# First Microsatellite Markers Developed from Cupuassu ESTs: Application in Diversity Analysis and Cross-Species Transferability to Cacao

**DOI:** 10.1371/journal.pone.0151074

**Published:** 2016-03-07

**Authors:** Lucas Ferraz dos Santos, Roberta Moreira Fregapani, Loeni Ludke Falcão, Roberto Coiti Togawa, Marcos Mota do Carmo Costa, Uilson Vanderlei Lopes, Karina Peres Gramacho, Rafael Moyses Alves, Fabienne Micheli, Lucilia Helena Marcellino

**Affiliations:** 1 Universidade Estadual de Santa Cruz (UESC), Departamento de Ciências Biológicas (DCB), Centro de Biotecnologia e Genética (CBG), Rodovia Ilhéus-Itabuna, km 16, 45662–900 Ilhéus-BA, Brazil; 2 Embrapa Recursos Genéticos e Biotecnologia, Brasília-DF, 70770–917, Brazil; 3 Cocoa Research Center, CEPLAC/CEPEC, 45600–970 Itabuna-BA, Brazil; 4 Embrapa Amazônia Oriental, 66095–903 Belém-PA, Brazil; 5 CIRAD, UMR AGAP, F-34398 Montpellier, France; Beijing Forestry University, CHINA

## Abstract

The cupuassu tree (*Theobroma grandiflorum*) (Willd. ex Spreng.) Schum. is a fruitful species from the Amazon with great economical potential, due to the multiple uses of its fruit´s pulp and seeds in the food and cosmetic industries, including the production of *cupulate*, an alternative to chocolate. In order to support the cupuassu breeding program and to select plants presenting both pulp/seed quality and fungal disease resistance, SSRs from Next Generation Sequencing ESTs were obtained and used in diversity analysis. From 8,330 ESTs, 1,517 contained one or more SSRs (1,899 SSRs identified). The most abundant motifs identified in the EST-SSRs were hepta- and trinucleotides, and they were found with a minimum and maximum of 2 and 19 repeats, respectively. From the 1,517 ESTs containing SSRs, 70 ESTs were selected based on their functional annotation, focusing on pulp and seed quality, as well as resistance to pathogens. The 70 ESTs selected contained 77 SSRs, and among which, 11 were polymorphic in cupuassu genotypes. These EST-SSRs were able to discriminate the cupuassu genotype in relation to resistance/susceptibility to witches’ broom disease, as well as to pulp quality (SST/ATT values). Finally, we showed that these markers were transferable to cacao genotypes, and that genome availability might be used as a predictive tool for polymorphism detection and primer design useful for both *Theobroma* species. To our knowledge, this is the first report involving EST-SSRs from cupuassu and is also a pioneer in the analysis of marker transferability from cupuassu to cacao. Moreover, these markers might contribute to develop or saturate the cupuassu and cacao genetic maps, respectively.

## Introduction

The cupuassu tree, *Theobroma grandiflorum* (Willd. ex Spreng.) Schum., belonging to the *Malvaceae* family, is a fruitful species native to the Amazon [1]–as the cacao tree (*Theobroma cacao* L.) whose seeds are used as raw material for chocolate production. The cupuassu tree is considered one of the main tree crops in the Amazon region [2,3], being economically important in Brazil, with great potential at international level due to the multiple uses of its fruit pulp and seeds. From the pulp, several products are manufactured, such as juices, ice creams, liquors, jams, jellies, creams and sweets [2, 3]. Cupuassu seeds have a high quality fat, composed mainly of oleic and stearic acid [4, 5], from which a product similar to chocolate, called *cupulate*, can be obtained [6–8]. Moreover, cupuassu received attention because of its proteolytic activity, useful in food industry [8], its antioxidant and cytotoxic activity, as well as its action in increasing glucose tolerance [9–11]. Due to its potential for the “chocolate” industry—particularly in the actual period of announced cacao beans and chocolate shortage [12, 13]–studies related to cupuassu species are increasing at molecular and breeding level [14–17]. Moreover, the genetic proximity of cupuassu with cacao—that has been thoroughly studied during the last 10 years [18–21]–allowed the transfer of data and technologies, as well as comparison for improvement of breeding programs related to different characteristics such as pulp/seed quality and disease resistance.

Considering that in Brazil, the main phytopathological problem that affects the *Theobroma* genus is the witches’ broom disease—caused by the hemibiotrophic basidiomycete *Moniliophthora perniciosa* [22]–the cupuassu breeding program should integrate the selection of lines that present both pulp/seed quality and resistance to this fungus. Such selection could be assisted by microsatellites (SSRs) markers that are short repeat motifs with high polymorphism due to indel mutation-type in one or more repeats [23]. SSRs distribution is considered as nonrandom across both coding and noncoding regions of genomic DNA, and some of these SSR structures are important for different cell function (e.g. gene transcription, chromatin organization, DNA replication, cell cycle), indicating that some of the SSR groups may not be neutral [23]. In plant genetics, the SSRs were preferred due to their high variability, abundance, multiallelic nature, reproducibility, polymorphism, transferability as well as their codominant inheritance, chromosome-specific location and wide genomic distribution [23–25]. SSRs, in many species, were widely used for genetic diversity studies, molecular mapping, molecular fingerprint and conservation strategies [26].

When these SSRs are identified in expressed sequence tags (ESTs), the selection of interesting plant genotypes could be quite efficient mainly because the markers are physically associated to coding regions and can enhance the evaluation of plant populations by enabling the variation assay in expressed genes with known function [27]. With the advent of low cost next generation sequencing (NGS) technologies, it is now possible to easily obtain thousands of ESTs that could be the main source for *in silico* SSR identification (then named EST-SSRs). Identification of EST-SSRs is also important in the study of different species from the same genus [28–32], in which gene function and biological processes could be conserved [24, 33] and may be related to the same responses to biotic and/or abiotic stresses. Therefore, the transferability of SSRs or EST-SSRs between species may support the idea of similar existing function, as well as to contribute to comparative genomics and diversity analysis [34–36].

For this reason, herein, we focused on: i) the identification and description of SSRs from new generation sequencing-obtained ESTs of cupuassu; ii) the analysis of the related EST function; iii) the validation of the SSRs on cupuassu genotypes with varied pulp quality and resistance to witches’ broom disease and diversity study in relation to both characteristics; iv) the transferability of cupuassu SSR to cacao genotypes. To our knowledge this is the first work involving EST-SSRs from cupuassu and is also a pioneer in the analysis of marker transfer from cupuassu to cacao.

## Material and Methods

### Plant material

Cupuassu genotypes used for EST-SSR validation were selected focusing on subsequent applications in breeding program for pulp quality improvement and/or witches’ broom disease resistance. Sixteen cupuassu genotypes from Embrapa Amazonia Oriental were used (Tables 1 and 2) in this study. Among them, fourteen were resistant to witches’ broom disease and two susceptible (Table 2; personal communication R.M. Alves). The genotypes 174 (Coari) and 1074, resistant and susceptible to witches’ broom disease, respectively, were the genitors of several of the progenies used in the breeding programs in Brazil (Table 1) [14]. For marker transferability analysis, three *Theobroma cacao* L. genotypes, from Ceplac (Bahia, Brazil) were used: two resistant, SCA6 and TSH 516, and one susceptible, ICS1. The TSH 516 genotype corresponds to the SCA6 x ICS1 cross [37].

**Table 1:** Origin of the cupuassu genotypes from the Brazilian breeding programs used in this study. AM: Amazon State from Brazil; AP: Amapá State from Brazil; PA: Pará State from Brazil.

Genotype	Original crossing(♀ x ♂)	♀ origin	♂ origin
32	174 x 186	Coari—AM	Codajás—AM
42	186 x 434	Codajás—AM	Muaná—PA
44	186 x 434	Codajás—AM	Muaná—PA
46	186 x 215	Codajás—AM	Manacapuru—AM
47	186 x 1074	Codajás—AM	Itacoatiara—AM
48	186 x 1074	Codajás—AM	Itacoatiara—AM
51	215 x 624	Manacapuru—AM	Santarém—PA
56	186 x 1074	Codajás—AM	Itacoatiara—AM
57	186 x 513	Codajás—AM	Oiapoque—AP
61	220 x 228	Manacapuru—AM	Manaus—AM
62	220 x 185	Manacapuru—AM	Codajás—AM
63	174 x 248	Coari—AM	Itacoatiara—AM
64	220 x 185	Manacapuru—AM	Codajás—AM
174	-	Coari—AM	**-**
186*	-	Codajás—AM	-
215	-	Manacapuru—AM	**-**
1074	-	Parintins—AM	-

* This genotype was used in some of the original crosses but was not used in the subsequent analysis presented in this work.

**Table 2:** Pulp characteristics and response to witches’ broom disease of the cupuassu genotypes used in this study. ATT: titratable acidity; R: resistant; S: susceptible; SST: total soluble solids; ST: total solids.

Genotype	Physico-chemical characteristics of pulp						Response to witches’ broom disease
	pH	SST	ATT	Humidity	ST	SST/ATT	
32	3.7	12.2	1.7	82.3	17.7	7.0	R
42	3.6	12.7	1.7	84.0	16.0	7.5	R
44	3.3	16.0	2.4	83.9	16.1	6.7	R
46	3.6	11.3	1.6	85.4	14.6	6.9	R
47	3.9	10.3	1.2	88.1	11.9	8.4	R
48	3.2	11.0	2.2	86.0	14.0	5.0	R
51	3.4	10.6	1.6	85.1	14.9	6.7	R
56	3.4	13.0	2.2	83.2	16.8	5.8	R
57	3.7	12.5	1.4	84.9	15.1	8.8	R
61	3.4	12.1	2.7	86.5	13.5	4.6	R
62	3.5	11.7	1.9	86.1	13.9	6.3	S
63	3.9	12.5	0.9	85.1	14.9	13.3	R
64	4.0	11.3	1.1	86.3	13.7	10.6	R
174	3.5	13.1	1.5	83.9	16.1	8.7	R
215	3.5	14.4	2.2	80.4	19.6	6.6	R
1074	3.5	10.7	1.8	86.0	14.0	5.9	S

### Cupuassu pulp quality analyses

For the pulp quality analyses, five cupuassu fruits were harvested from three different plants (n = 15) for each of the sixteen cupuassu genotypes described (Table 2). For the evaluation of the pulp characteristics (°Brix, acidity, humidity and pH), 20 g of pulp from each fruit were collected and analyzed as previously described [38]. The Brix was determined using a refractometer PR-101 (ATAGO). The total acidity, expressed in citric acid percentage, was determined by titration using 0.1 N NaOH. The pH was determined using a Horiba F-21 pH-meter. For the determination of humidity, the samples were oven dried at 105°C until weight stabilization.

### EST sequencing, EST-SSR identification, and primer design

**Fig 1:**
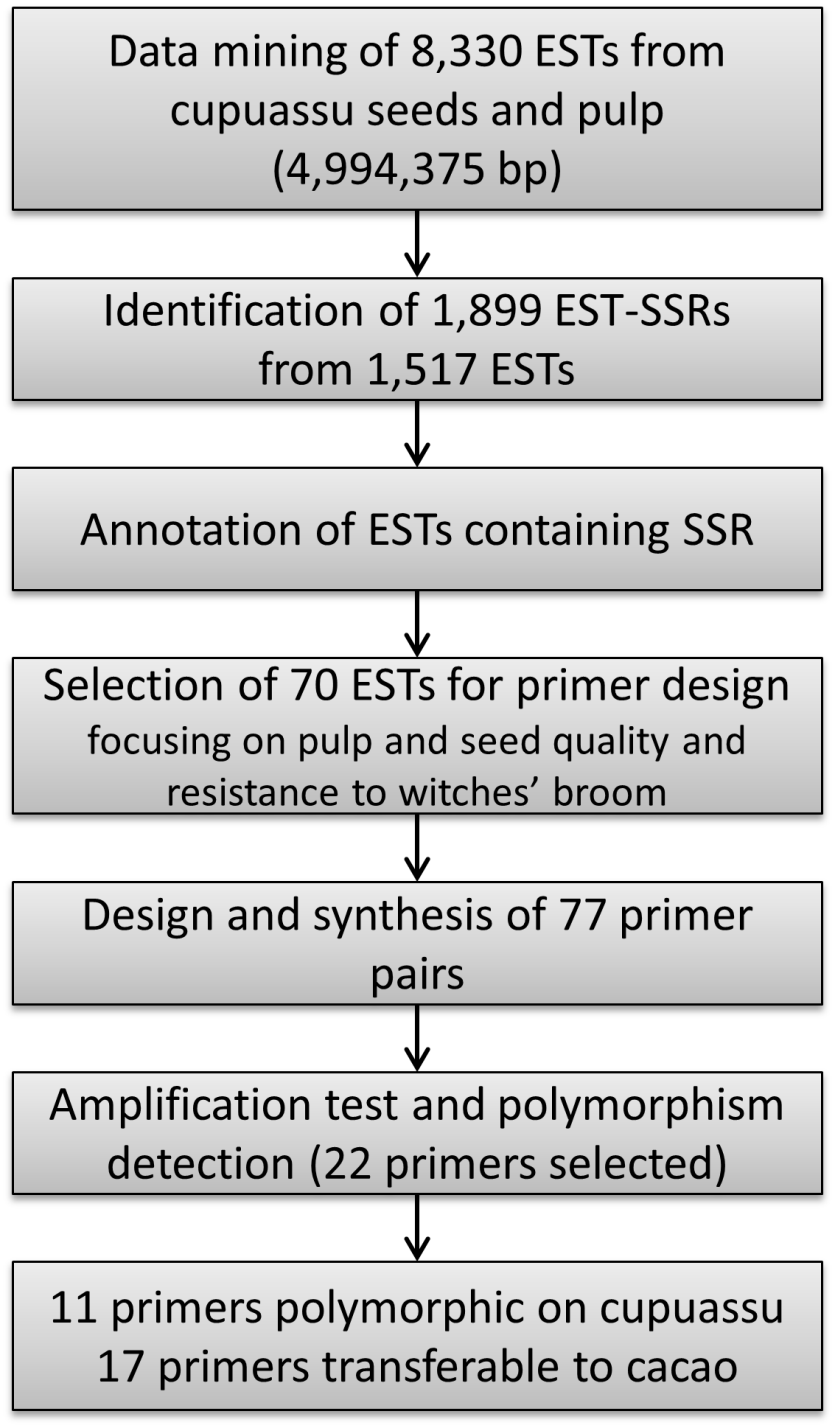
Scheme used for data mining and development of EST-SSRs from cupuassu seeds and pulp.

In this study, pulp and seed of the cupuassu genotype (Coari 174) (*Theobroma grandiflorum* [Willd. ex Spreng.] Schumm.; see also Plant material) grown at the experimental station of Embrapa Amazonia Oriental (Belém, Pará, Brazil) were sequenced using the 454 platform / Roche Applied Sciences. The raw sequences were trimmed and assembled using the est2assembly [39] and Mira [40] software resulting on 8,330 contig sequences. The sequences are available on the cupuassu restricted databases at http://lbi.cenargen.embrapa.br/cupuacu/. The ESTs were screened for the presence of SSRs using the MISA software [41] according to the following criteria: i) nucleotide motif/minimum number of repeats of 1/10, 2/6, 3/4, 4/2, 4/3, 5/3, 6/5, 7/2, 8/2 and 9/2; and ii) maximum difference between two SSRs of 100 bp. For putative function determination and annotation, EST sequences containing SSRs were compared with the public sequence database using BLASTX against the non-redundant (NR) protein database (http://www.ncbi.nih.gov/BLAST/; [42]) and with the cacao protein-coding sequence database (http://cocoagendb.cirad.fr; [19]). Alignments showing similarity with an expected value (e-value) ≤1.10^−7^ were considered significant. The GO annotation for the ESTs containing SSRs were performed using Gene Ontology Consortium tools (http://www.geneontology.org/) [43] and then manually inspected and classified as previously described [44]. The primers were designed using the Primer3 software (http://primer3.wi.mit.edu/) according to the following criteria: i) amplicon size of 100–300 bp; ii) primer length of 17–23 bases; iii) melting temperature of 56–60°C; and iv) GC content of 40%-60%. See Fig 1 for the general scheme of data mining for SSR identification.

### Location of the EST-SSR in relation to the coding sequence of the cDNA

The open reading frame (ORF) of the 70 chosen ESTs was determined using the ORF Finder program (http://www.ncbi.nlm.nih.gov/gorf/gorf.html) and by comparison with cacao genome (http://cocoagendb.cirad.fr; [19]), and the SSR was localized in relation to the ORF. The possible locations were: in the 5’ untranslated region (5’UTR), in the ORF region, or in the 3’ untranslated region (3’UTR). In some cases, due to the EST sequence length or quality, it was not possible to clearly determine the ORF and consequently the location of the SSR.

### DNA extraction, PCR amplification and electrophoresis conditions

Cupuassu and cacao DNA were extracted from young leaves as previously described [45] and quantified using Nanodrop 2000 (Thermo Scientific). The optimization phase of the 77 primers designed was performed using the cupuassu genotypes 174 and 1074 (see Plant material). For the optimization phase, PCR was performed in 13 μl containing 7.5 ng of DNA, 0.25 mmol.l^-1^ of each dNTPs, 10 mmol.l^-1^ of Tris-HCl pH 8.3, 50 mmol.l^-1^ of KCl, 2 mmol.l^-1^ of MgCl_2_, 0.2 μmol.l^-1^ of each primer, and 1U of Taq DNA polymerase (Phoneutria). Amplifications were performed using the Mastercycler PCR 5333 thermocycler (Eppendorf), using the following conditions: 96°C for 2 min, 30 cycles at 94°C for 1 min, 58°C for 1 min, 72°C for 1 min, and a final extension step at 72°C for 7 min. Amplified fragments were analyzed by electrophoresis on 4% denaturing TBE acrylamide gels. Polymorphism was evaluated by scoring the SSR bands. When comparing the genotypes, the presence or absence of a determined band (similar size) indicated similarity or dissimilarity between genotypes, respectively. The 10-bp molecular marker (Invitrogen) was used as a reference to score the bands. For the confirmation of the polymorphic primers, the amplifications were made on the 16 cupuassu genotypes (Table 1). PCR was performed as described above, excepted for the primers that were labelled with the M13 tail, and with the increase in the reaction of 0.2 μmol.l^-1^ of M13 primer labelled with NEDTM fluorescence, and 10 μmol.l^-1^ of 6-FAM. The amplification products were analyzed on the ABI3500 sequencer (Applied Biosystems) using GeneScan^™^ 500 LIZ^™^ dye (Life Technologies) as internal size standard. The allele size was defined using the GeneMarker software. The transferability of the developed EST-SSR primers was carried out by cross-species amplification on genomic DNA of three *T*. *cacao* genotypes (SCA6, ICS1, TSH516) using the same PCR and electrophoretic conditions (4% denaturing TBE acrylamide gel) as described above.

### Sequencing of amplicons for marker confirmation

PCR amplifications were carried out in 20 μl reaction volume containing PCR buffer 1X (Invitrogen), 0.375 mM of each primer (see S1 Table), 10 ng/μl of cupuassu DNA (genotypes 1074 and 174) and 0.5 U of Taq polymerase (Invitrogen). Thermocycling conditions consisted of an initial melt at 95°C for 5 min followed by 28 cycles of 95°C for 30 s, 58°C for 90 s, 72°C for 30 s and a final extension step of 72°C for 10 min. All amplifications were performed in a MyCycler thermocycler (Bio-Rad Laboratories). PCR amplification reactions were checked on electrophoresis on 1.8% agarose gel stained with Gel-red I (Invitrogen). PCR products were cleaned with ExoSap-IT (USB) according to the manufacturer’s instructions. Sequencing was performed on the ABI3100 equipment (Applied Biosystems) at Ceplac (Bahia, Brasil). The confirmation of the SSR marker was based on the comparison of number of repeated sequences of each allele among the different genotypes.

### Genetic diversity and statistical analysis

The amplified SSR DNA bands representing different alleles were scored on the different genotypes. The genetic diversity parameters were assessed in terms of observed number of alleles (Na), observed heterozygosity (Ho), and expected heterozygosity (He) using the Genetic Data Analysis software [46]. Polymorphic information content (PIC) was obtained for each locus as previously described [47] and null alleles were examined using Micro-checker software, v.2.2.3 [48]. Factorial Component Analysis (FCA) was made with the GENETIX software [49]. Correlation test between molecular data and pulp quality or resistance to witches’ broom disease was realized using the SAS program [50].

### *In silico* comparison of *Theobroma grandiflorum* loci with *Theobroma cacao* var. Criollo

For cupuassu/cacao loci comparison, *T*. *grandiflorum* ESTs were compared to cacao genome var. Criollo (CacaoGenDB; http://cocoagendb.cirad.fr) using the blastn tool of the CacaoGenDB configured with the following parameters: blast against gene sequences (including UTRs and introns) and expected e-value of 1.10^−10^ [19]. Specific repeat motifs observed in cupuassu loci were searched in the corresponding region of the cacao sequence (ORF or UTRs). Primers used for SSR analysis in cupuassu and for transferability study in cacao were also blasted on the cacao genome using the specific Primer Blaster tool from CocoaGenDB, with an acceptability of up to three mismatches. Each cupuassu EST and the corresponding cacao sequences were compared and aligned using the Clustal Omega program (http://www.ebi.ac.uk/Tools/msa/clustalo/).

## Results

### Frequency, distribution and function of SSRs in cupuassu ESTs

**Fig 2:**
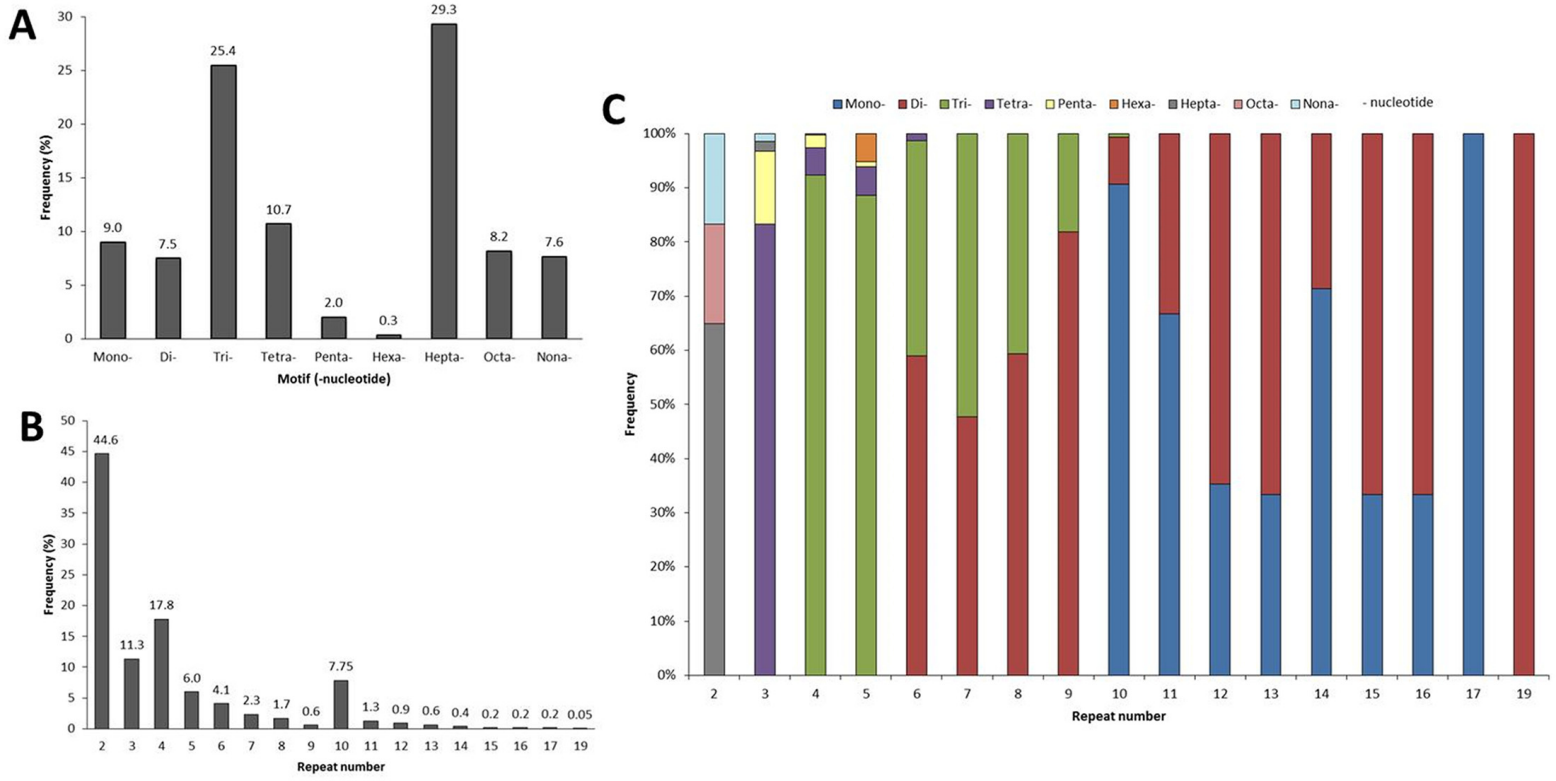
Frequency of 1899 EST-SSRs with different motifs and repeat number. **A.** Frequency of EST-SSRs with mono-, di-, tri-, tetra-, penta-, hexa-, hepta-, octa- and nonanucleotide motifs. **B.** Frequency of EST-SSRs with 2 to 19 repeat motifs. **C.** Frequency of mono-, di-, tri-, tetra-, penta-, hexa-, hepta-, octa- e nonanucleotide motifs for each repeat number category.

**Fig 3:**
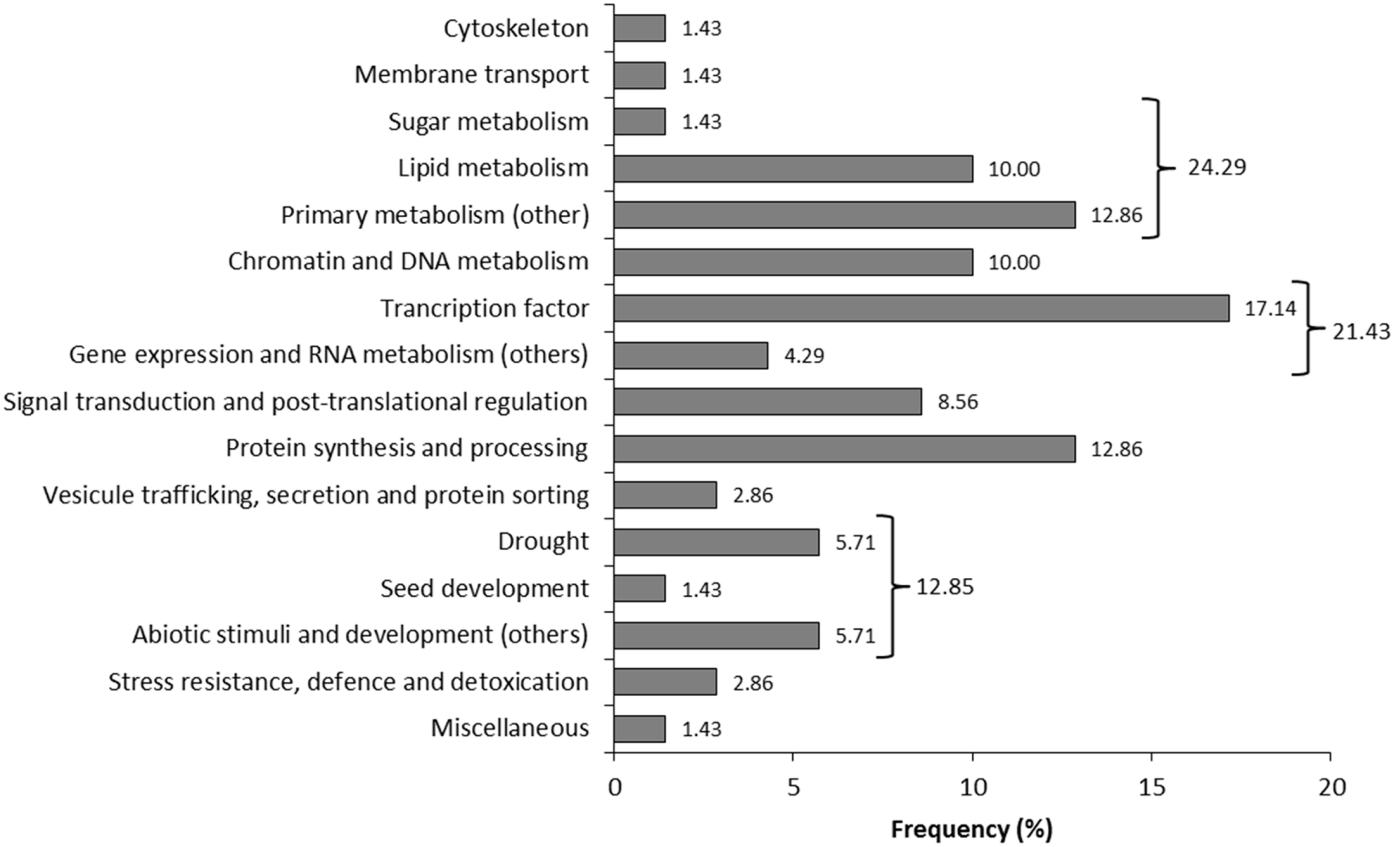
Functional annotation of the 70 ESTs selected for primer design. The frequency of each category was indicated.

**Fig 4:**
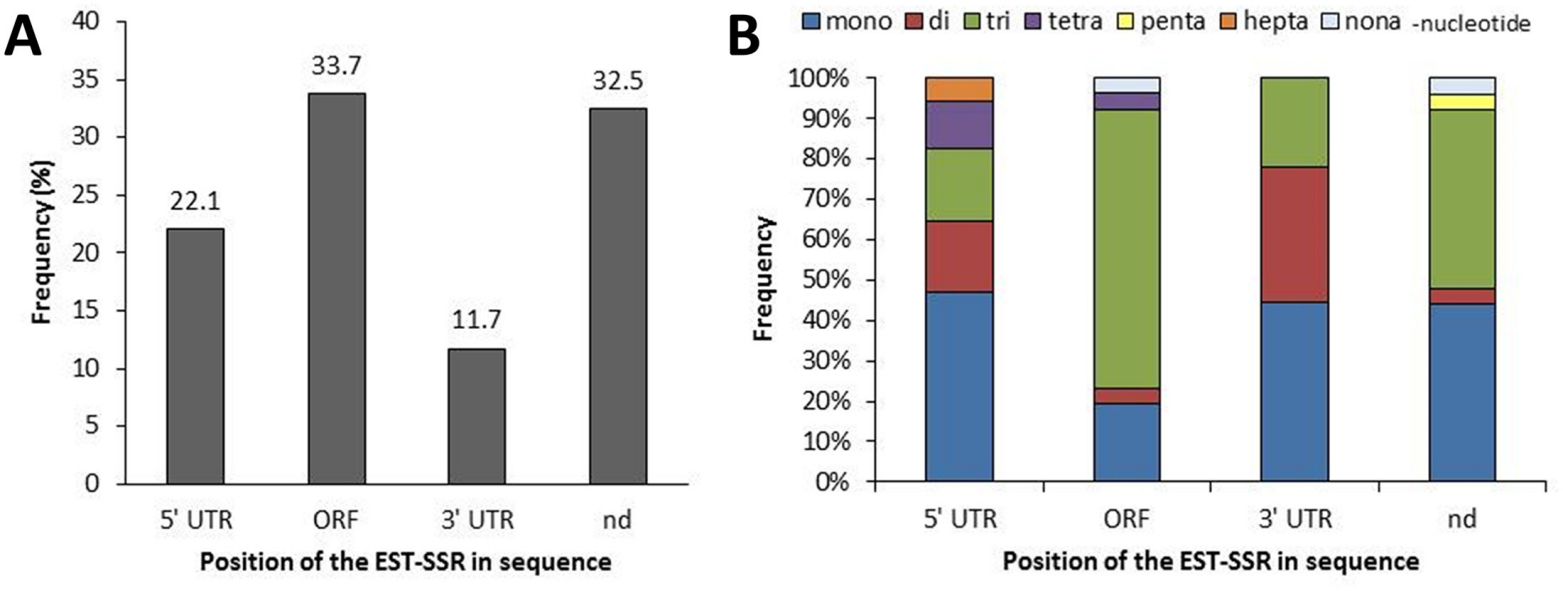
Frequency of the 77 selected EST-SSRs in the different sequence region. **A.** Frequency of the EST-SSRs according to sequence structure. **B.** Frequency of repeat motif in each sequence region. ORF: open reading frame; UTR: untranslated region, nd: undetermined.

Among a total of 8,330 EST sequences from cupuassu (pulp and seeds), 1,517 ESTs containing 1,899 SSRs were identified (Fig 1). Two-hundred and eighty ESTs contained more than one SSR (data not shown). From the 1,899 EST-SSRs, nine types of motifs were identified: mononucleotides (9%), dinucleotides (7.5%), trinucleotides (25.4%), tetranucleotides (10.7%), pentanucleotides (2.0%), hexanucleotides (0.3%), heptanucleotides (29.3%), octanucleotides (8.2%) and nonanucleotides (7.6%) (Fig 2A). Within the mononucleotides, A and T were the most frequents (4.53% and 4.27%, respectively); within the dinucleotides, AT, AG and TC were the most frequent (1.58%, 1.47% and 1.42%, respectively), followed by the GAA (1.32%), AAG (1.26%), CTT (1.21%), TCT and TTC (1.16%) trinucleotides (S2 Table). The tetra-, penta-, hexa-, hepta-, octa- and nonanucleotides presented similar low frequency, mostly <0.7% (S2 Table). The motifs were found with a minimum and maximum of 2 and 19 repeats, respectively (Fig 2B, S2 Table). From the 1,517 EST-SSRs, 44.6, 17.8, 11.3 and 7.75% presented 2, 4, 3 and 10 repeats, respectively (Fig 2B). The other highest categories were 5, 6, 7, 8 and 11 repeats (6%, 4.1%, 2.3%, 1.7% and 1.3%, respectively) followed by the lowest categories (12, 9, 13, 14, 15, 16, 17, 19 repeats, all <1%; Fig 2B). The mono-, di- and trinucleotides were the most repeated (repeat number 6 to 19; Fig 2C). Tetranucleotides were repeated three to six times, pentanucleotides three to five times, hexanucleotides five times, hepta- and nonanucleotides two to four times and octanucleotides two times (Fig 2C). From the 1,517 ESTs containing SSRs, 70 ESTs were selected based on their functional annotation focusing mainly on sequences potentially involved in the pulp and seed quality characteristics or development, but also in other potentially interesting regulating sequences (e.g. transcription factors) or sequences related to resistance (Fig 3; S1 Table). From these, 24.29% were related to primary metabolism, including lipid (10%) and sugar metabolisms (1.43%), 21.43% were related to gene expression and RNA metabolism, 12.86% to protein synthesis and processing, 12.85% to drought, seed development and other abiotic stresses, 10% to chromatin and DNA metabolism, 8.57% to signal transduction and post-translational regulation, 2.86% to stress resistance, defense and detoxification. The other categories corresponded to 7.14% (Fig 3). The 70 selected ESTs contained 77 SSRs for which primers were designed (Fig 1; S1 Table). Considering these 77 SSRs in relation to the coding sequence position, 33.7% were found in the ORF, 22.1% in the 5’UTR and 11.7% in the 3’UTR; for 32.5% of the SSRs, the localization in relation to the ORF was not possible (Fig 4A). The 5’UTR contained mono-, di-, tri-, tetra- and heptanucleotides, while the 3’UTR contained mono-, di-, and trinucleotides (Fig 4B). The ORF mainly contained tri- and mononucleotides, followed by di-, tetra- and nonanucleotides (Fig 4B).

### Polymorphism detection in cupuassu genotypes and diversity analysis

**Fig 5:**
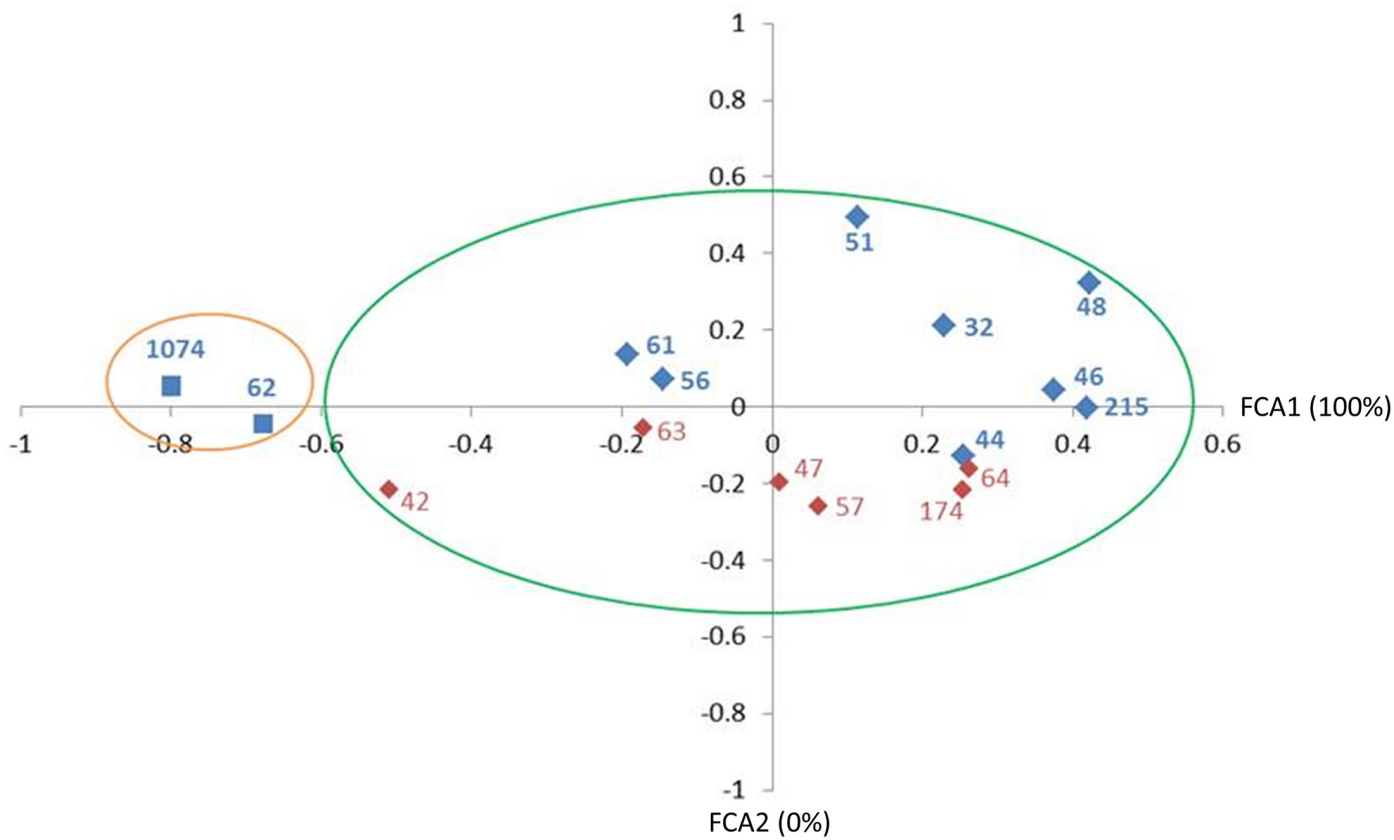
Distribution of the 16 genotypes of cupuassu (resistant and susceptible, described in Table 1) based on allele frequencies using eleven polymorphic SSR markers (Table 3) and pulp quality characteristics (Table 2). The susceptible cupuassu genotypes were indicated by squares (62 and 1074); the other ones were susceptible and indicated by diamonds. The cupuassu genotypes with SST/ATT parameter >7.0 were indicated in red; those with SST/ATT parameter ≤ 7.0 were indicated in blue. Orange circle separated the susceptible genotypes to the resistant ones (green circle).

From the 77 SSRs selected, 22 were pre-selected, and finally 11 were confirmed as polymorphic (Fig 1, Table 3) when tested in the cupuassu genotypes described in Table 1. The number of alleles per EST-SSR ranged from 2 to 6 with an average of 3.18. The observed heterozygosity (H*o*) values ranged from 0 to 0.88 with an average of 0.51, and the expected heterozygosity (H*e*) values ranged from 0.083 to 0.76 with an average of 0.5. PIC values of the EST-SSR ranged from 0.32 to 0.7 with an average of 0.5 (Table 3). Eight of the SSRs were located in the ORF of the corresponding EST (72.7%) and 3 in the 5’UTR (27.3%) (Table 3). These 11 markers used in the genetic diversity analysis revealed a clusterization according to the resistance *vs* susceptibility of the cupuassu genotypes; the susceptible genotypes 62 and 1074 were discriminated from the others (Fig 5). Interestingly, the genotype 1074, that present the higher deviation, also came from a different geographic origin (Parintins—AM; Table 1) than the other genotypes. The diversity analysis also showed a tendency of genotype clusterization according to the SST/ATT parameter, discriminating into two groups: i) SST/ATT ≤ 7.0 (genotypes 32, 44, 46, 48, 51, 56, 61, 62, 215, 1074); ii) SST/ATT > 7.0 (genotypes 42, 47, 57, 63, 64 and 174) (Fig 5, Table 2).

**Table 3:** Characteristics of eleven microsatellite loci derived from cupuassu ESTs analyzed in cupuassu genotypes (see Table 1). He: expected heterozygosity; Ho: observed heterozygosity; Na: number of alleles; PIC: polymorphic information content. For all the locus/primers, the annealing temperature was 58°C.

Locus name	Putative gene function	Organism	Repeat motif	Marker validation					Position in sequence
				Amplicon sizerange (bp)	Na	He	Ho	PIC	
c2723^a^,^b^	AML1	*Citrus unshiu*	(CAT)_6_	265–280	4	0.52	0.63	0.44	ORF
c5718^a^,^b^	Disease resistance protein RPM1	*Ricinus communis*	(CTC)_5_	258–267	4	0.65	0.44	0.57	ORF
c70^a^,^b^	Ethylene-responsive element-binding factor	*Gossypium hirsutum*	(AGA)_6_	135–141	2	0.40	0.4	0.32	5’UTR
c3202	CBL-interacting serine/threonine-protein kinase	*Ricinus communis*	(GAA)_6_	258–297	3	0.58	0.85	0.50	ORF
c3202B^a^,^b^	CBL-interacting serine/threonine-protein kinase	*Ricinus communis*	(GAA)_6_	239–293	6	0.50	0.50	0.45	ORF
c733	Ubiquitin-activating enzyme E1	*Ricinus communis*	(CAA)_2_(CAA)_2_	88–94	2	0.52	0.67	0.43	ORF
c180^a^	Eukaryotic translation initiation factor 5A isoform I	*Hevea brasiliensis*	(GA)_8_	200–212	2	0.20	0.00	0.70	5’UTR
c193B	DNA-binding protein	*Vitis vinifera*	(GAT)_4_	297–315	2	0.52	0.88	0.38	ORF
c203B^a^,^b^	Nuclear acid binding protein	*Ricinus communis*	(TTGACCCGC)_2_	157–211	2	0.08	0.08	0.50	ORF
c339^a^	Transcription factor	*Ricinus communis*	(AAAT)_2_	137–147	4	0.60	0.50	0.40	ORF
c431B^a^,^b^	Ribosomal protein S14	*Nicotiana tabacum*	(A)_10_(TA)_8_(TA)_6_(TA)_10_	231–276	4	0.76	0.64	0.40	5’UTR
Mean					3.18(35)*	0.50	0.51	0.50	-

^a^ Transferable to cacao genotypes SCA6, ICS1 and TSH516 –see also Table 4

^b^ Polymorphic in cacao genotypes SCA6, ICS1 and TSH516 –see also Table 4

* Total number of alleles

### Transferability of EST-SSRs

**Fig 6:**
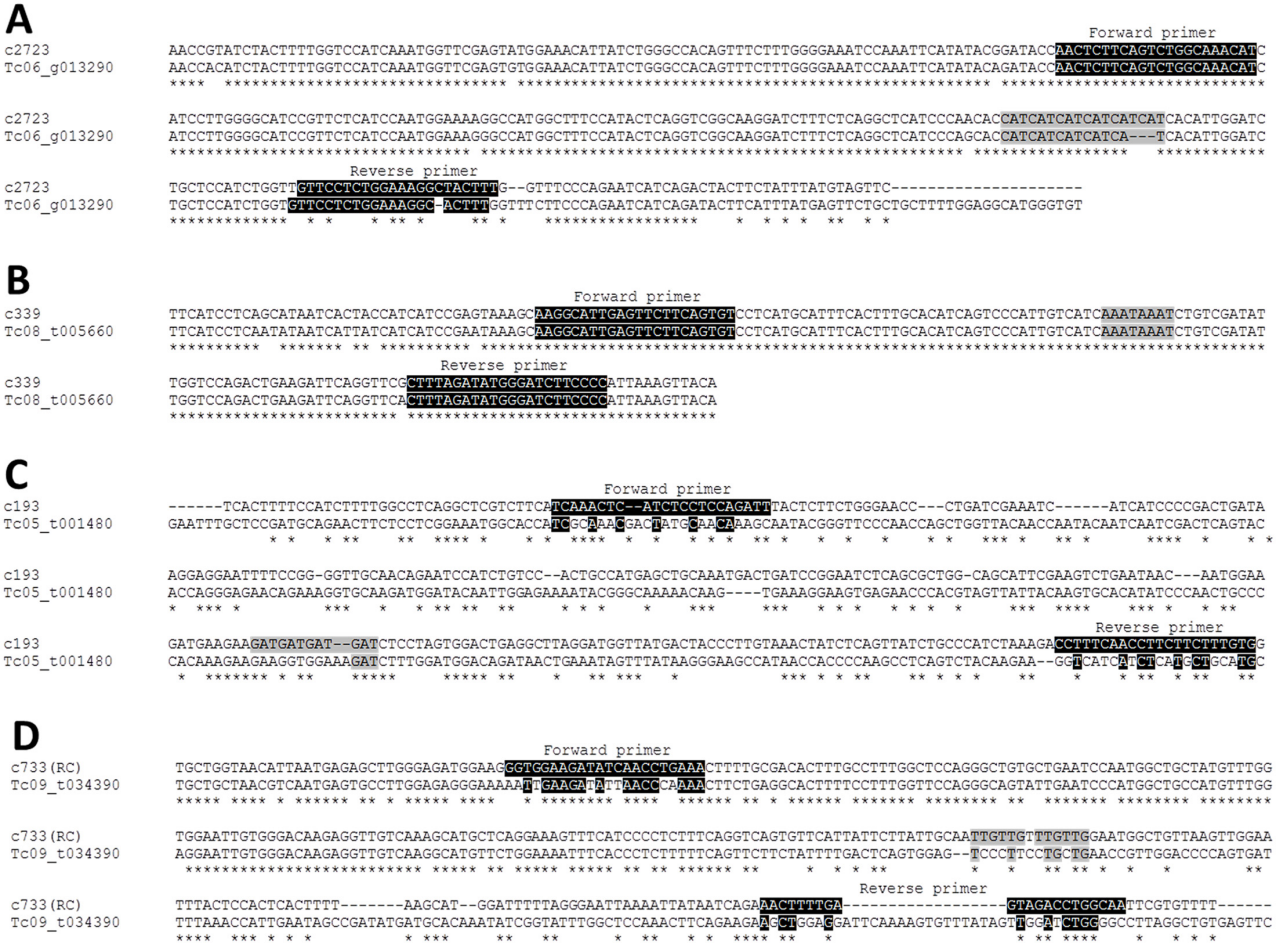
Alignment between cupuassu ESTs and genomic cacao sequence (Criollo variety, see also Table 5) showing the different possible transferability situations. **A.** Transferable primers and polymorphic locus. **B.** Transferable primers and non-polymorphic locus. **C.** Untransferable primer and polymorphic locus. **D.** Untransferable and non-polymorphic locus.

The transferability of the cupuassu EST-SSRs to *T*. *cacao* was analyzed by cross-species amplification. From the 22 pre-selected EST-SSRs (polymorphic or not in cupuassu; Fig 1), 17 amplified cacao DNA, which corresponds to a transferability rate of 77% (Table 4). The amplifications were within the expected size, and 14 of the 17 cupuassu SSRs were polymorphic in cacao (Table 4). From the 11 EST-SSRs polymorphic in cupuassu, 8 were transferable to cacao and 6 were also polymorphic in this species (Tables 3 and 4). The 11 polymorphic locus of cupuassu were also compared to the cacao genome database (cacao var. Criollo) and several homolog sequences were encountered (Table 5). Eight cupuassu loci presented polymorphism when compared to cacao: six of them presented the same repeat motif, but with less repeats (c2723, c5718, c70, c180, c193B, c203B) for at least one homolog sequence, and 2 of them did not present the repeat motif (c3202/3202B, c733; Table 5). Two loci showed the same motif/repeat number in cupuassu and cacao (c339, c431B; Table 5). The *in silico* analysis showed that some primers were transferable allowing the identification of a polymorphic locus (e.g. c2723; Fig 6A, Tables 4 and 5). Some primers were transferable but the locus was non-polymorphic (e.g. c339; Fig 6B, Tables 4 and 5). The two other situations corresponded to primers that were not able to amplify the cacao gene, whatever if the locus was polymorphic or not (e.g. c193, c733; Fig 6C, Tables 4 and 5). It is interesting to note that some loci were transferable to cacao but presented different polymorphism depending on the cacao variety analyzed: for example, the c431B locus is polymorphic in SCA6/ICS1/TSH516 varieties (Table 4) but did not presented potential polymorphism in the *in silico* analysis using the Criollo variety (Table 5).

**Table 4:** Transferability analysis of the EST-SSRs from cupuassu to *Theobroma cacao*.

Locus	Cacao genotypes			Observed size range (bp)
	SCA6	ICS1	TSH516	
c2723*	153	159	156	153–159
c8207	-	166	-	166
c5718*	117/126	117/126	117	117–126
c70*	204	204	207	204–207
c3202B*	170	173	173	170–173
c180*	104	104	-	104
c203B	130	130	139	130–139
c1251	132	132	130	130–132
c339*	-	-	115	115
c2763	154	158	158	154–158
c5718B	174	170	174	170–174
c431B*	156	153	153	153–156
c431C	150	146	156	146–156
c432	136	146	146	136–146
c8097	188	190/200	190	188–200
c618	-	-	156/164	156–164
c1295	152	156	156	152–156

*Polymorphic in cupuassu (see also Table 3)

**Table 5:** Comparison of the 11 polymorphic *Theobroma grandiflorum* loci (see Table 4) with the cacao genome database (*Theobroma cacao* var. Criollo; CocoaGenDB—http://cocoagendb.cirad.fr). Chr.: chromosome; F: forward primer; R: reverse primer; (-) indicates no specific repeat type in the corresponding gene region.

*Theobroma grandiflorum*				*Theobroma cacao*					
Locus name	Sequence size (bp)	Repeat motif(n° of repeat)	Position in sequence	E-value	Identity (%)	Locus ID	Chr.	Putative gene function	Repeat motif(n° of repeat)
c2723	317	(CAT)6	ORF	1E-129	95.9	Tc06_g013290	Tc06	AML1	(CAT)_5_
c5718	684	(CTC)5	ORF	0	87.14	Tc07_p005960	Tc07	Disease resistance protein RPS2	(CTC)_4_
c70	761	(AGA)6	5’UTR	0	96.18	Tc09_p008690	Tc09	Profilin-1	(AGA)_3_
				2E-21	84.9	Tc09_p004370	Tc09	Profilin-1	(AGA)_1_
c3202	452	(GAA)6	ORF	1E-175	91.85	Tc04_p030640	Tc04	CBL-interacting serine/threonine-protein kinase 1	-
c3202B	452	(GAA)6	ORF	1E-175	91.85	Tc04_p030640	Tc04	CBL-interacting serine/threonine-protein kinase 1	-
c733	1880	(CAA)2(CAA)2	ORF	0	96.93	Tc09_p034380	Tc09	Ubiquitin-activating enzyme E1 2	-
				1E-180	85.22	Tc09_p034390	Tc09	Ubiquitin-activating enzyme E1 2	-
c180	881	(GA)8	5’UTR	0	94.14	Tc02_p032650	Tc02	Eukaryotic translation initiation factor 5A-2	(GA)6
				2E-34	88.18	Tc03_p010560	Tc03	Eukaryotic translation initiation factor 5A	(GA)1
				4E-26	86.21	Tc09_p002800	Tc09	Eukaryotic translation initiation factor 5A	(GA)2
c193B	1974	(GAT)4	ORF	1E-15	89.47	Tc05_p005710	Tc05	WRKY transcription factor 44	-
				4E-12	94	Tc07_p002020	Tc07	WRKY transcription factor 20	-
				3E-47	87.21	Tc05_p001480	Tc05	Predicted protein	(GAT)1
				0	96.92	Tc09_p034740	Tc09	DNA-binding protein	(GAT)2
				7E-11	86.25	Tc07_p000190	Tc07	WRKY transcription factor 2	(GAT)1
c203B	1507	(TTGACCCGC)2	ORF	0	97.1	Tc06_p019000	Tc07	Hypothetical protein	(TTGACCCGC)1
				1E-48	88.64	Tc06_p008790	Tc06	Nuclear acid binding protein, putative	-
c339	2695	(AAAT)2	ORF	9E-42	93.85	Tc04_p020630	Tc04	LRR receptor-like serine/threonine-protein kinase GSO2	-
				0	97.19	Tc08_p005660	Tc08	Transcription factor bHLH145	(AAAT)2
				6E-12	82.31	Tc02_p010840	Tc02	Uncharacterized protein	-
c431B	2301	(A)10(TA)8(TA)6(TA)10	5’UTR	5E-52	96.24	Tc05_p000610	Tc05	Ribosomal protein S4, mitochondrial	-
				2E-11	100	Tc05_p000830	Tc05	Hypothetical protein	-
				0	99.15	Tc05_p001190	Tc05	Hypothetical protein	(A)10(TA)8(TA)6(TA)6
				1E-37	94.17	Tc05_p001180	Tc05	Cytochrome b	-
				0	99.34	Tc05_p001200	Tc05	Ribosomal protein S14, mitochondrial	-
				8E-11	100	Tc05_p000730	Tc05	Hypothetical protein	-
				3E-13	90.62	Tc01_p014050	Tc01	Uncharacterized protein	-

## Discussion

In this article we obtained and analyzed a large number of ESTs from *Theobroma grandiflorum* (cupuassu) with the objective to identify new SSR markers useful for marker assisted selection in cupuassu with respect to both quality and resistance to witches’ broom disease. Both of these characteristics are important from a practical point of view for increasing the development of *cupulate* production or pulp-derived products, as an alternative to chocolate production declared in crisis [12, 13]. Moreover, the cupuassu breeding program needs the insertion of new markers for genetic fine mapping and selection of genome regions specifically involved in quality and/or resistance, in order to complement previous genetic analysis of cupuassu population [14, 15, 17]. Here we obtained SSR markers from NGS ESTs of cupuassu genotypes with different levels of resistance to witches’ broom disease and pulp quality. It is important to highlight that we produced the first EST database from cupuassu as well as the first EST-SSRs for this species. In cacao, more than 200,000 ESTs from different plant genotypes and organs submitted or not to different biotic and abiotic stresses [18, 44, 51–54], and more than 2,000 SSRs (whose 1631 [81%] were EST-SSRs) were already obtained (S3 Table) whereas in cupuassu, only genomic SSR were previously found (unpublished data, R.M. Alves). Furthermore, ESTs for use in molecular studies related to pulp or bean quality from the *Theobroma* genus are rare [18, 53].

Under these conditions, our results are highly relevant due to the large amount of ESTs generated (8,330) as well as the functional data associated to some of the EST-SSR identified (Fig 3). SSRs were detected in 18% of the ESTs analyzed (Fig 1), which corresponds to a high frequency comparing to data produced from other crops [24, 55, 56] with similar technical approaches (e.g. NGS, Misa analysis). Here, the highest proportions of EST-SSRs identified were hepta- and trinucleotides (29.3% and 25.4%, respectively; Fig 2A). Trinucleotides were generally considered as the most abundant class of SSRs in plant ESTs [27, 55–57] but other works also indicated dinucleotides [33, 58]. Since the addition or deletion of three nucleotides within translated regions usually does not affect the ORFs, it is not uncommon to detect a high abundance of these repeat motifs in EST-SSRs [59, 60], as we observed in our results (Fig 4B). But generally, it is accepted that the abundance of one or other SSR class may be due to the search criteria used for EST mining [26, 58, 61]. Nevertheless, the search criteria used for EST mining influences the frequency of the repeat number of the SSRs motifs; here the most frequent repeat number were 2, 4, 3 and 10 (44.6%, 17.8%, 11.3% and 7.75%, respectively; Fig 2B). Moreover, the SSRs containing the highest repeat numbers (10 to 19) were also the ones that contained exclusively mono- and dinucleotides (Fig 2C), while the SSRs with the lowest repeat numbers (2 to 6) contained larger motifs (tetra- to nonanucleotides; Fig 2C).

From the 1,899 EST-SSRs identified, 77 were tested as to their polymorphism in 16 cupuassu genotypes and 11 were polymorphic (Table 3). The PIC values (average 0.5; Table 3) observed here was closed to the ones found in cacao and cupuassu studies using genomic SSR [14, 62]. Such polymorphism was associated to genetic diversity of cupuassu according to the resistance parameter (characteristic that better discriminated the cupuassu genotypes) and, to a lesser extent, to SST/ATT parameter (Fig 5). The ATT data found in our study were consistent with the results obtained in other evaluations [63, 64], and 13 of the 16 genotypes studied (81%) presented ATT values higher than the minimum required (1.5; Table 2) [65]. The pH of cupuassu genotypes used here also showed values closed to those observed in other studies [63, 64, 66, 67] and all the genotypes (100%) presented values higher as to the required limit for good cupuassu quality (2.6; Table 2) [65]. The SST content also were consistent with other studies [63] and higher to the required limit [65] (Table 2); it is important to note that the harvesting period could influence the pulp quality as observed in other analyses where the SST values were lower than the expected values [64, 67]. Genotypes 63 and 64 showed the highest SST/ATT and for this reason may be considered as good candidate for breeding programs (Table 2). Generally, these data suggested that the cupuassu germplasm collection, as well as the cupuassu breeding program, generated material with high genetic variability related to pulp quality, and that the marker found here could be used for subsequent analysis of new crosses for cupuassu population and potentially for use in other *Theobroma* species.

Because EST-SSRs are generated from coding and expressed sequences, which are generally well conserved between species, the possibility to find conserved primers flanking the repeats—and possibly polymorphic—motifs, is high [26, 41]. Here we observed *in vitro* and *in silico* marker transferability between cupuassu and different varieties of cacao (resistant and susceptible to witches’ broom disease; Tables 4 and 5). Generally, the *in silico* analysis confirmed the *in vitro* results, and different transferability situations were observed (Table 5 and Fig 6). Transferability requires not only polymorphism between cuapuassu and cacao sequences, but also good primer design, able to amplify the polymorphic regions (Fig 6A). Therefore, the availability of the cacao genome and the study of the family of genes with interesting function can help to design primers able to amplify—and consequently to be efficiently transferred—between different species from the same genus. It is important to note that we report the first cupuassu-cacao marker transferability; whereas only a few studies of transferability between the two *Theobroma* species have been already reported and always from cacao to cupuassu [38, 68]. The first report used cacao markers previously developed [69] (S3 Table) to define the natural mating system of *Theobroma grandiflorum* in its putative center of diversity [38] while the second specifically deals with marker transferability from cacao to cupuassu [68]. The polymorphism rate calculated in these studies was lower (43.8%; Alves et al., 2006) than the one obtained here from EST-SSRs (77%; Fig 1, Table 4). Generally, in the work presented here we obtained a higher transferability (77%) than presented in other tests regarding marker transferability between correlated species [31, 35, 70]. The success of transferability between species as observed for coffee [71], rice [70], bananas [72], barley [73] and gerbera [74] is due to saving time and costs in the development of new markers.

## Conclusion

Here we obtained the first EST-SSRs from cupuassu. These markers were polymorphic in cupuassu and allowed diversity analysis of the studied genotypes, mainly in relation to pulp quality. Moreover, these markers were transferable to cacao genotypes. The detection of EST-SSRs was also an important point regarding sequence function; the sequences containing ESTs will be good candidates for functional studies related to pulp and seed quality as well as to resistance to witches’ broom disease. Moreover, these markers may contribute to develop or saturate both the cupuassu and cacao genetic maps, respectively.

## Supporting Information

S1 Table Characteristics of the 77 EST-SSRs designed in this study. (DOCX)

S2 Table Frequency of different SSR types identified in 1517 ESTs from cupuassu seeds and pulp. (DOCX)

S3 Table Summary of the different SSR data set obtained from cacao and cupuassu and already available in databanks or literature. (DOCX)

## Abbreviations

ATT: titratable acidity
EST: expressed sequence tag
NGS: next generation sequencing
ORF: open reading frame
UTR: untranslated region
SSR: simple sequence repeat
SST: total soluble solids

## References

[1] Clement C, De Cristo-Araújo M, Coppens D’Eeckenbrugge G, Alves Pereira A, Picanço-Rodrigues D. Origin and domestication of native amazonian crops. Diversity. 2010;2(1):72–106. 10.3390/d2010072

[2] Calzavara BBG, Muller CH, Kahwage ONN. Fruticultura Tropical: o cupuaçuzeiro—cultivo, beneficiamento e utilização do fruto. Belém: EMBRAPA-CPATU; 1984 101 p.

[3] Cohen KO, Jackix MNH. Estudo do liquor de cupuaçu. Food Science and Technology (Campinas). 2005;25:182–90.

[4] Lannes SCS, Medeiros ML, Gioielli LA. Physical interactions between cupuassu and cocoa fats. Grasas y Aceites. 2003;54(3): 253–8.

[5] Vasconcelos MNL, da Silva ML, Maia JGS, GO R.. Estudo químico de sementes do cupuaçu. Acta Amazonica. 1975;5:293–5.

[6] Lannes SCdS, Medeiros ML. Processamento de achocolatado de cupuaçu por spray-dryer. Revista Brasileira de Ciências Farmacêuticas. 2003;39:115–23.

[7] Lannes SCdS, Medeiros ML, Amaral RL. Formulação de "chocolate" de cupuaçu e reologia do produto líquido. 2002 2002;38(4):7 Epub 2002-12-01. 10.1590/s1516-93322002000400009

[8] Reisdorff C, Rohsius C, Claret de Souza AdG, Gasparotto L, Lieberei R. Comparative study on the proteolytic activities and storage globulins in seeds of *Theobroma grandiflorum* (Willd ex Spreng) Schum and *Theobroma bicolor* Humb Bonpl, in relation to their potential to generate chocolate-like aroma. Journal of the Science of Food and Agriculture. 2004;84(7):693–700. 10.1002/jsfa.1717

[9] de Oliveira TB, Genovese MI. Chemical composition of cupuassu (*Theobroma grandiflorum*) and cocoa (*Theobroma cacao*) liquors and their effects on streptozotocin-induced diabetic rats. Food Research International. 2013;51(2):929–35. 10.1016/j.foodres.2013.02.019

[10] Yang H, Protiva P, Cui B, Ma C, Baggett S, Hequet V, et al New bioactive polyphenols from *Theobroma grandiflorum* (“Cupuaçu”). Journal of Natural Products. 2003;66(11):1501–4. 10.1021/np034002j 14640528

[11] de Oliveira TB, Rogero MM, Genovese MI. Poliphenolic-rich extracts from cocoa (*Theobroma cacao* L.) and cupuassu (*Theobroma grandiflorum* Willd. Ex Spreng. K. Shum) liquors: A comparison of metabolic effects in high-fat fed rats. PharmaNutrition. 2015;3(2):20–8. 10.1016/j.phanu.2015.01.002

[12] Sayid R. Chocolate could run out in 2020 due to worldwide shortage of cocoa. The Daily Mirror online. 2013 31/12/2013; Sect. World News.

[13] Wexler A. World's sweet tooth heats up cocoa. Growing demand from emerging markets is pushing up prices for key ingredient in chocolate. The Wall Street Journal. 2014 13/02/2014.

[14] Alves RM, Silva CRS, Silva MSC, Silva DCS, Sebbenn AM. Diversidade genética em coleções amazônicas de germoplasma de cupuaçuzeiro [*Theobroma grandiflorum* (Willd. ex Spreng.) Schum.]. Revista Brasileira de Fruticultura. 2013;35:818–28.

[15] Alves RM, Resende MDV, Bandeira BS, Pinheiro TM, Farias DCR. Evolução da vassoura-de-bruxa e avaliação da resistência em progênies de cupuaçuzeiro. Revista Brasileira de Fruticultura. 2009;31:1022–32.

[16] Kuhn DN, Figueira A, Lopes U, Motamayor JC, Meerow AW, Cariaga K, et al Evaluating T*heobroma grandiflorum* for comparative genomic studies with *Theobroma cacao*. Tree Genetics & Genomes. 2010;6(5):783–92. 10.1007/s11295-010-0291-0

[17] Alves RM, Resende MDVd, Bandeira BdS, Pinheiro TM, Farias DCR. Avaliação e seleção de progênies de cupuaçuzeiro (*Theobroma grandiflorum*), em Belém, Pará. Revista Brasileira de Fruticultura. 2010;32:204–12.

[18] Argout X, Fouet O, Wincker P, Gramacho K, Legavre T, Sabau X, et al Towards the understanding of the cocoa transcriptome: Production and analysis of an exhaustive dataset of ESTs of *Theobroma cacao* L. generated from various tissues and under various conditions. BMC Genomics. 2008;9(1):512 10.1186/1471-2164-9-512 18973681 PMC2642826

[19] Argout X, Salse J, Aury J-M, Guiltinan MJ, Droc G, Gouzy J, et al The genome of *Theobroma cacao*. Nat Genet. 2011;43:101–8. 10.1038/ng.736 21186351

[20] Feltus F, Saski C, Mockaitis K, Haiminen N, Parida L, Smith Z, et al Sequencing of a QTL-rich region of the *Theobroma cacao genome* using pooled BACs and the identification of trait specific candidate genes. BMC Genomics. 2011;12(1):379 10.1186/1471-2164-12-379 21794110 PMC3154204

[21] Micheli F, Guiltinan M, Gramacho KP, Wilkinson MJ, Figueira AVdO, Cascardo JCdM, et al Chapter 3—Functional genomics of cacao In: Jean-Claude K, Michel D, editors. Advances in Botanical Research. Volume 55: Academic Press; 2010 p. 119–77.

[22] Aime MC, Phillips-Mora W. The causal agents of witches' broom and frosty pod rot of cacao (chocolate, *Theobroma cacao*) form a new lineage of Marasmiaceae. Mycologia. 2005;97(5):1012–22. 10.3852/mycologia.97.5.1012 16596953

[23] Li Y-C, Korol AB, Fahima T, Beiles A, Nevo E. Microsatellites: genomic distribution, putative functions and mutational mechanisms: a review. Molecular Ecology. 2002;11(12):2453–65. 10.1046/j.1365-294X.2002.01643.x 12453231

[24] Su Haq, Jain R, Sharma M, Kachhwaha S, Kothari SL. Identification and characterization of microsatellites in Expressed Sequence Tags and their cross transferability in different plants. International Journal of Genomics. 2014;2014:863948 10.1155/2014/863948 PMC4217358. 25389527 PMC4217358

[25] Agarwal M, Shrivastava N, Padh H. Advances in molecular marker techniques and their applications in plant sciences. Plant Cell Reports. 2008;27(4):617–31. 10.1007/s00299-008-0507-z 18246355

[26] Varshney R, Graner A, Sorrells M. Genic microsatellite markers in plants: features and applications. Trends in Biotechnology. 2005;23(1):48–55. 10.1016/j.tibtech.2004.11.005 15629858

[27] Nicot N, Chiquet V, Gandon B, Amilhat L, Legeai F, Leroy P, et al Study of simple sequence repeat (SSR) markers from wheat expressed sequence tags (ESTs). Theoretical and Applied Genetics. 2004;109(4):800–5. 10.1007/s00122-004-1685-x 15146317

[28] Taniguchi F, Fukuoka H, Tanaka J. Expressed sequence tags from organ-specific cDNA libraries of tea (*Camellia sinensis*) and polymorphisms and transferability of EST-SSRs across *Camellia* species. Breeding Science. 2012;62(2):186–95. 10.1270/jsbbs.62.186 PMC3405963. 23136530 PMC3405963

[29] Fan L, Zhang MY, Liu QZ, Li LT, Song Y, Wang LF, et al Transferability of Newly Developed Pear SSR Markers to Other Rosaceae Species. Plant Molecular Biology Reporter / Ispmb. 2013;31(6):1271–82. 10.1007/s11105-013-0586-z PMC3881569. PMC3881569 24415844

[30] Lee G-A, Song J, Choi H-R, Chung J-W, Jeon Y-A, Lee J-R, et al Novel microsatellite markers acquired from *Rubus coreanus* Miq. and cross-amplification in other *Rubus* Species. Molecules. 2015;20(4):6432–42. 10.3390/molecules20046432 25867828 PMC6272785

[31] Santos JCS, Barreto MA, Oliveira FA, Vigna BBZ, Souza AP. Microsatellite markers for *Urochloa humidicola* (Poaceae) and their transferability to other *Urochloa* species. BMC Research Notes. 2015;8:83 10.1186/s13104-015-1044-9 PMC4365966. 25889143 PMC4365966

[32] Adal A, Demissie Z, Mahmoud S. Identification, validation and cross-species transferability of novel Lavandula EST-SSRs. Planta. 2015;241(4):987–1004. 10.1007/s00425-014-2226-8 25534945

[33] Sahu J, Das Talukdar A, Devi K, Choudhury MD, Barooah M, Modi MK, et al E-microsatellite markers for *Centella asiatica* (Gotu Kola) genome: validation and cross-transferability in Apiaceae family for plant omics research and development. OMICS: A Journal of Integrative Biology. 2015;19(1):52–65. 10.1089/omi.2014.0113 25562200

[34] Shrivastava D, Verma P, Bhatia S. Expanding the repertoire of microsatellite markers for polymorphism studies in Indian accessions of mung bean (*Vigna radiata* L. Wilczek). Molecular Biology Reports. 2014;41(9):5669–80. 10.1007/s11033-014-3436-7 24913033

[35] Raveendar S, Lee G-A, Jeon Y-A, Lee Y, Lee J-R, Cho G-T, et al Cross-amplification of *Vicia sativa* subsp. sativa microsatellites across 22 Other *Vicia* Species. Molecules. 2015;20(1):1543–50. 10.3390/molecules20011543 25608853 PMC6272350

[36] Bhawna, Abdin MZ, Arya L, Verma M. Transferability of cucumber microsatellite markers used for phylogenetic analysis and population structure study in Bottle Gourd (*Lagenaria siceraria* (Mol.) Standl.). Applied Biochemistry and Biotechnology. 2015;175(4):2206–23. 10.1007/s12010-014-1395-z 25471016

[37] Faleiro FG, Queiroz VT, Lopes UV, Guimarães CT, Pires JL, Yamada MM, et al Mapping QTLs for witches' broom (*Crinipellis perniciosa*) resistance in cacao (*Theobroma cacao* L.). Euphytica. 2006;149(1–2):227–35. 10.1007/s10681-005-9070-7

[38] Alves RM, Garcia AAF, Cruz ED, Figueira A. Seleção de descritores botânico-agronômicos para caracterização de germoplasma de cupuaçuzeiro. Pesquisa Agropecuaria Brasileira. 2003;38:807–18.

[39] Papanicolaou A, Stierli R, ffrench-Constant R, Heckel D. Next generation transcriptomes for next generation genomes using est2assembly. BMC Bioinformatics. 2009;10(1):447 10.1186/1471-2105-10-447 20034392 PMC3087352

[40] Chevreux B, Pfisterer T, Drescher B, Driesel AJ, Müller WEG, Wetter T, et al Using the miraEST assembler for reliable and automated mRNA transcript assembly and SNP detection in sequenced ESTs. Genome Research. 2004;14(6):1147–59. 10.1101/gr.1917404 15140833 PMC419793

[41] Thiel T, Michalek W, Varshney R, Graner A. Exploiting EST databases for the development and characterization of gene-derived SSR-markers in barley (*Hordeum vulgare* L.). Theoretical and Applied Genetics. 2003;106(3):411–22. 10.1007/s00122-002-1031-0 12589540

[42] Altschul S, Gish W, Miller W, Myers E, Lipman D. Basic local alignment search tool. Journal of Molecular Biology. 1990;215(3):403–10. 2231712 10.1016/S0022-2836(05)80360-2

[43] Consortium GO. The Gene Ontology (GO) database and informatics resource. Nucleic Acids Research. 2004;32(suppl 1):D258–D61. 10.1093/nar/gkh036 14681407 PMC308770

[44] Gesteira AS, Micheli F, Carels N, Da Silva A, Gramacho K, Schuster I, et al Comparative analysis of expressed genes from cacao meristems infected by *Moniliophthora perniciosa*. Ann Bot. 2007;100(1):129–40. Epub 2007/06/15. mcm092 [pii] 10.1093/aob/mcm092 17557832 PMC2735303

[45] Doyle JJ, Doyle JL. Isolation of plant DNA from fresh tissue1990.

[46] Lewis PO, Zaykin D. Genetic Data Analysis. 1.0. ed2001.

[47] Anderson JA, Churchill GA, Autrique JE, Tanksley SD, Sorrells ME. Optimizing parental selection for genetic linkage maps. Genome. 1993;36(1):181–6. 10.1139/g93-024 . 18469981

[48] Van Oosterhout C, Hutchinson WF, Wills DPM, Shipley P. micro-checker: software for identifying and correcting genotyping errors in microsatellite data. Molecular Ecology Notes. 2004;4(3):535–8. 10.1111/j.1471-8286.2004.00684.x

[49] Belkhir K, Borsa P, Chikhi L, Raufaste N, Bonhomme F. GENETIX 4.05, logiciel sous Windows TM pour la génétique des populations. Montpellier (France): Laboratoire Génome, Populations, Interactions, CNRS UMR 5000, Université de Montpellier II; 1996–2004

[50] SAS I. SAS/STAT user’s guide. Release 6.03 ed. SAS Institute Inc., Cary, NC 10281988.

[51] Leal G, Albuquerque P, Figueira A. Genes differentially expressed in *Theobroma caca*o associated with resistance to witches' broom disease caused by *Crinipellis perniciosa*. Molecular Plant Pathology. 2007;8(3):279–92. 10.1111/j.1364-3703.2007.00393.x 20507499

[52] Verica J, Maximova S, Strem M, Carlson J, Bailey B, Guiltinan M. Isolation of ESTs from cacao (*Theobroma cacao* L.) leaves treated with inducers of the defense response. Plant cell reports. 2004;23(6):404–13. 10.1007/s00299-004-0852-5 15340758

[53] Jones P, Allaway D, Gilmour D, Harris C, Rankin D, Retzel E, et al Gene discovery and microarray analysis of cacao (*Theobroma cacao* L.) varieties. Planta. 2002;216(2):255–64. 10.1007/s00425-002-0882-6 12447539

[54] Naganeeswaran SA, Subbian EA, Ramaswamy M. Analysis of expressed sequence tags (ESTs) from cocoa (*Theobroma cacao* L.) upon infection with *Phytophthora megakarya*. Bioinformation. 2012;8(2):65–9. PMC3282258. 22359437 10.6026/97320630008065 PMC3282258

[55] Asadi A, Rashidi Monfared S. Characterization of EST-SSR markers in durum wheat EST library and functional analysis of SSR-containing EST fragments. Molecular Genetics and Genomics. 2014;289(4):625–40. 10.1007/s00438-014-0839-z 24652471

[56] Kumari K, Muthamilarasan M, Misra G, Gupta S, Subramanian A, Parida SK, et al Development of eSSR-Markers in Setaria italica and their applicability in studying genetic diversity, cross-transferability and comparative mapping in Millet and Non-Millet Species. PLoS ONE. 2013;8(6):e67742 10.1371/journal.pone.0067742 PMC3689721. 23805325 PMC3689721

[57] Kantety R, La Rota M, Matthews D, Sorrells M. Data mining for simple sequence repeats in expressed sequence tags from barley, maize, rice, sorghum and wheat. Plant Molecular Biology. 2002;48(5–6):501–10. 10.1023/a:1014875206165 11999831

[58] Lima LS, Gramacho KP, Pires JL, Clement D, Lopes UV, Carels N, et al Development, characterization, validation, and mapping of SSRs derived from *Theobroma cacao* L.–*Moniliophthora perniciosa* interaction ESTs. Tree Genetics & Genomes. 2010;6(5):663–76. 10.1007/s11295-010-0282-1

[59] Bosamia TC, Mishra GP, Thankappan R, Dobaria JR. Novel and stress relevant EST derived SSR markers developed and validated in Peanut. PLoS ONE. 2015;10(6):e0129127 10.1371/journal.pone.0129127 26046991 PMC4457858

[60] Metzgar D, Bytof J, Wills C. Selection against frameshift mutations limits microsatellite expansion in coding DNA. Genome Research. 2000;10(1):72–80. PMC310501. 10645952 PMC310501

[61] Aggarwal R, Hendre P, Varshney R, Bhat P, Krishnakumar V, Singh L. Identification, characterization and utilization of EST-derived genic microsatellite markers for genome analyses of coffee and related species. Theoretical and Applied Genetics. 2007;114(2):359–72. 10.1007/s00122-006-0440-x 17115127

[62] Santos R, Clement D, Lemos L, Legravre T, Lanaud C, Schnell R, et al Identification, characterization and mapping of EST-derived SSRs from the cacao–*Ceratocystis cacaofunesta* interaction. Tree Genetics & Genomes. 2013;9(1):117–27. 10.1007/s11295-012-0539-y

[63] Gonçalves MVVA, da Silva JPL, Mathias SP, Rosenthal A, Calado VMA. Caracterização fisico-chimica e reologica da polpa de cupuaçu congelada (*Theobroma grandiflorum* Schum). Perspectivas Online Exatas e Engenharia. 2013; 3(7).

[64] Santos GM, Maia GA, Sousa PHM, Figueiredo RW, Costa JMC, Fonseca AVV. Atividade antioxidante e correlações com componentes bioativos de produtos comerciais de cupuaçu. Ciência Rural. 2010;40:1636–42.

[65] Regulamento técnico geral para fixação dos padrões de identidade e qualidade para polpa de fruta conforme consta do Anexo I desta Instrução Normativa., Instrução normativa n°01 (2000).

[66] Costa MC, Maia GA, Souza Filho MdSM, Figueiredo RWd, Nassu RT, Monteiro JCS. Conservação de polpa de cupuaçu [*Theobroma grandiflorum* (Willd. Ex Spreng.) Schum] por métodos combinados. Revista Brasileira de Fruticultura. 2003;25:213–5.

[67] Bueno SM, Lopes MRV, Graciano RAS, Fernandes ECB, Garcia-Cruz CH. Quality evaluation of frozen fruit pulp. Rev Inst Adolfo Lutz. 2002;62(2):121–6.

[68] Alves RM, Sebbenn AM, Artero AS, Figueira A. Microsatellite loci transferability from *Theobroma cacao* to *Theobroma grandiflorum*. Molecular Ecology Notes. 2006;6(4):1219–21. 10.1111/j.1471-8286.2006.01496.x

[69] Lanaud C, Risterucci AM, Pieretti I, Falque M, Bouet A, Lagoda PJL. Isolation and characterization of microsatellites in *Theobroma cacao* L. Molecular Ecology. 1999;8(12):2141–3. 10.1046/j.1365-294x.1999.00802.x 10632866

[70] Wang L, Chen H, Bai P, Wu J, Wang S, Blair M, et al The transferability and polymorphism of mung bean SSR markers in rice bean germplasm. Molecular Breeding. 2015;35(2):1–10. 10.1007/s11032-015-0280-y

[71] Ferrão L, Caixeta E, Pena G, Zambolim E, Cruz C, Zambolim L, et al New EST–SSR markers of *Coffea arabica*: transferability and application to studies of molecular characterization and genetic mapping. Molecular Breeding. 2015;35(1):1–5. 10.1007/s11032-015-0247-z

[72] Backiyarani S, Uma S, Varatharj P, Saraswathi MS. Mining of EST-SSR markers of Musa and their transferability studies among the members of order the Zingiberales. Applied Biochemistry and Biotechnology. 2013;169(1):228–38. 10.1007/s12010-012-9975-2 23179283

[73] Castillo A, Budak H, Varshney RK, Dorado G, Graner A, Hernandez P. Transferability and polymorphism of barley EST-SSR markers used for phylogenetic analysis in *Hordeum chilense*. BMC Plant Biology. 2008;8:97- 10.1186/1471-2229-8-97 PMC2569940. 18822176 PMC2569940

[74] Gong L, Deng Z. EST-SSR markers for gerbera (*Gerbera hybrida*). Molecular Breeding. 2010;26(1):125–32. 10.1007/s11032-009-9380-x

