# Supplementary material for: First Microsatellite Markers Developed from Cupuassu ESTs: Application in Diversity Analysis and Cross-Species Transferability to Cacao
Source: PLoS One. 2016 Mar 7;11(3):e0151074. doi: 10.1371/journal.pone.0151074 (PMC4780773; doi:10.1371/journal.pone.0151074)
Supplement: S1 Table — (DOCX) [file pone.0151074.s001.docx]

**S1 Table. Characteristics of the 77 EST-SSRs designed in this study.**

| Marker name | Primers forward (F) and reverse (R) | Repeat motif  (nº of repeat) | Putative gene function | Organism | Origin library |
| --- | --- | --- | --- | --- | --- |
| c45A | F: AGAAGGGTTAGCCCCAGTGT  R: TCCCGAAATCGAAATTCAAG | A(14) | Predicted protein | *Populus trichocarpa* | Pulp/Seed |
| c5993 | F: TCAAACGCTTAAAAGATAAAGAAGA  R: TGGGGTTTTGGTTTTCTGAC | A(10) | TPA_inf: aquaporin SIP1;2 | *Gossypium hirsutum* | Pulp |
| c2723 | F: AACTCTTCAGTCTGGCAAACAT  R: AAAGTAGCCTTTCCAGAGGAAC | (CAT)6 | AML1 | *Citrus unshiu* | Seed |
| c4949 | F: AGGTGATCAGAAAATGGGAAC  R: ACTGAGTCCACAACTGATCCTT | (T)13 | Repressor of gene silencing 1 | *Gossypium hirsutum* | Pulp |
| c8207 | F: GAGACGATATTGGGGAGTACAA  R: CTTCCCCTTTTGTCTCTACCTT | (TC)6 | AML1 | *Citrus unshiu* | Pulp/Seed |
| c2987 | F: GCTGCTACTTCTGCTTATGTTTG  R: TAGCTAGCCTTCTGGAAAATTG | (TA)11 | Glutathione S-transferase GST 18 | *Populus alba x Populus tremula var. glandulosa* | Pulp/Seed |
| c5718 | F: TAATGGTTCCTGCAAACTCTCT  R: CAGGCTCTTACTCTTCATCGTT | (CTC)5 | Disease resistance protein RPM1 | *Ricinus communis* | Pulp |
| c4546 | F: TGCTTGGGAGAATAGTTTTCAG  R: TACATTGCATTTACCACCTGAG | (TC)6 | mgatp-energized glutathione s-conjugate pump | *Ricinus communis* | Pulp |
| c1773 | F: TATGCTTTGTGTCTGGCTATTG  R: CCTCCACTTCTCAAACAGACTT | (A)10 | Aquaporin 2 | *Samanea saman* | Pulp/Seed |
| c692A | F: ATCAAGATCCCCAACATACAAC  R: AAACAAGGCAAGGAGGTAATGT | (CTG)7 | Lipid transfer protein 4 precursor | *Gossypium hirsutum* | Pulp/Seed |
| c692B | F: ATCAAGATCCCCAACATACAAC  R: TCCCCTTTCTTTCTCCTTTT | (TTGTTTT)2 | Lipid transfer protein 4 precursor | *Gossypium hirsutum* | Pulp/Seed |
| c692C | F: ATCAAGATCCCCAACATACAAC  R: TCCCCTTTCTTTCTCCTTTT | A(10) | Lipid transfer protein 4 precursor | *Gossypium hirsutum* | Pulp/Seed |
| c3464 | F: TAAAGAGGCCACATCAGAGTTT  R: GCAGAAAAATATTGGCTACCAG | (TCA)5 | JHL03K20.4 | *Jatropha curcas* | Pulp/Seed |
| c806 | F: GAATCACATGAAACTCCTCCAT  R: AAAGATTCTTGGACCTGGACTT | (T)11 | Triacylglycerol lipase | *Ricinus communis* | Pulp/Seed |
| c366 | F: TCACTATCAAACTCGTGCAAAC  R: GAGACATGTCACTGCATTTTGT | (T)10 | Choline/ethanolamine kinase | *Ricinus communis* | Pulp/Seed |
| c4063 | F: GACGAGTGGGAGGAACAGTAT  R: AAGTCCGCAATCCTACTATCAA | (GAG)6 | Phospholipid-transporting atpase | *Ricinus communis* | Pulp |
| c1143 | F: CCCTCCTTTACTTCTTGACCTT  R: CCCTCATGATCAAGCTACTTTT | (ATT)5 | Transcription regulator | *Ricinus communis* | Pulp/Seed |
| c513 | F: AATTTCAAAGGGCTGGAGAC  R: TGGAGGGTTTAGGAATGTTATG | (A)10 | Transcription regulator | *Ricinus communis* | Pulp/Seed |
| c45 | F: ATAACCCACTTCCCTTCAACTT  R: CGCCTTCTCTTCGATAAATTC | (AATT)4 | Translationally controlled tumor protein | *Elaeis guineensis* | Pulp/Seed |
| c573 | F: AACCAAACTCTGGTCTTTTTCC  R: GAATTGGGTGAATCTGAAGTGT | (TGC)5 | Translationally controlled tumor protein | *Elaeis guineensis* | Pulp/Seed |
| c70 | F: GAGAGAGACTTCAACAAAGGAGA  R: TCAAGTGGTCATCTACATACGC | (AGA)6 | Ethylene-responsive element-binding factor | *Gossypium hirsutum* | Pulp/Seed |
| c3202 | F: GAGCAGCACTAGGACAAGAGAT  R: ATGGTAAAGAGGAAGAGGAACC | (GAA)6 | CBL-interacting serine/threonine-protein kinase | *Ricinus communis* | Pulp |
| c3202B | F: AGAGATGAGGAGAACAACAGGA  R: ATGGTAAAGAGGAAGAGGAACC | (GAA)6 | CBL-interacting serine/threonine-protein kinase | *Ricinus communis* | Pulp |
| c83 | F: CTAAGTTGCCTGAAGAAAGGAA  R: TCCCAAGCTTTAAACAGAGCTA | (T)10 | Serine-threonine protein kinase | *Ricinus communis* | Pulp/Seed |
| c4380 | F: GCTCTTCAATTGCAACTTATCC  R: CATATTTTCAACACCTCGCTCT | (A)10 | Wall-associated kinase | *Ricinus communis* | Pulp |
| c4943 | F: ATCATCAGCACAGCTTCAAGTA  R: TAGAATATCGGAGCGAGTTCAT | (GAT)5 | Kinase | *Ricinus communis* | Pulp/Seed |
| c664 | F: CTACTAGCTTCCTTGCTTGCAT  R: CCATCTTGTAAGTCTCGAGGTC | (T)10 | Ribosomal protein subunit 10 | *Solanum tuberosum* | Pulp/Seed |
| c109 | F: AAAGGACCCAAGGTAATCAAGT  R: ATTACAAGGAAAAAGGGAGACC | (A)10 | Cystathionine gamma-synthase isoform 1 | *Solanum tuberosum* | Pulp/Seed |
| c733 | F: TTGCCAGGTCTACTCAAAAGTT  R: GGTGGAAGATATCAACCTGAAA | (CAA)2(CAA)2 | Ubiquitin-activating enzyme E1 | *Ricinus communis* | Pulp |
| c784 | F: TTTTCAATATGACCCCTACTGC  R: AGAAAGATCTGCGAGGTACTTG | (T)10 | E3 ubiquitin protein ligase upl2 | *Ricinus communis* | Pulp/Seed |
| c122 | F: GTACAATGCATCCAACAATGAG  R: ACCATAGTGCTGGAGATACCAC | (CAG)7 | 40S ribosomal protein S8 | *Elaeis guineensis* | Pulp/Seed |
| c1410 | F: ACCTCGCGTTCATGATATCTAC  R: GACGGACACGACTTCTTCTAAC | (G)10 | Ribosomal protein L2 | *Vitis vinifera* | Pulp/Seed |
| c180 | F: AATGTAACCGTTCTTACGGATG  R: ATTTTCCTCTTCGAAGCCTTAC | (GA)8 | Eukaryotic translation initiation factor 5A isoform I | *Hevea brasiliensis* | Pulp/Seed |
| c3128 | F: GTAGGGCAGGAGGAGTTCTATT  R: CTTCTCGGGGTAGTAATGACAG | (T)10 | Ribosomal protein S8 | *Talipariti macrophyllum* | Pulp |
| c193A | F: GTCACGGAATGGTTAGAGTGAT  R: GGAGCTATTACAAGTGCACACA | (TTG)5 | Similar to DNA-binding protein | *Vitis vinifera* | Pulp/Seed |
| c193B | F: TCAAACTCATCTCCTCCAGATT  R: CACAAAGAAGAAGGTTGAAAGG | (GAT)4 | Similar to DNA-binding protein | *Vitis vinifera* | Pulp/Seed |
| c203A | F: CCAAGAGAGAAACAAGGAGAGA  R: ATGCTCAAGGATACGATTATGG | (TTG)5 | Nuclear acid binding protein | *Ricinus communis* | Pulp/Seed |
| c203B | F: TAATACTGCAAATCTCCGATCC  R: AACAGTACCAATACCAGCAACC | (TTGACCCGC)2 | Nuclear acid binding protein | *Ricinus communis* | Pulp/Seed |
| c370A | F: TTTCCCTGTTTCTTCTGTGTCT  R: ATCCTCAAGGACTCTCTCATCA | (T)11 | DNA binding protein | *Ricinus communis* | Pulp/Seed |
| c370B | F: TTTCGCTGTATCAACTGTATCG  R: GTATCAAAATTCGGTGACCACT | (TGC)4 | DNA binding protein | *Ricinus communis* | Pulp/Seed |
| c1251 | F: AAGGTACCACTGAACCAAACAG  R: CAGAGTCATGGAGCCTACTACC | (TGA)6 | Topoisomerase I | *Camptotheca acuminata* | Pulp/Seed |
| c1974 | F: GCTTCTGTTTCTGTTCCTGTTG  R: AACCGGAGGTTATTTGTTTCTC | (A)10 | DNA binding/zinc ion binding protein | *Gossypium hirsutum* | Pulp/Seed |
| c2674 | F: CTTGCACATGAAGTTGATTCTG  R: TCTAATGCCATACCAAAAGGAC | (TGT)6 | DNA excision repair protein | *Citrus unshiu* | Pulp |
| c3481 | F: GGAGGATGTTAGTGCGATAGAG  R: ACCTATGCTCTTCTTCTGTCCA | (GCT)7 | DNA binding protein | *Ricinus communis* | Pulp/Seed |
| c239 | F: CACGAATTTTCTTCAACTCCTC  R: ACATGGATGATAGTGATGCAGA | (AGG)6 | Acireductone dioxygenase | *Ricinus communis* | Pulp/Seed |
| c345 | F: CTATGAGCAAACGGATCTTTTC  R: TTCTTGGGTTAATACCCTTGTG | (GCT)8 | Transcription factor | *Bruguiera gymnorhiza* | Pulp/Seed |
| c339 | F: AAGGCATTGAGTTCTTCAGTGT  R: GGGGAAGATCCCATATCTAAAG | (AAAT)2 | Transcription factor | *Ricinus communis* | Pulp/Seed |
| c430 | F: AAGCCTAGGGGATACAAAAGAG  R: CAGAAAATCCAGCTTTTAGTGG | (TGC)7 | AP2/ERF domain-containing transcription factor | *Populus trichocarpa* | Pulp/Seed |
| c651 | F: GTTCAATCCTCTGTTTCTTTGG  R: AAGAAGAAGAAGGTGGGAAAAG | (CTT)7 | AP2/ERF domain-containing transcription factor | *Populus trichocarpa* | Pulp/Seed |
| c513 | F: GCTTGTACACGTGAGAAGATGA  R: TTACTAACGGAGGGTATGATGG | (A)10 | r2r3-myb transcription factor | *Ricinus communis* | Pulp/Seed |
| c2763F | F: CATATAAGGAGGCAGTGAGAGG  R: TTATGGGAAGTTTGACCATAGG | (CCA)6 | Transcription factor hy5 | *Ricinus communis* | Seed |
| c2723 | F: GTCCATCAAATGGTTCGAGTAT  R: ATGTTTGCCAGACTGAAGAGTT | (CAT)6 | AML1 | *Citrus unshiu* | Seed |
| c3359 | F: CCTAAAGTTTTGTCCATGAAGG  R: CTTCAAAAGGACATGTTTCTCC | (A)11 | Transcription factor | *Ricinus communis* | Pulp/Seed |
| c4949 | F: CTGACCATCTTTCTAGGTGAGC  R: GATTTGAGAGGAAAATGAGCAG | (T)13 | Tepressor of gene silencing 1 | *Gossypium hirsutum* | Pulp |
| c8207 | F: AACTCTCGGATTCTTTCTTTCC  R: CTTCCCCTTTTGTCTCTACCTT | (TC)6 | Transcription initiation factor IIB | *Glycine max* | Pulp/Seed |
| c2987 | F: GTCCCCAAGAACTTTCTCTTCT  R: GGCATTGACCTATCATTCTTTC | (TA)11 | Glutathione S-transferase GST 18 | *Populus alba x Populus tremula var. glandulosa* | Pulp/Seed |
| c5718 | F: CGGAAAAACGATGAAGAGTAAG  R: ATGCCCTGATCAGTTTCTTCTA | (CTC)5 | Disease resistance protein RPM1 | *Ricinus communis* | Pulp |
| c246 | F: ATCTATCCCCCATAGGAAACAC  R: TTTTATCTGAAGAGGGTTGGAG | (A)10 | ATPase subunit 8 | *Ricinus communis* | Pulp/Seed |
| c297 | F: ACCATGAAAAAGGCCTACTCTT  R: CCCTCTTCCGTTAGAGAGATTT | (T)10 | ATPase subunit 4 | *Cucurbita pepo* | Pulp/Seed |
| c295 | F: GTCATATACAACCCCGACGTAT  R: ATCTCTCACGAGGCCATAAAT | (T)10 | NADH dehydrogenase subunit 4L | *Nicotiana tabacum* | Pulp/Seed |
| c351 | F: AGAGAGGGAAATTACAGCCTTC  R: ACAAAGGTATGAGAGCAGCATT | (T)10 | Serine/threonine protein phosphatase | *Ricinus communis* | Pulp/Seed |
| c431B | F: GGAAATGTTTGGAACAGAGAAC  R: CAATATCTTGAAAGGCGAAGAG | (A)10(TA)8(TA)6(TA10) | Ribosomal protein S14 | *Nicotiana tabacum* | Pulp/Seed |
| c431A | F: GAGAGTGCTGGAGAGAAGAGAA  R: GTTCTGGGGCCCTACTTACTAT | (AAGA)3 | Ribosomal protein S14 | *Nicotiana tabacum* | Pulp/Seed |
| c399A | F: TCTTATTCTGCCATATGCACTG  R: TGCTGAGGATGGATATACTGTG | (A)10 | Patellin-3 | *Ricinus communis* | Pulp/Seed |
| c399B | F: CTTCTTCCTTGACTTCAACAGG  R: TATTCAAGAGGCCCTTAACAAG | (TTCTTCTTT)2 | Patellin-3 | *Ricinus communis* | Pulp/Seed |
| c432 | F: AATCGGTCTTCAAGTTGTCACT  R: ATATTCTCTCCCCCAGTTTCAT | (CAT)5 | Importin beta-1 | *Ricinus communis* | Pulp/Seed |
| c559 | F: TGTACCATAGCTCAGGACATCA  R: AAGCTGCCTTAACATCAGCTAC | (A)10 | Pyruvate kinase | *Glycine max* | Pulp/Seed |
| c663A | F: GCTCCTCTCGATCTTATTCGTA  R: CCCACGAAGATCTCTACGTACT | (CAGAC)3 | NADH dehydrogenase subunit 4 | *Arabidopsis thaliana* | Pulp/Seed |
| c663B | F: TATGGTTTTGATATCCGGTAGG  R: AAACACCCATCCAAACAAGTAG | (T)10 | NADH dehydrogenase subunit 4 | *Arabidopsis thaliana* | Pulp/Seed |
| c8097 | F: GTGATGGGAAACAGACTAGGAG  R: CGGAAGTCTGAAACCATTAGAG | (GCA)5 | dtdp-glucose 4-6-dehydratase | *Ricinus communis* | Pulp/Seed |
| c618 | F: CATAAAATCAGCGGGTAGAGAG  R: GTGGAAAGACGAAAGTCTTCAG | (CT)6 | NADP-dependent malic protein | *Ricinus communis* | Pulp/Seed |
| c1149 | F: AAAAATGTCATTCCCACCAG  R: CTCGGGTATGGATCTTCTTGTA | (AGA)6 | Glucan endo-1,3-beta-glucosidase precursor | *Ricinus communis* | Pulp/Seed |
| c2564 | F: TTGCATAAAGTCATCACCAGAG  R: ACAATCCTCGAAAGGCATACTA | (AAT)5 | Arginine/serine rich splicing factor sf4/14 | *Ricinus communis* | Pulp |
| c1295 | F: TACATTCTCAAACGTCATCTCG  R: CTGCTGTTGTTGGGATATTTG | (ATG)5 | Transcription elongation factor s-II | *Ricinus communis* | Pulp/Seed |
| c7101 | F: ATGGACATAGGTTCTGCTTTTG  R: GTATGAGAAATGGGATGCTGAT | (CTT)5 | Helicase | *Ricinus communis* | Seed |
| c7219 | F: TTAGCAGTTGTCAAAGTCGAAC  R: GGATTGTAAGAATTGGGGTTCT | (AAG)5 | Frigida | *Populus tremula* | Seed |
| c7297 | F: CGGTCTTTCAGTCTTTTCAATC  R: GACCCATTCAGCTTGAACTTAG | (T)10 | MIM (hypersensitive to MMS, irradiation and MMC) | *Vitis vinifera* | Seed |
