# Supplementary material for: First Microsatellite Markers Developed from Cupuassu ESTs: Application in Diversity Analysis and Cross-Species Transferability to Cacao
Source: PLoS One. 2016 Mar 7;11(3):e0151074. doi: 10.1371/journal.pone.0151074 (PMC4780773; doi:10.1371/journal.pone.0151074)
Supplement: S2 Table — (DOCX) [file pone.0151074.s002.docx]

**S2 Table. Frequency of different SSR types identified in 1517 ESTs from cupuassu seeds and pulp.**

| Repeats | 2 | 3 | 4 | 5 | 6 | 7 | 8 | 9 | 10 | 11 | 12 | 13 | 14 | 15 | 16 | 17 | 18 | 19 | total | Frequency (%) |
| --- | --- | --- | --- | --- | --- | --- | --- | --- | --- | --- | --- | --- | --- | --- | --- | --- | --- | --- | --- | --- |
| A | - | - | - | - | - | - | - | - | 73 | 7 | 2 | 1 | 1 | 1 | - | 1 | - | - | 86 | 4.53 |
| T | - | - | - | - | - | - | - | - | 59 | 8 | 4 | 3 | 4 | - | 1 | 2 | - | - | 81 | 4.27 |
| C | - | - | - | - | - | - | - | - | 1 | 1 | - | - | - | - | - | - | - | - | 2 | 0.11 |
| G | - | - | - | - | - | - | - | - | 2 | - | - | - | - | - | - | - | - | - | 2 | 0.11 |
| AT | - | - | - | - | 3 | 5 | 3 | 1 | 6 | 1 | 8 | 3 | - | - | - | - | - | - | 30 | 1.58 |
| AG | - | - | - | - | 8 | 4 | 4 | 2 | 2 | 2 | 1 | 2 | 1 | 1 | 1 | - | - | - | 28 | 1.47 |
| TC | - | - | - | - | 10 | 4 | 4 | 2 | 2 | 3 | - | 1 | - | - | - | - | - | 1 | 27 | 1.42 |
| GA | - | - | - | - | 6 | 4 | 2 | 2 | - | - | 2 | 2 | 1 | - | - | - | - | - | 19 | 1.00 |
| TA | - | - | - | - | 5 | 3 | 3 | 2 | 2 | 2 | - | - | - | - | 1 | - | - | - | 18 | 0.95 |
| CT | - | - | - | - | 7 | - | 2 | - | 1 | - | - | - | - | 1 | - | - | - | - | 11 | 0.58 |
| TG | - | - | - | - | 3 | 1 | 1 | - | - | - | - | - | - | - | - | - | - | - | 5 | 0.26 |
| AC | - | - | - | - | 4 | - | - | - | - | - | - | - | - | - | - | - | - | - | 4 | 0.21 |
| GAA | - | - | 14 | 6 | 1 | 4 |  | - | - | - | - | - | - | - | - | - | - | - | 25 | 1.32 |
| AAG | - | - | 16 | 3 | 2 | 2 | 1 | - | - | - | - | - | - | - | - | - | - | - | 24 | 1.26 |
| CTT | - | - | 13 | 6 | - | 3 | 1 | - | - | - | - | - | - | - | - | - | - | - | 23 | 1.21 |
| TCT | - | - | 14 | 4 | 2 | 1 | - | - | 1 | - | - | - | - | - | - | - | - | - | 22 | 1.16 |
| TTC | - | - | 17 | 2 | 1 | 1 | 1 | - | - | - | - | - | - | - | - | - | - | - | 22 | 1.16 |
| GCT | - | - | 14 | 4 | - | 1 | 1 | - | - | - | - | - | - | - | - | - | - | - | 20 | 1.05 |
| TGC | - | - | 11 | 6 | - | 3 | - | - | - | - | - | - | - | - | - | - | - | - | 20 | 1.05 |
| TCA | - | - | 15 | 3 | - | - | 1 | - | - | - | - | - | - | - | - | - | - | - | 19 | 1.00 |
| AGA | - | - | 9 | 4 | 4 | - | - | - | - | - | - | - | - | - | - | - | - | - | 17 | 0.90 |
| AGC | - | - | 14 | 2 | - | 1 |  | - | - | - | - | - | - | - | - | - | - | - | 17 | 0.90 |
| CCA | - | - | 11 | 3 | 1 | 1 | - | - | - | - | - | - | - | - | - | - | - | - | 16 | 0.84 |
| GCA | - | - | 11 | 5 | - | - | - | - | - | - | - | - | - | - | - | - | - | - | 16 | 0.84 |
| TGA | - | - | 11 | 2 | 2 | 1 | - | - | - | - | - | - | - | - | - | - | - | - | 16 | 0.84 |
| CAT | - | - | 5 | 8 | 2 | - | - | - | - | - | - | - | - | - | - | - | - | - | 15 | 0.79 |
| ATG | - | - | 9 | 2 | - | 1 | - | - | - | - | - | - | - | - | - | - | - | - | 12 | 0.63 |
| CTC | - | - | 9 | 1 | 1 | - | 1 | - | - | - | - | - | - | - | - | - | - | - | 12 | 0.63 |
| CTG | - | - | 7 | 2 | - | 1 | 1 | - | - | - | - | - | - | - | - | - | - | - | 11 | 0.58 |
| GAG | - | - | 7 | 2 | 2 | - | - | - | - | - | - | - | - | - | - | - | - | - | 11 | 0.58 |
| TTG | - | - | 5 | 4 | 1 | - | - | 1 | - | - | - | - | - | - | - | - | - | - | 11 | 0.58 |
| ATC | - | - | 8 | 2 | - | - | - | - | - | - | - | - | - | - | - | - | - | - | 10 | 0.53 |
| AAT | - | - | 3 | 4 | 2 | - | - | - | - | - | - | - | - | - | - | - | - | - | 9 | 0.47 |
| TGG | - | - | 9 | - | - | - | - | - | - | - | - | - | - | - | - | - | - | - | 9 | 0.47 |
| ATT | - | - | 6 | 1 | - | 1 | - | - | - | - | - | - | - | - | - | - | - | - | 8 | 0.42 |
| TCC | - | - | 5 | 2 | - | - | 1 | - | - | - | - | - | - | - | - | - | - | - | 8 | 0.42 |
| CAG | - | - | 3 | 2 | 1 | 1 | - | - | - | - | - | - | - | - | - | - | - | - | 7 | 0.37 |
| TTA | - | - | 4 | 2 | 1 | - | - | - | - | - | - | - | - | - | - | - | - | - | 7 | 0.37 |
| AAC | - | - | 4 | 1 | - | 1 | - | - | - | - | - | - | - | - | - | - | - | - | 6 | 0.32 |
| ACC | - | - | 5 | 1 | - | - | - | - | - | - | - | - | - | - | - | - | - | - | 6 | 0.32 |
| CAC | - | - | 5 | 1 | - | - | - | - | - | - | - | - | - | - | - | - | - | - | 6 | 0.32 |
| GAT | - | - | 3 | 3 | - | - | - | - | - | - | - | - | - | - | - | - | - | - | 6 | 0.32 |
| TAT | - | - | 3 | 1 | - | - | 2 | - | - | - | - | - | - | - | - | - | - | - | 6 | 0.32 |
| ATA | - | - | 1 | 3 | 1 | - | - | - | - | - | - | - | - | - | - | - | - | - | 5 | 0.26 |
| GGT | - | - | 2 | 2 | 1 | - | - | - | - | - | - | - | - | - | - | - | - | - | 5 | 0.26 |
| TGT | - | - | 2 | 1 | 1 | - | 1 | - | - | - | - | - | - | - | - | - | - | - | 5 | 0.26 |
| ACA | - | - | 4 | - | - | - | - | - | - | - | - | - | - | - | - | - | - | - | 4 | 0.21 |
| AGG | - | - | 3 | - | 1 | - | - | - | - | - | - | - | - | - | - | - | - | - | 4 | 0.21 |
| CAA | - | - | 2 | 1 | - | - | 1 | - | - | - | - | - | - | - | - | - | - | - | 4 | 0.21 |
| GCG | - | - | 3 | 1 | - | - | - | - | - | - | - | - | - | - | - | - | - | - | 4 | 0.21 |
| CCT | - | - | 3 | - | - | - | - | - | - | - | - | - | - | - | - | - | - | - | 3 | 0.16 |
| GGC | - | - | 2 | 1 | - | - | - | - | - | - | - | - | - | - | - | - | - | - | 3 | 0.16 |
| GTT | - | - | 3 | - | - | - | - | - | - | - | - | - | - | - | - | - | - | - | 3 | 0.16 |
| TAC | - | - | 2 | - | 1 | - | - | - | - | - | - | - | - | - | - | - | - | - | 3 | 0.16 |
| ACG | - | - | 1 | 1 | - | - | - | - | - | - | - | - | - | - | - | - | - | - | 2 | 0.11 |
| CCG | - | - | - | - | 2 | - | - | - | - | - | - | - | - | - | - | - | - | - | 2 | 0.11 |
| CGC | - | - | 1 | - | - | - | 1 | - | - | - | - | - | - | - | - | - | - | - | 2 | 0.11 |
| GCC | - | - | 2 | - | - | - | - | - | - | - | - | - | - | - | - | - | - | - | 2 | 0.11 |
| GGA | - | - | 2 | - | - | - | - | - | - | - | - | - | - | - | - | - | - | - | 2 | 0.11 |
| GTC | - | - | 1 | 1 | - | - | - | - | - | - | - | - | - | - | - | - | - | - | 2 | 0.11 |
| GTG | - | - | 2 | - | - | - | - | - | - | - | - | - | - | - | - | - | - | - | 2 | 0.11 |
| TAA | - | - | 1 | - | - | - | - | 1 | - | - | - | - | - | - | - | - | - | - | 2 | 0.11 |
| AGT | - | - | 1 | - | - | - | - | - | - | - | - | - | - | - | - | - | - | - | 1 | 0.05 |
| CGA | - | - | - | - | 1 | - | - | - | - | - | - | - | - | - | - | - | - | - | 1 | 0.05 |
| CGG | - | - | 1 | - | - | - | - | - | - | - | - | - | - | - | - | - | - | - | 1 | 0.05 |
| CTA | - | - | 1 | - | - | - | - | - | - | - | - | - | - | - | - | - | - | - | 1 | 0.05 |
| GAC | - | - | 1 | - | - | - | - | - | - | - | - | - | - | - | - | - | - | - | 1 | 0.05 |
| TAG | - | - | 1 | - | - | - | - | - | - | - | - | - | - | - | - | - | - | - | 1 | 0.05 |
| TCG | - | - | - | 1 | - | - | - | - | - | - | - | - | - | - | - | - | - | - | 1 | 0.05 |
| AAAG | - | 10 | 2 | - | - | - | - | - | - | - | - | - | - | - | - | - | - | - | 12 | 0.63 |
| TTTC | - | 10 | - | 2 | - | - | - | - | - | - | - | - | - | - | - | - | - | - | 12 | 0.63 |
| CTTC | - | 7 | - | - | - | - | - | - | - | - | - | - | - | - | - | - | - | - | 7 | 0.37 |
| TCTT | - | 6 | - | - | 1 | - | - | - | - | - | - | - | - | - | - | - | - | - | 7 | 0.37 |
| TTAT | - | 7 | - | - | - | - | - | - | - | - | - | - | - | - | - | - | - | - | 7 | 0.37 |
| TTCT | - | 6 | 1 | - | - | - | - | - | - | - | - | - | - | - | - | - | - | - | 7 | 0.37 |
| ATTT | - | 5 | 1 | - | - | - | - | - | - | - | - | - | - | - | - | - | - | - | 6 | 0.32 |
| TTTA | - | 6 | - | - | - | - | - | - | - | - | - | - | - | - | - | - | - | - | 6 | 0.32 |
| AAGA | - | 5 | - | - | - | - | - | - | - | - | - | - | - | - | - | - | - | - | 5 | 0.26 |
| AGAA | - | 4 | - | 1 | - | - | - | - | - | - | - | - | - | - | - | - | - | - | 5 | 0.26 |
| CAAA | - | 5 | - | - | - | - | - | - | - | - | - | - | - | - | - | - | - | - | 5 | 0.26 |
| AAAT | - | 3 | 1 | - | - | - | - | - | - | - | - | - | - | - | - | - | - | - | 4 | 0.21 |
| AATC | - | 3 | 1 | - | - | - | - | - | - | - | - | - | - | - | - | - | - | - | 4 | 0.21 |
| AATT | - | 2 | 2 | - | - | - | - | - | - | - | - | - | - | - | - | - | - | - | 4 | 0.21 |
| GAAA | - | 4 | - | - | - | - | - | - | - | - | - | - | - | - | - | - | - | - | 4 | 0.21 |
| GATT | - | 3 | 1 | - | - | - | - | - | - | - | - | - | - | - | - | - | - | - | 4 | 0.21 |
| TAAA | - | 2 | 1 | 1 | - | - | - | - | - | - | - | - | - | - | - | - | - | - | 4 | 0.21 |
| AAAC | - | 3 | - | - | - | - | - | - | - | - | - | - | - | - | - | - | - | - | 3 | 0.16 |
| AACA | - | 3 | - | - | - | - | - | - | - | - | - | - | - | - | - | - | - | - | 3 | 0.16 |
| AAGC | - | 3 | - | - | - | - | - | - | - | - | - | - | - | - | - | - | - | - | 3 | 0.16 |
| ATAA | - | 3 | - | - | - | - | - | - | - | - | - | - | - | - | - | - | - | - | 3 | 0.16 |
| CTTG | - | 3 | - | - | - | - | - | - | - | - | - | - | - | - | - | - | - | - | 3 | 0.16 |
| TATG | - | 3 | - | - | - | - | - | - | - | - | - | - | - | - | - | - | - | - | 3 | 0.16 |
| TGCA | - | 3 | - | - | - | - | - | - | - | - | - | - | - | - | - | - | - | - | 3 | 0.16 |
| TGGT | - | 3 | - | - | - | - | - | - | - | - | - | - | - | - | - | - | - | - | 3 | 0.16 |
| TTAA | - | 3 | - | - | - | - | - | - | - | - | - | - | - | - | - | - | - | - | 3 | 0.16 |
| TTTG | - | 3 | - | - | - | - | - | - | - | - | - | - | - | - | - | - | - | - | 3 | 0.16 |
| AGCA | - | 2 | - | - | - | - | - | - | - | - | - | - | - | - | - | - | - | - | 2 | 0.11 |
| CTTT | - | 2 | - | - | - | - | - | - | - | - | - | - | - | - | - | - | - | - | 2 | 0.11 |
| GAAG | - | 2 | - | - | - | - | - | - | - | - | - | - | - | - | - | - | - | - | 2 | 0.11 |
| GCTT | - | 2 | - | - | - | - | - | - | - | - | - | - | - | - | - | - | - | - | 2 | 0.11 |
| GGAA | - | 2 | - | - | - | - | - | - | - | - | - | - | - | - | - | - | - | - | 2 | 0.11 |
| TACA | - | 1 | 1 | - | - | - | - | - | - | - | - | - | - | - | - | - | - | - | 2 | 0.11 |
| TGAT | - | 2 | - | - | - | - | - | - | - | - | - | - | - | - | - | - | - | - | 2 | 0.11 |
| AACC | - | 1 | - | - | - | - | - | - | - | - | - | - | - | - | - | - | - | - | 1 | 0.05 |
| AACT | - | 1 | - | - | - | - | - | - | - | - | - | - | - | - | - | - | - | - | 1 | 0.05 |
| AAGG | - | 1 | - | - | - | - | - | - | - | - | - | - | - | - | - | - | - | - | 1 | 0.05 |
| AATA | - | 1 | - | - | - | - | - | - | - | - | - | - | - | - | - | - | - | - | 1 | 0.05 |
| AATG | - | 1 | - | - | - | - | - | - | - | - | - | - | - | - | - | - | - | - | 1 | 0.05 |
| ACAT | - | 1 | - | - | - | - | - | - | - | - | - | - | - | - | - | - | - | - | 1 | 0.05 |
| ACCT | - | - | 1 | - | - | - | - | - | - | - | - | - | - | - | - | - | - | - | 1 | 0.05 |
| ACTT | - | 1 | - | - | - | - | - | - | - | - | - | - | - | - | - | - | - | - | 1 | 0.05 |
| AGAT | - | 1 | - | - | - | - | - | - | - | - | - | - | - | - | - | - | - | - | 1 | 0.05 |
| AGCG | - | 1 | - | - | - | - | - | - | - | - | - | - | - | - | - | - | - | - | 1 | 0.05 |
| AGGC | - | 1 | - | - | - | - | - | - | - | - | - | - | - | - | - | - | - | - | 1 | 0.05 |
| ATAC | - | - | 1 | - | - | - | - | - | - | - | - | - | - | - | - | - | - | - | 1 | 0.05 |
| ATAG | - | - | 1 | - | - | - | - | - | - | - | - | - | - | - | - | - | - | - | 1 | 0.05 |
| ATGA | - | - | 1 | - | - | - | - | - | - | - | - | - | - | - | - | - | - | - | 1 | 0.05 |
| ATGC | - | 1 | - | - | - | - | - | - | - | - | - | - | - | - | - | - | - | - | 1 | 0.05 |
| ATGT | - | 1 | - | - | - | - | - | - | - | - | - | - | - | - | - | - | - | - | 1 | 0.05 |
| ATTA | - | 1 | - | - | - | - | - | - | - | - | - | - | - | - | - | - | - | - | 1 | 0.05 |
| CAAC | - | 1 | - | - | - | - | - | - | - | - | - | - | - | - | - | - | - | - | 1 | 0.05 |
| CAAG | - | 1 | - | - | - | - | - | - | - | - | - | - | - | - | - | - | - | - | 1 | 0.05 |
| CACC | - | 1 | - | - | - | - | - | - | - | - | - | - | - | - | - | - | - | - | 1 | 0.05 |
| CAGC | - | 1 | - | - | - | - | - | - | - | - | - | - | - | - | - | - | - | - | 1 | 0.05 |
| CATA | - | 1 | - | - | - | - | - | - | - | - | - | - | - | - | - | - | - | - | 1 | 0.05 |
| CCCA | - | 1 | - | - | - | - | - | - | - | - | - | - | - | - | - | - | - | - | 1 | 0.05 |
| CCGG | - | 1 | - | - | - | - | - | - | - | - | - | - | - | - | - | - | - | - | 1 | 0.05 |
| CCTA | - | 1 | - | - | - | - | - | - | - | - | - | - | - | - | - | - | - | - | 1 | 0.05 |
| CGGC | - | 1 | - | - | - | - | - | - | - | - | - | - | - | - | - | - | - | - | 1 | 0.05 |
| CGGG | - | 1 | - | - | - | - | - | - | - | - | - | - | - | - | - | - | - | - | 1 | 0.05 |
| CGTT | - | 1 | - | - | - | - | - | - | - | - | - | - | - | - | - | - | - | - | 1 | 0.05 |
| CTAG | - | 1 | - | - | - | - | - | - | - | - | - | - | - | - | - | - | - | - | 1 | 0.05 |
| CTAT | - | 1 | - | - | - | - | - | - | - | - | - | - | - | - | - | - | - | - | 1 | 0.05 |
| CTCA | - | 1 | - | - | - | - | - | - | - | - | - | - | - | - | - | - | - | - | 1 | 0.05 |
| CTCC | - | 1 | - | - | - | - | - | - | - | - | - | - | - | - | - | - | - | - | 1 | 0.05 |
| CTGC | - | 1 | - | - | - | - | - | - | - | - | - | - | - | - | - | - | - | - | 1 | 0.05 |
| GAAT | - | 1 | - | - | - | - | - | - | - | - | - | - | - | - | - | - | - | - | 1 | 0.05 |
| GACC | - | 1 | - | - | - | - | - | - | - | - | - | - | - | - | - | - | - | - | 1 | 0.05 |
| GATC | - | 1 | - | - | - | - | - | - | - | - | - | - | - | - | - | - | - | - | 1 | 0.05 |
| GCAA | - | 1 | - | - | - | - | - | - | - | - | - | - | - | - | - | - | - | - | 1 | 0.05 |
| GCAG | - | 1 | - | - | - | - | - | - | - | - | - | - | - | - | - | - | - | - | 1 | 0.05 |
| GCAT | - | 1 | - | - | - | - | - | - | - | - | - | - | - | - | - | - | - | - | 1 | 0.05 |
| GCTA | - | 1 | - | - | - | - | - | - | - | - | - | - | - | - | - | - | - | - | 1 | 0.05 |
| GGAG | - | 1 | - | - | - | - | - | - | - | - | - | - | - | - | - | - | - | - | 1 | 0.05 |
| GGAT | - | 1 | - | - | - | - | - | - | - | - | - | - | - | - | - | - | - | - | 1 | 0.05 |
| GGTT | - | - | 1 | - | - | - | - | - | - | - | - | - | - | - | - | - | - | - | 1 | 0.05 |
| GTTC | - | 1 | - | - | - | - | - | - | - | - | - | - | - | - | - | - | - | - | 1 | 0.05 |
| GTTG | - | 1 | - | - | - | - | - | - | - | - | - | - | - | - | - | - | - | - | 1 | 0.05 |
| GTTT | - | 1 | - | - | - | - | - | - | - | - | - | - | - | - | - | - | - | - | 1 | 0.05 |
| TACC | - | 1 | - | - | - | - | - | - | - | - | - | - | - | - | - | - | - | - | 1 | 0.05 |
| TATT | - | 1 | - | - | - | - | - | - | - | - | - | - | - | - | - | - | - | - | 1 | 0.05 |
| TGAC | - | 1 | - | - | - | - | - | - | - | - | - | - | - | - | - | - | - | - | 1 | 0.05 |
| TGCT | - |  | 1 | - | - | - | - | - | - | - | - | - | - | - | - | - | - | - | 1 | 0.05 |
| TGGC | - | 1 | - | - | - | - | - | - | - | - | - | - | - | - | - | - | - | - | 1 | 0.05 |
| TGTA | - | 1 | - | - | - | - | - | - | - | - | - | - | - | - | - | - | - | - | 1 | 0.05 |
| TGTT | - | 1 | - | - | - | - | - | - | - | - | - | - | - | - | - | - | - | - | 1 | 0.05 |
| TTCC | - | 1 | - | - | - | - | - | - | - | - | - | - | - | - | - | - | - | - | 1 | 0.05 |
| TTGA | - | - | - | 1 | - | - | - | - | - | - | - | - | - | - | - | - | - | - | 1 | 0.05 |
| TTGT | - | - | - | 1 | - | - | - | - | - | - | - | - | - | - | - | - | - | - | 1 | 0.05 |
| AAAAG | - | 4 | - | - | - | - | - | - | - | - | - | - | - | - | - | - | - | - | 4 | 0.21 |
| AAAAT | - | 2 | - | - | - | - | - | - | - | - | - | - | - | - | - | - | - | - | 2 | 0.11 |
| GAAAA | - | 1 | 1 | - | - | - | - | - | - | - | - | - | - | - | - | - | - | - | 2 | 0.11 |
| AAAAC | - | - | 1 | - | - | - | - | - | - | - | - | - | - | - | - | - | - | - | 1 | 0.05 |
| AAAGG | - | 1 | - | - | - | - | - | - | - | - | - | - | - | - | - | - | - | - | 1 | 0.05 |
| AAATC | - | - | 1 | - | - | - | - | - | - | - | - | - | - | - | - | - | - | - | 1 | 0.05 |
| AAATT | - | 1 | - | - | - | - | - | - | - | - | - | - | - | - | - | - | - | - | 1 | 0.05 |
| AACTC | - | - | 1 | - | - | - | - | - | - | - | - | - | - | - | - | - | - | - | 1 | 0.05 |
| AAGGA | - | 1 | - | - | - | - | - | - | - | - | - | - | - | - | - | - | - | - | 1 | 0.05 |
| ACTAT | - | 1 | - | - | - | - | - | - | - | - | - | - | - | - | - | - | - | - | 1 | 0.05 |
| AGAAA | - | 1 | - | - | - | - | - | - | - | - | - | - | - | - | - | - | - | - | 1 | 0.05 |
| ATAAT | - | 1 | - | - | - | - | - | - | - | - | - | - | - | - | - | - | - | - | 1 | 0.05 |
| ATCAG | - | 1 | - | - | - | - | - | - | - | - | - | - | - | - | - | - | - | - | 1 | 0.05 |
| ATTTT | - | 1 | - | - | - | - | - | - | - | - | - | - | - | - | - | - | - | - | 1 | 0.05 |
| CAGAC | - | 1 | - | - | - | - | - | - | - | - | - | - | - | - | - | - | - | - | 1 | 0.05 |
| CCAGG | - | 1 | - | - | - | - | - | - | - | - | - | - | - | - | - | - | - | - | 1 | 0.05 |
| CTAGT | - | 1 | - | - | - | - | - | - | - | - | - | - | - | - | - | - | - | - | 1 | 0.05 |
| CTGCT | - | 1 | - | - | - | - | - | - | - | - | - | - | - | - | - | - | - | - | 1 | 0.05 |
| CTTCT | - | - | 1 | - | - | - | - | - | - | - | - | - | - | - | - | - | - | - | 1 | 0.05 |
| CTTTC | - | - | 1 | - | - | - | - | - | - | - | - | - | - | - | - | - | - | - | 1 | 0.05 |
| CTTTT | - | - | 1 | - | - | - | - | - | - | - | - | - | - | - | - | - | - | - | 1 | 0.05 |
| GAAAG | - | 1 | - | - | - | - | - | - | - | - | - | - | - | - | - | - | - | - | 1 | 0.05 |
| GAGCA | - | - | 1 | - | - | - | - | - | - | - | - | - | - | - | - | - | - | - | 1 | 0.05 |
| TCGGC | - | 1 | - | - | - | - | - | - | - | - | - | - | - | - | - | - | - | - | 1 | 0.05 |
| TCTCT | - | 1 | - | - | - | - | - | - | - | - | - | - | - | - | - | - | - | - | 1 | 0.05 |
| TGTTG | - | 1 | - | - | - | - | - | - | - | - | - | - | - | - | - | - | - | - | 1 | 0.05 |
| TTAAA | - | 1 | - | - | - | - | - | - | - | - | - | - | - | - | - | - | - | - | 1 | 0.05 |
| TTCTT | - | 1 | - | - | - | - | - | - | - | - | - | - | - | - | - | - | - | - | 1 | 0.05 |
| TTTAA | - | 1 | - | - | - | - | - | - | - | - | - | - | - | - | - | - | - | - | 1 | 0.05 |
| TTTAT | - | 1 | - | - | - | - | - | - | - | - | - | - | - | - | - | - | - | - | 1 | 0.05 |
| TTTCC | - | 1 | - | - | - | - | - | - | - | - | - | - | - | - | - | - | - | - | 1 | 0.05 |
| TTTTA | - | 1 | - | - | - | - | - | - | - | - | - | - | - | - | - | - | - | - | 1 | 0.05 |
| TTTTG | - | - | - | 1 | - | - | - | - | - | - | - | - | - | - | - | - | - | - | 1 | 0.05 |
| AAGCAA | - | - | - | 1 | - | - | - | - | - | - | - | - | - | - | - | - | - | - | 1 | 0.05 |
| AGCAAC | - | - | - | 1 | - | - | - | - | - | - | - | - | - | - | - | - | - | - | 1 | 0.05 |
| CGACTG | - | - | - | 1 | - | - | - | - | - | - | - | - | - | - | - | - | - | - | 1 | 0.05 |
| CTAATC | - | - | - | 1 | - | - | - | - | - | - | - | - | - | - | - | - | - | - | 1 | 0.05 |
| TCGTGA | - | - | - | 1 | - | - | - | - | - | - | - | - | - | - | - | - | - | - | 1 | 0.05 |
| TCTCCT | - | - | - | 1 | - | - | - | - | - | - | - | - | - | - | - | - | - | - | 1 | 0.05 |
| AAAAAAG | 4 | - | - | - | - | - | - | - | - | - | - | - | - | - | - | - | - | - | 4 | 0.21 |
| TTTTTCT | 4 | - | - | - | - | - | - | - | - | - | - | - | - | - | - | - | - | - | 4 | 0.21 |
| AAAACAA | 3 | - | - | - | - | - | - | - | - | - | - | - | - | - | - | - | - | - | 3 | 0.16 |
| AGAGAAA | 3 | - | - | - | - | - | - | - | - | - | - | - | - | - | - | - | - | - | 3 | 0.16 |
| ATCTTGG | 3 | - | - | - | - | - | - | - | - | - | - | - | - | - | - | - | - | - | 3 | 0.16 |
| TCCATCA | 3 | - | - | - | - | - | - | - | - | - | - | - | - | - | - | - | - | - | 3 | 0.16 |
| TTCTTCT | 3 | - | - | - | - | - | - | - | - | - | - | - | - | - | - | - | - | - | 3 | 0.16 |
| TTTTTTC | 3 | - | - | - | - | - | - | - | - | - | - | - | - | - | - | - | - | - | 3 | 0.16 |
| AAAAAAC | 2 | - | - | - | - | - | - | - | - | - | - | - | - | - | - | - | - | - | 2 | 0.11 |
| AAAAACC | 2 | - | - | - | - | - | - | - | - | - | - | - | - | - | - | - | - | - | 2 | 0.11 |
| AAAAATA | 2 | - | - | - | - | - | - | - | - | - | - | - | - | - | - | - | - | - | 2 | 0.11 |
| AAAAATT | 2 | - | - | - | - | - | - | - | - | - | - | - | - | - | - | - | - | - | 2 | 0.11 |
| AAAATAT | 2 | - | - | - | - | - | - | - | - | - | - | - | - | - | - | - | - | - | 2 | 0.11 |
| AAAGAAG | 2 | - | - | - | - | - | - | - | - | - | - | - | - | - | - | - | - | - | 2 | 0.11 |
| AAAGAGA | 2 | - | - | - | - | - | - | - | - | - | - | - | - | - | - | - | - | - | 2 | 0.11 |
| AAGAAAG | 2 | - | - | - | - | - | - | - | - | - | - | - | - | - | - | - | - | - | 2 | 0.11 |
| AAGAAGA | 2 | - | - | - | - | - | - | - | - | - | - | - | - | - | - | - | - | - | 2 | 0.11 |
| ACCATTC | 2 | - | - | - | - | - | - | - | - | - | - | - | - | - | - | - | - | - | 2 | 0.11 |
| AGAAAAA | 2 | - | - | - | - | - | - | - | - | - | - | - | - | - | - | - | - | - | 2 | 0.11 |
| AGAAAGA | 2 | - | - | - | - | - | - | - | - | - | - | - | - | - | - | - | - | - | 2 | 0.11 |
| AGAGAAT | 2 | - | - | - | - | - | - | - | - | - | - | - | - | - | - | - | - | - | 2 | 0.11 |
| ATGATTG | 2 | - | - | - | - | - | - | - | - | - | - | - | - | - | - | - | - | - | 2 | 0.11 |
| ATGTTGA | 2 | - | - | - | - | - | - | - | - | - | - | - | - | - | - | - | - | - | 2 | 0.11 |
| ATTGAGA | 2 | - | - | - | - | - | - | - | - | - | - | - | - | - | - | - | - | - | 2 | 0.11 |
| CAAAACT | 2 | - | - | - | - | - | - | - | - | - | - | - | - | - | - | - | - | - | 2 | 0.11 |
| CAAAATA | 2 | - | - | - | - | - | - | - | - | - | - | - | - | - | - | - | - | - | 2 | 0.11 |
| CATAATA | 2 | - | - | - | - | - | - | - | - | - | - | - | - | - | - | - | - | - | 2 | 0.11 |
| CCTCCTC | 2 | - | - | - | - | - | - | - | - | - | - | - | - | - | - | - | - | - | 2 | 0.11 |
| CTCCACA | 2 | - | - | - | - | - | - | - | - | - | - | - | - | - | - | - | - | - | 2 | 0.11 |
| CTCTCTC | 2 | - | - | - | - | - | - | - | - | - | - | - | - | - | - | - | - | - | 2 | 0.11 |
| GAAAATA | 2 | - | - | - | - | - | - | - | - | - | - | - | - | - | - | - | - | - | 2 | 0.11 |
| GCGACTC | 2 | - | - | - | - | - | - | - | - | - | - | - | - | - | - | - | - | - | 2 | 0.11 |
| GGGAGGA | 2 | - | - | - | - | - | - | - | - | - | - | - | - | - | - | - | - | - | 2 | 0.11 |
| TAAAAAA | 2 | - | - | - | - | - | - | - | - | - | - | - | - | - | - | - | - | - | 2 | 0.11 |
| TCATGGA | 2 | - | - | - | - | - | - | - | - | - | - | - | - | - | - | - | - | - | 2 | 0.11 |
| TCTTTTT | 2 | - | - | - | - | - | - | - | - | - | - | - | - | - | - | - | - | - | 2 | 0.11 |
| TGAAAAA | 2 | - | - | - | - | - | - | - | - | - | - | - | - | - | - | - | - | - | 2 | 0.11 |
| TGACTAA | 2 | - | - | - | - | - | - | - | - | - | - | - | - | - | - | - | - | - | 2 | 0.11 |
| TGGGTTT | 2 | - | - | - | - | - | - | - | - | - | - | - | - | - | - | - | - | - | 2 | 0.11 |
| TTAGATC | 2 | - | - | - | - | - | - | - | - | - | - | - | - | - | - | - | - | - | 2 | 0.11 |
| TTATTTA | 2 | - | - | - | - | - | - | - | - | - | - | - | - | - | - | - | - | - | 2 | 0.11 |
| TTATTTT | 2 | - | - | - | - | - | - | - | - | - | - | - | - | - | - | - | - | - | 2 | 0.11 |
| TTTCCTT | 2 | - | - | - | - | - | - | - | - | - | - | - | - | - | - | - | - | - | 2 | 0.11 |
| TTTTAAA | 2 | - | - | - | - | - | - | - | - | - | - | - | - | - | - | - | - | - | 2 | 0.11 |
| AAAAAAT | 1 | - | - | - | - | - | - | - | - | - | - | - | - | - | - | - | - | - | 1 | 0.05 |
| AAAAACA | 1 | - | - | - | - | - | - | - | - | - | - | - | - | - | - | - | - | - | 1 | 0.05 |
| AAAAACT | 1 | - | - | - | - | - | - | - | - | - | - | - | - | - | - | - | - | - | 1 | 0.05 |
| AAAAAGC | 1 | - | - | - | - | - | - | - | - | - | - | - | - | - | - | - | - | - | 1 | 0.05 |
| AAAAATG | 1 | - | - | - | - | - | - | - | - | - | - | - | - | - | - | - | - | - | 1 | 0.05 |
| AAAACAT | 1 | - | - | - | - | - | - | - | - | - | - | - | - | - | - | - | - | - | 1 | 0.05 |
| AAAACCA | 1 | - | - | - | - | - | - | - | - | - | - | - | - | - | - | - | - | - | 1 | 0.05 |
| AAAACTG | 1 | - | - | - | - | - | - | - | - | - | - | - | - | - | - | - | - | - | 1 | 0.05 |
| AAAAGAT | 1 | - | - | - | - | - | - | - | - | - | - | - | - | - | - | - | - | - | 1 | 0.05 |
| AAAATCA | 1 | - | - | - | - | - | - | - | - | - | - | - | - | - | - | - | - | - | 1 | 0.05 |
| AAAATCC | 1 | - | - | - | - | - | - | - | - | - | - | - | - | - | - | - | - | - | 1 | 0.05 |
| AAACAAC | 1 | - | - | - | - | - | - | - | - | - | - | - | - | - | - | - | - | - | 1 | 0.05 |
| AAACAAG | 1 | - | - | - | - | - | - | - | - | - | - | - | - | - | - | - | - | - | 1 | 0.05 |
| AAACAGA | 1 | - | - | - | - | - | - | - | - | - | - | - | - | - | - | - | - | - | 1 | 0.05 |
| AAACAGG | 1 | - | - | - | - | - | - | - | - | - | - | - | - | - | - | - | - | - | 1 | 0.05 |
| AAACATA | 1 | - | - | - | - | - | - | - | - | - | - | - | - | - | - | - | - | - | 1 | 0.05 |
| AAACCCT | 1 | - | - | - | - | - | - | - | - | - | - | - | - | - | - | - | - | - | 1 | 0.05 |
| AAACCTT | 1 | - | - | - | - | - | - | - | - | - | - | - | - | - | - | - | - | - | 1 | 0.05 |
| AAAGAAT | 1 | - | - | - | - | - | - | - | - | - | - | - | - | - | - | - | - | - | 1 | 0.05 |
| AAAGAGG | 1 | - | - | - | - | - | - | - | - | - | - | - | - | - | - | - | - | - | 1 | 0.05 |
| AAAGATT | 1 | - | - | - | - | - | - | - | - | - | - | - | - | - | - | - | - | - | 1 | 0.05 |
| AAAGGGA | 1 | - | - | - | - | - | - | - | - | - | - | - | - | - | - | - | - | - | 1 | 0.05 |
| AAAGTCA | 1 | - | - | - | - | - | - | - | - | - | - | - | - | - | - | - | - | - | 1 | 0.05 |
| AAAGTTG | 1 | - | - | - | - | - | - | - | - | - | - | - | - | - | - | - | - | - | 1 | 0.05 |
| AAATAAA | 1 | - | - | - | - | - | - | - | - | - | - | - | - | - | - | - | - | - | 1 | 0.05 |
| AAATCAA | 1 | - | - | - | - | - | - | - | - | - | - | - | - | - | - | - | - | - | 1 | 0.05 |
| AAATCCT | 1 | - | - | - | - | - | - | - | - | - | - | - | - | - | - | - | - | - | 1 | 0.05 |
| AAATGGG | 1 | - | - | - | - | - | - | - | - | - | - | - | - | - | - | - | - | - | 1 | 0.05 |
| AAATTCA | 1 | - | - | - | - | - | - | - | - | - | - | - | - | - | - | - | - | - | 1 | 0.05 |
| AAATTTT | 1 | - | - | - | - | - | - | - | - | - | - | - | - | - | - | - | - | - | 1 | 0.05 |
| AACAAAA | 1 | - | - | - | - | - | - | - | - | - | - | - | - | - | - | - | - | - | 1 | 0.05 |
| AACAAAC | 1 | - | - | - | - | - | - | - | - | - | - | - | - | - | - | - | - | - | 1 | 0.05 |
| AACAACA | 1 | - | - | - | - | - | - | - | - | - | - | - | - | - | - | - | - | - | 1 | 0.05 |
| AACACCA | 1 | - | - | - | - | - | - | - | - | - | - | - | - | - | - | - | - | - | 1 | 0.05 |
| AACATTT | 1 | - | - | - | - | - | - | - | - | - | - | - | - | - | - | - | - | - | 1 | 0.05 |
| AACCATT | 1 | - | - | - | - | - | - | - | - | - | - | - | - | - | - | - | - | - | 1 | 0.05 |
| AACCCAA | 1 | - | - | - | - | - | - | - | - | - | - | - | - | - | - | - | - | - | 1 | 0.05 |
| AACTTGG | 1 | - | - | - | - | - | - | - | - | - | - | - | - | - | - | - | - | - | 1 | 0.05 |
| AACTTTT | 1 | - | - | - | - | - | - | - | - | - | - | - | - | - | - | - | - | - | 1 | 0.05 |
| AAGAACA | 1 | - | - | - | - | - | - | - | - | - | - | - | - | - | - | - | - | - | 1 | 0.05 |
| AAGAGAG | 1 | - | - | - | - | - | - | - | - | - | - | - | - | - | - | - | - | - | 1 | 0.05 |
| AAGAGCA | 1 | - | - | - | - | - | - | - | - | - | - | - | - | - | - | - | - | - | 1 | 0.05 |
| AAGCAGG | 1 | - | - | - | - | - | - | - | - | - | - | - | - | - | - | - | - | - | 1 | 0.05 |
| AAGCTAA | 1 | - | - | - | - | - | - | - | - | - | - | - | - | - | - | - | - | - | 1 | 0.05 |
| AAGCTGA | 1 | - | - | - | - | - | - | - | - | - | - | - | - | - | - | - | - | - | 1 | 0.05 |
| AAGCTTT | 1 | - | - | - | - | - | - | - | - | - | - | - | - | - | - | - | - | - | 1 | 0.05 |
| AAGGAGA | 1 | - | - | - | - | - | - | - | - | - | - | - | - | - | - | - | - | - | 1 | 0.05 |
| AAGGGAA | 1 | - | - | - | - | - | - | - | - | - | - | - | - | - | - | - | - | - | 1 | 0.05 |
| AAGGGAT | 1 | - | - | - | - | - | - | - | - | - | - | - | - | - | - | - | - | - | 1 | 0.05 |
| AAGTCAA | 1 | - | - | - | - | - | - | - | - | - | - | - | - | - | - | - | - | - | 1 | 0.05 |
| AATAAAA | 1 | - | - | - | - | - | - | - | - | - | - | - | - | - | - | - | - | - | 1 | 0.05 |
| AATAAAT | 1 | - | - | - | - | - | - | - | - | - | - | - | - | - | - | - | - | - | 1 | 0.05 |
| AATCCCC |  | 1 | - | - | - | - | - | - | - | - | - | - | - | - | - | - | - | - | 1 | 0.05 |
| AATCTCA | 1 | - | - | - | - | - | - | - | - | - | - | - | - | - | - | - | - | - | 1 | 0.05 |
| AATGACA | 1 | - | - | - | - | - | - | - | - | - | - | - | - | - | - | - | - | - | 1 | 0.05 |
| AATTCGG | 1 | - | - | - | - | - | - | - | - | - | - | - | - | - | - | - | - | - | 1 | 0.05 |
| AATTCTA | 1 | - | - | - | - | - | - | - | - | - | - | - | - | - | - | - | - | - | 1 | 0.05 |
| AATTTAT | 1 | - | - | - | - | - | - | - | - | - | - | - | - | - | - | - | - | - | 1 | 0.05 |
| AATTTTC | 1 | - | - | - | - | - | - | - | - | - | - | - | - | - | - | - | - | - | 1 | 0.05 |
| AATTTTG | 1 | - | - | - | - | - | - | - | - | - | - | - | - | - | - | - | - | - | 1 | 0.05 |
| ACAAAAA | 1 | - | - | - | - | - | - | - | - | - | - | - | - | - | - | - | - | - | 1 | 0.05 |
| ACAAAGG | 1 | - | - | - | - | - | - | - | - | - | - | - | - | - | - | - | - | - | 1 | 0.05 |
| ACAAATG | 1 | - | - | - | - | - | - | - | - | - | - | - | - | - | - | - | - | - | 1 | 0.05 |
| ACAAGAT | 1 | - | - | - | - | - | - | - | - | - | - | - | - | - | - | - | - | - | 1 | 0.05 |
| ACATCTT | 1 | - | - | - | - | - | - | - | - | - | - | - | - | - | - | - | - | - | 1 | 0.05 |
| ACATTAT | 1 | - | - | - | - | - | - | - | - | - | - | - | - | - | - | - | - | - | 1 | 0.05 |
| ACATTCC | 1 | - | - | - | - | - | - | - | - | - | - | - | - | - | - | - | - | - | 1 | 0.05 |
| ACCAGGC | 1 | - | - | - | - | - | - | - | - | - | - | - | - | - | - | - | - | - | 1 | 0.05 |
| ACTAGAA | 1 | - | - | - | - | - | - | - | - | - | - | - | - | - | - | - | - | - | 1 | 0.05 |
| ACTATTG | 1 | - | - | - | - | - | - | - | - | - | - | - | - | - | - | - | - | - | 1 | 0.05 |
| ACTGAAA | 1 | - | - | - | - | - | - | - | - | - | - | - | - | - | - | - | - | - | 1 | 0.05 |
| ACTTCAA | 1 | - | - | - | - | - | - | - | - | - | - | - | - | - | - | - | - | - | 1 | 0.05 |
| AGAAAGC | 1 | - | - | - | - | - | - | - | - | - | - | - | - | - | - | - | - | - | 1 | 0.05 |
| AGAACCC | 1 | - | - | - | - | - | - | - | - | - | - | - | - | - | - | - | - | - | 1 | 0.05 |
| AGAAGAT | 1 | - | - | - | - | - | - | - | - | - | - | - | - | - | - | - | - | - | 1 | 0.05 |
| AGAAGGA | 1 | - | - | - | - | - | - | - | - | - | - | - | - | - | - | - | - | - | 1 | 0.05 |
| AGAGTCA | 1 | - | - | - | - | - | - | - | - | - | - | - | - | - | - | - | - | - | 1 | 0.05 |
| AGAGTTG | 1 | - | - | - | - | - | - | - | - | - | - | - | - | - | - | - | - | - | 1 | 0.05 |
| AGATTCA | 1 | - | - | - | - | - | - | - | - | - | - | - | - | - | - | - | - | - | 1 | 0.05 |
| AGCAAGA | 1 | - | - | - | - | - | - | - | - | - | - | - | - | - | - | - | - | - | 1 | 0.05 |
| AGCAATA | 1 | - | - | - | - | - | - | - | - | - | - | - | - | - | - | - | - | - | 1 | 0.05 |
| AGCAATG | 1 | - | - | - | - | - | - | - | - | - | - | - | - | - | - | - | - | - | 1 | 0.05 |
| AGCCCTT | 1 | - | - | - | - | - | - | - | - | - | - | - | - | - | - | - | - | - | 1 | 0.05 |
| AGCTATC | 1 | - | - | - | - | - | - | - | - | - | - | - | - | - | - | - | - | - | 1 | 0.05 |
| AGGAAAG | 1 | - | - | - | - | - | - | - | - | - | - | - | - | - | - | - | - | - | 1 | 0.05 |
| AGGAGAG | 1 | - | - | - | - | - | - | - | - | - | - | - | - | - | - | - | - | - | 1 | 0.05 |
| AGGATAT | 1 | - | - | - | - | - | - | - | - | - | - | - | - | - | - | - | - | - | 1 | 0.05 |
| AGGCGAG | 1 | - | - | - | - | - | - | - | - | - | - | - | - | - | - | - | - | - | 1 | 0.05 |
| AGGCTTC | 1 | - | - | - | - | - | - | - | - | - | - | - | - | - | - | - | - | - | 1 | 0.05 |
| AGGGAAA | 1 | - | - | - | - | - | - | - | - | - | - | - | - | - | - | - | - | - | 1 | 0.05 |
| AGGGGTG | 1 | - | - | - | - | - | - | - | - | - | - | - | - | - | - | - | - | - | 1 | 0.05 |
| AGGTTGC | 1 | - | - | - | - | - | - | - | - | - | - | - | - | - | - | - | - | - | 1 | 0.05 |
| AGTCATC | 1 | - | - | - | - | - | - | - | - | - | - | - | - | - | - | - | - | - | 1 | 0.05 |
| AGTTCAA | 1 | - | - | - | - | - | - | - | - | - | - | - | - | - | - | - | - | - | 1 | 0.05 |
| AGTTGTC | 1 | - | - | - | - | - | - | - | - | - | - | - | - | - | - | - | - | - | 1 | 0.05 |
| AGTTTGT | 1 | - | - | - | - | - | - | - | - | - | - | - | - | - | - | - | - | - | 1 | 0.05 |
| AGTTTTA | 1 | - | - | - | - | - | - | - | - | - | - | - | - | - | - | - | - | - | 1 | 0.05 |
| ATAAACC | 1 | - | - | - | - | - | - | - | - | - | - | - | - | - | - | - | - | - | 1 | 0.05 |
| ATAATAA | 1 | - | - | - | - | - | - | - | - | - | - | - | - | - | - | - | - | - | 1 | 0.05 |
| ATACTTG | 1 | - | - | - | - | - | - | - | - | - | - | - | - | - | - | - | - | - | 1 | 0.05 |
| ATAGAGA | 1 | - | - | - | - | - | - | - | - | - | - | - | - | - | - | - | - | - | 1 | 0.05 |
| ATATAAA | 1 | - | - | - | - | - | - | - | - | - | - | - | - | - | - | - | - | - | 1 | 0.05 |
| ATATATA | 1 | - | - | - | - | - | - | - | - | - | - | - | - | - | - | - | - | - | 1 | 0.05 |
| ATATATT | 1 | - | - | - | - | - | - | - | - | - | - | - | - | - | - | - | - | - | 1 | 0.05 |
| ATATCAA | 1 | - | - | - | - | - | - | - | - | - | - | - | - | - | - | - | - | - | 1 | 0.05 |
| ATATCTG | 1 | - | - | - | - | - | - | - | - | - | - | - | - | - | - | - | - | - | 1 | 0.05 |
| ATCAAAA | 1 | - | - | - | - | - | - | - | - | - | - | - | - | - | - | - | - | - | 1 | 0.05 |
| ATCACTG | 1 | - | - | - | - | - | - | - | - | - | - | - | - | - | - | - | - | - | 1 | 0.05 |
| ATCATAA | 1 | - | - | - | - | - | - | - | - | - | - | - | - | - | - | - | - | - | 1 | 0.05 |
| ATCATAG | 1 | - | - | - | - | - | - | - | - | - | - | - | - | - | - | - | - | - | 1 | 0.05 |
| ATCCATT | 1 | - | - | - | - | - | - | - | - | - | - | - | - | - | - | - | - | - | 1 | 0.05 |
| ATCCTTC | 1 | - | - | - | - | - | - | - | - | - | - | - | - | - | - | - | - | - | 1 | 0.05 |
| ATCCTTG | 1 | - | - | - | - | - | - | - | - | - | - | - | - | - | - | - | - | - | 1 | 0.05 |
| ATCTGCA | 1 | - | - | - | - | - | - | - | - | - | - | - | - | - | - | - | - | - | 1 | 0.05 |
| ATCTTTC | 1 | - | - | - | - | - | - | - | - | - | - | - | - | - | - | - | - | - | 1 | 0.05 |
| ATGACTT | 1 | - | - | - | - | - | - | - | - | - | - | - | - | - | - | - | - | - | 1 | 0.05 |
| ATGCAAC | 1 | - | - | - | - | - | - | - | - | - | - | - | - | - | - | - | - | - | 1 | 0.05 |
| ATGGAAA | 1 | - | - | - | - | - | - | - | - | - | - | - | - | - | - | - | - | - | 1 | 0.05 |
| ATTAAAA | 1 | - | - | - | - | - | - | - | - | - | - | - | - | - | - | - | - | - | 1 | 0.05 |
| ATTAGAA | 1 | - | - | - | - | - | - | - | - | - | - | - | - | - | - | - | - | - | 1 | 0.05 |
| ATTATGG | 1 | - | - | - | - | - | - | - | - | - | - | - | - | - | - | - | - | - | 1 | 0.05 |
| ATTATTA | 1 | - | - | - | - | - | - | - | - | - | - | - | - | - | - | - | - | - | 1 | 0.05 |
| ATTCTAA | 1 | - | - | - | - | - | - | - | - | - | - | - | - | - | - | - | - | - | 1 | 0.05 |
| ATTCTAG | 1 | - | - | - | - | - | - | - | - | - | - | - | - | - | - | - | - | - | 1 | 0.05 |
| ATTGGCA | 1 | - | - | - | - | - | - | - | - | - | - | - | - | - | - | - | - | - | 1 | 0.05 |
| ATTGGTG | 1 | - | - | - | - | - | - | - | - | - | - | - | - | - | - | - | - | - | 1 | 0.05 |
| ATTGTCA | 1 | - | - | - | - | - | - | - | - | - | - | - | - | - | - | - | - | - | 1 | 0.05 |
| ATTTAAG | 1 | - | - | - | - | - | - | - | - | - | - | - | - | - | - | - | - | - | 1 | 0.05 |
| ATTTTTA | 1 | - | - | - | - | - | - | - | - | - | - | - | - | - | - | - | - | - | 1 | 0.05 |
| CAAAAAA | 1 | - | - | - | - | - | - | - | - | - | - | - | - | - | - | - | - | - | 1 | 0.05 |
| CAAAAAG | 1 | - | - | - | - | - | - | - | - | - | - | - | - | - | - | - | - | - | 1 | 0.05 |
| CAAAAGC | 1 | - | - | - | - | - | - | - | - | - | - | - | - | - | - | - | - | - | 1 | 0.05 |
| CAAAAGG | 1 | - | - | - | - | - | - | - | - | - | - | - | - | - | - | - | - | - | 1 | 0.05 |
| CAAACAC | 1 | - | - | - | - | - | - | - | - | - | - | - | - | - | - | - | - | - | 1 | 0.05 |
| CAAACAT | 1 | - | - | - | - | - | - | - | - | - | - | - | - | - | - | - | - | - | 1 | 0.05 |
| CAAAGAA | 1 | - | - | - | - | - | - | - | - | - | - | - | - | - | - | - | - | - | 1 | 0.05 |
| CAAAGGC | 1 | - | - | - | - | - | - | - | - | - | - | - | - | - | - | - | - | - | 1 | 0.05 |
| CAACTCC | 1 | - | - | - | - | - | - | - | - | - | - | - | - | - | - | - | - | - | 1 | 0.05 |
| CAAGAAA | 1 | - | - | - | - | - | - | - | - | - | - | - | - | - | - | - | - | - | 1 | 0.05 |
| CAAGAAT | 1 | - | - | - | - | - | - | - | - | - | - | - | - | - | - | - | - | - | 1 | 0.05 |
| CAAGGCC | 1 | - | - | - | - | - | - | - | - | - | - | - | - | - | - | - | - | - | 1 | 0.05 |
| CAAGTTT | 1 | - | - | - | - | - | - | - | - | - | - | - | - | - | - | - | - | - | 1 | 0.05 |
| CAATGAC | 1 | - | - | - | - | - | - | - | - | - | - | - | - | - | - | - | - | - | 1 | 0.05 |
| CAATGTA | 1 | - | - | - | - | - | - | - | - | - | - | - | - | - | - | - | - | - | 1 | 0.05 |
| CAATTCT | 1 | - | - | - | - | - | - | - | - | - | - | - | - | - | - | - | - | - | 1 | 0.05 |
| CACAACT | 1 | - | - | - | - | - | - | - | - | - | - | - | - | - | - | - | - | - | 1 | 0.05 |
| CACACAA | 1 | - | - | - | - | - | - | - | - | - | - | - | - | - | - | - | - | - | 1 | 0.05 |
| CACCACA | 1 | - | - | - | - | - | - | - | - | - | - | - | - | - | - | - | - | - | 1 | 0.05 |
| CACCACC | 1 | - | - | - | - | - | - | - | - | - | - | - | - | - | - | - | - | - | 1 | 0.05 |
| CACTCTC | 1 | - | - | - | - | - | - | - | - | - | - | - | - | - | - | - | - | - | 1 | 0.05 |
| CACTGGT | 1 | - | - | - | - | - | - | - | - | - | - | - | - | - | - | - | - | - | 1 | 0.05 |
| CAGCGGA | 1 | - | - | - | - | - | - | - | - | - | - | - | - | - | - | - | - | - | 1 | 0.05 |
| CAGCTGC | 1 | - | - | - | - | - | - | - | - | - | - | - | - | - | - | - | - | - | 1 | 0.05 |
| CAGCTTC | 1 | - | - | - | - | - | - | - | - | - | - | - | - | - | - | - | - | - | 1 | 0.05 |
| CAGGGTA | 1 | - | - | - | - | - | - | - | - | - | - | - | - | - | - | - | - | - | 1 | 0.05 |
| CATATAT | 1 | - | - | - | - | - | - | - | - | - | - | - | - | - | - | - | - | - | 1 | 0.05 |
| CATATCC | 1 | - | - | - | - | - | - | - | - | - | - | - | - | - | - | - | - | - | 1 | 0.05 |
| CATCAAA | 1 | - | - | - | - | - | - | - | - | - | - | - | - | - | - | - | - | - | 1 | 0.05 |
| CATCAGC | 1 | - | - | - | - | - | - | - | - | - | - | - | - | - | - | - | - | - | 1 | 0.05 |
| CATGCAT | 1 | - | - | - | - | - | - | - | - | - | - | - | - | - | - | - | - | - | 1 | 0.05 |
| CATGCTT | 1 | - | - | - | - | - | - | - | - | - | - | - | - | - | - | - | - | - | 1 | 0.05 |
| CATGGTC | 1 | - | - | - | - | - | - | - | - | - | - | - | - | - | - | - | - | - | 1 | 0.05 |
| CCAAAAC | 1 | - | - | - | - | - | - | - | - | - | - | - | - | - | - | - | - | - | 1 | 0.05 |
| CCAACCC | 1 | - | - | - | - | - | - | - | - | - | - | - | - | - | - | - | - | - | 1 | 0.05 |
| CCAATAA | 1 | - | - | - | - | - | - | - | - | - | - | - | - | - | - | - | - | - | 1 | 0.05 |
| CCACATT | 1 | - | - | - | - | - | - | - | - | - | - | - | - | - | - | - | - | - | 1 | 0.05 |
| CCACCCC | 1 | - | - | - | - | - | - | - | - | - | - | - | - | - | - | - | - | - | 1 | 0.05 |
| CCAGAAT | 1 | - | - | - | - | - | - | - | - | - | - | - | - | - | - | - | - | - | 1 | 0.05 |
| CCAGATC | 1 | - | - | - | - | - | - | - | - | - | - | - | - | - | - | - | - | - | 1 | 0.05 |
| CCAGCAC | 1 | - | - | - | - | - | - | - | - | - | - | - | - | - | - | - | - | - | 1 | 0.05 |
| CCATCCC | 1 | - | - | - | - | - | - | - | - | - | - | - | - | - | - | - | - | - | 1 | 0.05 |
| CCATCTT | 1 | - | - | - | - | - | - | - | - | - | - | - | - | - | - | - | - | - | 1 | 0.05 |
| CCATGAC | 1 | - | - | - | - | - | - | - | - | - | - | - | - | - | - | - | - | - | 1 | 0.05 |
| CCATTCC | 1 | - | - | - | - | - | - | - | - | - | - | - | - | - | - | - | - | - | 1 | 0.05 |
| CCCAAGA | 1 | - | - | - | - | - | - | - | - | - | - | - | - | - | - | - | - | - | 1 | 0.05 |
| CCCAAGC | 1 | - | - | - | - | - | - | - | - | - | - | - | - | - | - | - | - | - | 1 | 0.05 |
| CCCATTA | 1 | - | - | - | - | - | - | - | - | - | - | - | - | - | - | - | - | - | 1 | 0.05 |
| CCCCCAC | 1 | - | - | - | - | - | - | - | - | - | - | - | - | - | - | - | - | - | 1 | 0.05 |
| CCCTGAC | 1 | - | - | - | - | - | - | - | - | - | - | - | - | - | - | - | - | - | 1 | 0.05 |
| CCTCAAC | 1 | - | - | - | - | - | - | - | - | - | - | - | - | - | - | - | - | - | 1 | 0.05 |
| CCTCTCT | 1 | - | - | - | - | - | - | - | - | - | - | - | - | - | - | - | - | - | 1 | 0.05 |
| CCTGAAT | 1 | - | - | - | - | - | - | - | - | - | - | - | - | - | - | - | - | - | 1 | 0.05 |
| CCTTACT | 1 | - | - | - | - | - | - | - | - | - | - | - | - | - | - | - | - | - | 1 | 0.05 |
| CCTTGAT | 1 | - | - | - | - | - | - | - | - | - | - | - | - | - | - | - | - | - | 1 | 0.05 |
| CCTTTGC | 1 | - | - | - | - | - | - | - | - | - | - | - | - | - | - | - | - | - | 1 | 0.05 |
| CGAATTC | 1 | - | - | - | - | - | - | - | - | - | - | - | - | - | - | - | - | - | 1 | 0.05 |
| CGGATGC | 1 | - | - | - | - | - | - | - | - | - | - | - | - | - | - | - | - | - | 1 | 0.05 |
| CGTTTGG | 1 | - | - | - | - | - | - | - | - | - | - | - | - | - | - | - | - | - | 1 | 0.05 |
| CTAAAAC | 1 | - | - | - | - | - | - | - | - | - | - | - | - | - | - | - | - | - | 1 | 0.05 |
| CTAAAGC | 1 | - | - | - | - | - | - | - | - | - | - | - | - | - | - | - | - | - | 1 | 0.05 |
| CTAGTTC | 1 | - | - | - | - | - | - | - | - | - | - | - | - | - | - | - | - | - | 1 | 0.05 |
| CTCATAA | 1 | - | - | - | - | - | - | - | - | - | - | - | - | - | - | - | - | - | 1 | 0.05 |
| CTCATCC | 1 | - | - | - | - | - | - | - | - | - | - | - | - | - | - | - | - | - | 1 | 0.05 |
| CTCATTC | 1 | - | - | - | - | - | - | - | - | - | - | - | - | - | - | - | - | - | 1 | 0.05 |
| CTCCAAG | 1 | - | - | - | - | - | - | - | - | - | - | - | - | - | - | - | - | - | 1 | 0.05 |
| CTCCATC | 1 | - | - | - | - | - | - | - | - | - | - | - | - | - | - | - | - | - | 1 | 0.05 |
| CTCCTCT | 1 | - | - | - | - | - | - | - | - | - | - | - | - | - | - | - | - | - | 1 | 0.05 |
| CTCTTCA | 1 | - | - | - | - | - | - | - | - | - | - | - | - | - | - | - | - | - | 1 | 0.05 |
| CTCTTTG | 1 | - | - | - | - | - | - | - | - | - | - | - | - | - | - | - | - | - | 1 | 0.05 |
| CTGAAAG | 1 | - | - | - | - | - | - | - | - | - | - | - | - | - | - | - | - | - | 1 | 0.05 |
| CTGAGGG | 1 | - | - | - | - | - | - | - | - | - | - | - | - | - | - | - | - | - | 1 | 0.05 |
| CTGCTCT | 1 | - | - | - | - | - | - | - | - | - | - | - | - | - | - | - | - | - | 1 | 0.05 |
| CTGGAAA | 1 | - | - | - | - | - | - | - | - | - | - | - | - | - | - | - | - | - | 1 | 0.05 |
| CTGGTGC | 1 | - | - | - | - | - | - | - | - | - | - | - | - | - | - | - | - | - | 1 | 0.05 |
| CTGTATT | 1 | - | - | - | - | - | - | - | - | - | - | - | - | - | - | - | - | - | 1 | 0.05 |
| CTGTGAA | 1 | - | - | - | - | - | - | - | - | - | - | - | - | - | - | - | - | - | 1 | 0.05 |
| CTGTGTC | 1 | - | - | - | - | - | - | - | - | - | - | - | - | - | - | - | - | - | 1 | 0.05 |
| CTGTTAA | 1 | - | - | - | - | - | - | - | - | - | - | - | - | - | - | - | - | - | 1 | 0.05 |
| CTGTTAG | 1 | - | - | - | - | - | - | - | - | - | - | - | - | - | - | - | - | - | 1 | 0.05 |
| CTTAGAT | 1 | - | - | - | - | - | - | - | - | - | - | - | - | - | - | - | - | - | 1 | 0.05 |
| CTTATTC | 1 | - | - | - | - | - | - | - | - | - | - | - | - | - | - | - | - | - | 1 | 0.05 |
| CTTCATG | 1 | - | - | - | - | - | - | - | - | - | - | - | - | - | - | - | - | - | 1 | 0.05 |
| CTTCGAT | 1 | - | - | - | - | - | - | - | - | - | - | - | - | - | - | - | - | - | 1 | 0.05 |
| CTTCTTC | 1 | - | - | - | - | - | - | - | - | - | - | - | - | - | - | - | - | - | 1 | 0.05 |
| CTTCTTG | 1 | - | - | - | - | - | - | - | - | - | - | - | - | - | - | - | - | - | 1 | 0.05 |
| CTTCTTT | 1 | - | - | - | - | - | - | - | - | - | - | - | - | - | - | - | - | - | 1 | 0.05 |
| CTTGGCA | 1 | - | - | - | - | - | - | - | - | - | - | - | - | - | - | - | - | - | 1 | 0.05 |
| CTTGTGA | 1 | - | - | - | - | - | - | - | - | - | - | - | - | - | - | - | - | - | 1 | 0.05 |
| CTTTAAG | 1 | - | - | - | - | - | - | - | - | - | - | - | - | - | - | - | - | - | 1 | 0.05 |
| CTTTAGT | 1 | - | - | - | - | - | - | - | - | - | - | - | - | - | - | - | - | - | 1 | 0.05 |
| CTTTATC | 1 | - | - | - | - | - | - | - | - | - | - | - | - | - | - | - | - | - | 1 | 0.05 |
| CTTTTCC | 1 | - | - | - | - | - | - | - | - | - | - | - | - | - | - | - | - | - | 1 | 0.05 |
| CTTTTGC | 1 | - | - | - | - | - | - | - | - | - | - | - | - | - | - | - | - | - | 1 | 0.05 |
| GAAAAAA | 1 | - | - | - | - | - | - | - | - | - | - | - | - | - | - | - | - | - | 1 | 0.05 |
| GAAAAAG | 1 | - | - | - | - | - | - | - | - | - | - | - | - | - | - | - | - | - | 1 | 0.05 |
| GAAAAAT | 1 | - | - | - | - | - | - | - | - | - | - | - | - | - | - | - | - | - | 1 | 0.05 |
| GAAAACA | 1 | - | - | - | - | - | - | - | - | - | - | - | - | - | - | - | - | - | 1 | 0.05 |
| GAAAACC | 1 | - | - | - | - | - | - | - | - | - | - | - | - | - | - | - | - | - | 1 | 0.05 |
| GAAACAA | 1 | - | - | - | - | - | - | - | - | - | - | - | - | - | - | - | - | - | 1 | 0.05 |
| GAACAGG | 1 | - | - | - | - | - | - | - | - | - | - | - | - | - | - | - | - | - | 1 | 0.05 |
| GAAGAAG | 1 | - | - | - | - | - | - | - | - | - | - | - | - | - | - | - | - | - | 1 | 0.05 |
| GAAGAGC | 1 | - | - | - | - | - | - | - | - | - | - | - | - | - | - | - | - | - | 1 | 0.05 |
| GAAGATC | 1 | - | - | - | - | - | - | - | - | - | - | - | - | - | - | - | - | - | 1 | 0.05 |
| GAAGATG | 1 | - | - | - | - | - | - | - | - | - | - | - | - | - | - | - | - | - | 1 | 0.05 |
| GAAGGAA | 1 | - | - | - | - | - | - | - | - | - | - | - | - | - | - | - | - | - | 1 | 0.05 |
| GAAGGTC | 1 | - | - | - | - | - | - | - | - | - | - | - | - | - | - | - | - | - | 1 | 0.05 |
| GAATCCT | 1 | - | - | - | - | - | - | - | - | - | - | - | - | - | - | - | - | - | 1 | 0.05 |
| GAATGAA | 1 | - | - | - | - | - | - | - | - | - | - | - | - | - | - | - | - | - | 1 | 0.05 |
| GACAAAA | 1 | - | - | - | - | - | - | - | - | - | - | - | - | - | - | - | - | - | 1 | 0.05 |
| GACCACT | 1 | - | - | - | - | - | - | - | - | - | - | - | - | - | - | - | - | - | 1 | 0.05 |
| GACCCCG | 1 | - | - | - | - | - | - | - | - | - | - | - | - | - | - | - | - | - | 1 | 0.05 |
| GACTATT | 1 | - | - | - | - | - | - | - | - | - | - | - | - | - | - | - | - | - | 1 | 0.05 |
| GAGAAAA | 1 | - | - | - | - | - | - | - | - | - | - | - | - | - | - | - | - | - | 1 | 0.05 |
| GAGAAAC | 1 | - | - | - | - | - | - | - | - | - | - | - | - | - | - | - | - | - | 1 | 0.05 |
| GAGAATG | 1 | - | - | - | - | - | - | - | - | - | - | - | - | - | - | - | - | - | 1 | 0.05 |
| GAGAGAA | 1 | - | - | - | - | - | - | - | - | - | - | - | - | - | - | - | - | - | 1 | 0.05 |
| GAGAGAG | 1 | - | - | - | - | - | - | - | - | - | - | - | - | - | - | - | - | - | 1 | 0.05 |
| GAGCCCC | 1 | - | - | - | - | - | - | - | - | - | - | - | - | - | - | - | - | - | 1 | 0.05 |
| GAGGCAA | 1 | - | - | - | - | - | - | - | - | - | - | - | - | - | - | - | - | - | 1 | 0.05 |
| GAGTCTC | 1 | - | - | - | - | - | - | - | - | - | - | - | - | - | - | - | - | - | 1 | 0.05 |
| GATCAAG | 1 | - | - | - | - | - | - | - | - | - | - | - | - | - | - | - | - | - | 1 | 0.05 |
| GATTCTA | 1 | - | - | - | - | - | - | - | - | - | - | - | - | - | - | - | - | - | 1 | 0.05 |
| GATTGAT | 1 | - | - | - | - | - | - | - | - | - | - | - | - | - | - | - | - | - | 1 | 0.05 |
| GATTGGT | 1 | - | - | - | - | - | - | - | - | - | - | - | - | - | - | - | - | - | 1 | 0.05 |
| GATTGTC | 1 | - | - | - | - | - | - | - | - | - | - | - | - | - | - | - | - | - | 1 | 0.05 |
| GATTTCA | 1 | - | - | - | - | - | - | - | - | - | - | - | - | - | - | - | - | - | 1 | 0.05 |
| GCAAAGG | 1 | - | - | - | - | - | - | - | - | - | - | - | - | - | - | - | - | - | 1 | 0.05 |
| GCACAAG | 1 | - | - | - | - | - | - | - | - | - | - | - | - | - | - | - | - | - | 1 | 0.05 |
| GCAGACA | 1 | - | - | - | - | - | - | - | - | - | - | - | - | - | - | - | - | - | 1 | 0.05 |
| GCAGAGC | 1 | - | - | - | - | - | - | - | - | - | - | - | - | - | - | - | - | - | 1 | 0.05 |
| GCAGCTG | 1 | - | - | - | - | - | - | - | - | - | - | - | - | - | - | - | - | - | 1 | 0.05 |
| GCCATGT | 1 | - | - | - | - | - | - | - | - | - | - | - | - | - | - | - | - | - | 1 | 0.05 |
| GCCTCTG | 1 | - | - | - | - | - | - | - | - | - | - | - | - | - | - | - | - | - | 1 | 0.05 |
| GCTACGG | 1 | - | - | - | - | - | - | - | - | - | - | - | - | - | - | - | - | - | 1 | 0.05 |
| GCTGCCT | 1 | - | - | - | - | - | - | - | - | - | - | - | - | - | - | - | - | - | 1 | 0.05 |
| GCTTACA | 1 | - | - | - | - | - | - | - | - | - | - | - | - | - | - | - | - | - | 1 | 0.05 |
| GGAAAAA | 1 | - | - | - | - | - | - | - | - | - | - | - | - | - | - | - | - | - | 1 | 0.05 |
| GGAAAAG | 1 | - | - | - | - | - | - | - | - | - | - | - | - | - | - | - | - | - | 1 | 0.05 |
| GGAAGAA | 1 | - | - | - | - | - | - | - | - | - | - | - | - | - | - | - | - | - | 1 | 0.05 |
| GGACACG | 1 | - | - | - | - | - | - | - | - | - | - | - | - | - | - | - | - | - | 1 | 0.05 |
| GGAGAAA | 1 | - | - | - | - | - | - | - | - | - | - | - | - | - | - | - | - | - | 1 | 0.05 |
| GGAGAAG | 1 | - | - | - | - | - | - | - | - | - | - | - | - | - | - | - | - | - | 1 | 0.05 |
| GGATACT | 1 | - | - | - | - | - | - | - | - | - | - | - | - | - | - | - | - | - | 1 | 0.05 |
| GGATAGT | 1 | - | - | - | - | - | - | - | - | - | - | - | - | - | - | - | - | - | 1 | 0.05 |
| GGATCCT | 1 | - | - | - | - | - | - | - | - | - | - | - | - | - | - | - | - | - | 1 | 0.05 |
| GGATTTG |  | - | 1 | - | - | - | - | - | - | - | - | - | - | - | - | - | - | - | 1 | 0.05 |
| GGCAATG | 1 | - | - | - | - | - | - | - | - | - | - | - | - | - | - | - | - | - | 1 | 0.05 |
| GGCTGTT | 1 | - | - | - | - | - | - | - | - | - | - | - | - | - | - | - | - | - | 1 | 0.05 |
| GGCTTGG | 1 | - | - | - | - | - | - | - | - | - | - | - | - | - | - | - | - | - | 1 | 0.05 |
| GGGAAGG | 1 | - | - | - | - | - | - | - | - | - | - | - | - | - | - | - | - | - | 1 | 0.05 |
| GGGGAGG | - | 1 | - | - | - | - | - | - | - | - | - | - | - | - | - | - | - | - | 1 | 0.05 |
| GGGGCAG | 1 | - | - | - | - | - | - | - | - | - | - | - | - | - | - | - | - | - | 1 | 0.05 |
| GGGTTTA | 1 | - | - | - | - | - | - | - | - | - | - | - | - | - | - | - | - | - | 1 | 0.05 |
| GGTGATG | 1 | - | - | - | - | - | - | - | - | - | - | - | - | - | - | - | - | - | 1 | 0.05 |
| GGTGATT | 1 | - | - | - | - | - | - | - | - | - | - | - | - | - | - | - | - | - | 1 | 0.05 |
| GGTGGAG | 1 | - | - | - | - | - | - | - | - | - | - | - | - | - | - | - | - | - | 1 | 0.05 |
| GGTTTTT | 1 | - | - | - | - | - | - | - | - | - | - | - | - | - | - | - | - | - | 1 | 0.05 |
| GTAACTA | 1 | - | - | - | - | - | - | - | - | - | - | - | - | - | - | - | - | - | 1 | 0.05 |
| GTAATCC | 1 | - | - | - | - | - | - | - | - | - | - | - | - | - | - | - | - | - | 1 | 0.05 |
| GTCAGGC | 1 | - | - | - | - | - | - | - | - | - | - | - | - | - | - | - | - | - | 1 | 0.05 |
| GTCATCA | 1 | - | - | - | - | - | - | - | - | - | - | - | - | - | - | - | - | - | 1 | 0.05 |
| GTCCTTG | 1 | - | - | - | - | - | - | - | - | - | - | - | - | - | - | - | - | - | 1 | 0.05 |
| GTGACTT | 1 | - | - | - | - | - | - | - | - | - | - | - | - | - | - | - | - | - | 1 | 0.05 |
| GTGGAAA | 1 | - | - | - | - | - | - | - | - | - | - | - | - | - | - | - | - | - | 1 | 0.05 |
| GTGGCTT | 1 | - | - | - | - | - | - | - | - | - | - | - | - | - | - | - | - | - | 1 | 0.05 |
| GTGGTAT | 1 | - | - | - | - | - | - | - | - | - | - | - | - | - | - | - | - | - | 1 | 0.05 |
| GTGTCAA | 1 | - | - | - | - | - | - | - | - | - | - | - | - | - | - | - | - | - | 1 | 0.05 |
| GTTAGCA | 1 | - | - | - | - | - | - | - | - | - | - | - | - | - | - | - | - | - | 1 | 0.05 |
| GTTCATT | 1 | - | - | - | - | - | - | - | - | - | - | - | - | - | - | - | - | - | 1 | 0.05 |
| GTTCCAT | 1 | - | - | - | - | - | - | - | - | - | - | - | - | - | - | - | - | - | 1 | 0.05 |
| GTTCGTT | 1 | - | - | - | - | - | - | - | - | - | - | - | - | - | - | - | - | - | 1 | 0.05 |
| GTTGCAA | 1 | - | - | - | - | - | - | - | - | - | - | - | - | - | - | - | - | - | 1 | 0.05 |
| GTTGCTC | 1 | - | - | - | - | - | - | - | - | - | - | - | - | - | - | - | - | - | 1 | 0.05 |
| GTTGGCT | 1 | - | - | - | - | - | - | - | - | - | - | - | - | - | - | - | - | - | 1 | 0.05 |
| GTTGTGA | 1 | - | - | - | - | - | - | - | - | - | - | - | - | - | - | - | - | - | 1 | 0.05 |
| GTTTCAA | 1 | - | - | - | - | - | - | - | - | - | - | - | - | - | - | - | - | - | 1 | 0.05 |
| GTTTGCA | 1 | - | - | - | - | - | - | - | - | - | - | - | - | - | - | - | - | - | 1 | 0.05 |
| GTTTTTG | 1 | - | - | - | - | - | - | - | - | - | - | - | - | - | - | - | - | - | 1 | 0.05 |
| TAAAACA | 1 | - | - | - | - | - | - | - | - | - | - | - | - | - | - | - | - | - | 1 | 0.05 |
| TAAAACT | 1 | - | - | - | - | - | - | - | - | - | - | - | - | - | - | - | - | - | 1 | 0.05 |
| TAAACCG | 1 | - | - | - | - | - | - | - | - | - | - | - | - | - | - | - | - | - | 1 | 0.05 |
| TAAAGAG | 1 | - | - | - | - | - | - | - | - | - | - | - | - | - | - | - | - | - | 1 | 0.05 |
| TAAATAT | 1 | - | - | - | - | - | - | - | - | - | - | - | - | - | - | - | - | - | 1 | 0.05 |
| TAAGGAG | 1 | - | - | - | - | - | - | - | - | - | - | - | - | - | - | - | - | - | 1 | 0.05 |
| TAATTAC | 1 | - | - | - | - | - | - | - | - | - | - | - | - | - | - | - | - | - | 1 | 0.05 |
| TAATTTA | 1 | - | - | - | - | - | - | - | - | - | - | - | - | - | - | - | - | - | 1 | 0.05 |
| TACATAT | 1 | - | - | - | - | - | - | - | - | - | - | - | - | - | - | - | - | - | 1 | 0.05 |
| TACTAGC | 1 | - | - | - | - | - | - | - | - | - | - | - | - | - | - | - | - | - | 1 | 0.05 |
| TAGCAGT | 1 | - | - | - | - | - | - | - | - | - | - | - | - | - | - | - | - | - | 1 | 0.05 |
| TAGTTTT | 1 | - | - | - | - | - | - | - | - | - | - | - | - | - | - | - | - | - | 1 | 0.05 |
| TATAAAA | 1 | - | - | - | - | - | - | - | - | - | - | - | - | - | - | - | - | - | 1 | 0.05 |
| TATAAAC | 1 | - | - | - | - | - | - | - | - | - | - | - | - | - | - | - | - | - | 1 | 0.05 |
| TATACGG | 1 | - | - | - | - | - | - | - | - | - | - | - | - | - | - | - | - | - | 1 | 0.05 |
| TATGTAA | 1 | - | - | - | - | - | - | - | - | - | - | - | - | - | - | - | - | - | 1 | 0.05 |
| TATTGAT | 1 | - | - | - | - | - | - | - | - | - | - | - | - | - | - | - | - | - | 1 | 0.05 |
| TATTGGT | 1 | - | - | - | - | - | - | - | - | - | - | - | - | - | - | - | - | - | 1 | 0.05 |
| TATTTAC | 1 | - | - | - | - | - | - | - | - | - | - | - | - | - | - | - | - | - | 1 | 0.05 |
| TATTTAT | 1 | - | - | - | - | - | - | - | - | - | - | - | - | - | - | - | - | - | 1 | 0.05 |
| TATTTGT | 1 | - | - | - | - | - | - | - | - | - | - | - | - | - | - | - | - | - | 1 | 0.05 |
| TATTTTC | 1 | - | - | - | - | - | - | - | - | - | - | - | - | - | - | - | - | - | 1 | 0.05 |
| TCAAAAC | 1 | - | - | - | - | - | - | - | - | - | - | - | - | - | - | - | - | - | 1 | 0.05 |
| TCAAAAT | 1 | - | - | - | - | - | - | - | - | - | - | - | - | - | - | - | - | - | 1 | 0.05 |
| TCAAACC | 1 | - | - | - | - | - | - | - | - | - | - | - | - | - | - | - | - | - | 1 | 0.05 |
| TCAACTA | 1 | - | - | - | - | - | - | - | - | - | - | - | - | - | - | - | - | - | 1 | 0.05 |
| TCAAGTT | 1 | - | - | - | - | - | - | - | - | - | - | - | - | - | - | - | - | - | 1 | 0.05 |
| TCACAAG | 1 | - | - | - | - | - | - | - | - | - | - | - | - | - | - | - | - | - | 1 | 0.05 |
| TCACCGG | 1 | - | - | - | - | - | - | - | - | - | - | - | - | - | - | - | - | - | 1 | 0.05 |
| TCAGCTC | 1 | - | - | - | - | - | - | - | - | - | - | - | - | - | - | - | - | - | 1 | 0.05 |
| TCATCTC | 1 | - | - | - | - | - | - | - | - | - | - | - | - | - | - | - | - | - | 1 | 0.05 |
| TCATGGC | 1 | - | - | - | - | - | - | - | - | - | - | - | - | - | - | - | - | - | 1 | 0.05 |
| TCCATGG | 1 | - | - | - | - | - | - | - | - | - | - | - | - | - | - | - | - | - | 1 | 0.05 |
| TCCCACA | 1 | - | - | - | - | - | - | - | - | - | - | - | - | - | - | - | - | - | 1 | 0.05 |
| TCCCAGC | 1 | - | - | - | - | - | - | - | - | - | - | - | - | - | - | - | - | - | 1 | 0.05 |
| TCCCTCA | 1 | - | - | - | - | - | - | - | - | - | - | - | - | - | - | - | - | - | 1 | 0.05 |
| TCCTCTC | 1 | - | - | - | - | - | - | - | - | - | - | - | - | - | - | - | - | - | 1 | 0.05 |
| TCCTTAT | 1 | - | - | - | - | - | - | - | - | - | - | - | - | - | - | - | - | - | 1 | 0.05 |
| TCCTTCC | 1 | - | - | - | - | - | - | - | - | - | - | - | - | - | - | - | - | - | 1 | 0.05 |
| TCCTTGG | 1 | - | - | - | - | - | - | - | - | - | - | - | - | - | - | - | - | - | 1 | 0.05 |
| TCGAACT | 1 | - | - | - | - | - | - | - | - | - | - | - | - | - | - | - | - | - | 1 | 0.05 |
| TCGGCCT | 1 | - | - | - | - | - | - | - | - | - | - | - | - | - | - | - | - | - | 1 | 0.05 |
| TCTAAGT | 1 | - | - | - | - | - | - | - | - | - | - | - | - | - | - | - | - | - | 1 | 0.05 |
| TCTAATG | 1 | - | - | - | - | - | - | - | - | - | - | - | - | - | - | - | - | - | 1 | 0.05 |
| TCTAGCA | 1 | - | - | - | - | - | - | - | - | - | - | - | - | - | - | - | - | - | 1 | 0.05 |
| TCTATTG | 1 | - | - | - | - | - | - | - | - | - | - | - | - | - | - | - | - | - | 1 | 0.05 |
| TCTCGCC | 1 | - | - | - | - | - | - | - | - | - | - | - | - | - | - | - | - | - | 1 | 0.05 |
| TCTCTCC | 1 | - | - | - | - | - | - | - | - | - | - | - | - | - | - | - | - | - | 1 | 0.05 |
| TCTCTTC | 1 | - | - | - | - | - | - | - | - | - | - | - | - | - | - | - | - | - | 1 | 0.05 |
| TCTGAAC | 1 | - | - | - | - | - | - | - | - | - | - | - | - | - | - | - | - | - | 1 | 0.05 |
| TCTGCTG | 1 | - | - | - | - | - | - | - | - | - | - | - | - | - | - | - | - | - | 1 | 0.05 |
| TCTGTTT | 1 | - | - | - | - | - | - | - | - | - | - | - | - | - | - | - | - | - | 1 | 0.05 |
| TCTTATG | 1 | - | - | - | - | - | - | - | - | - | - | - | - | - | - | - | - | - | 1 | 0.05 |
| TCTTCAT | 1 | - | - | - | - | - | - | - | - | - | - | - | - | - | - | - | - | - | 1 | 0.05 |
| TCTTCTC | 1 | - | - | - | - | - | - | - | - | - | - | - | - | - | - | - | - | - | 1 | 0.05 |
| TCTTCTT | 1 | - | - | - | - | - | - | - | - | - | - | - | - | - | - | - | - | - | 1 | 0.05 |
| TCTTGGC | 1 | - | - | - | - | - | - | - | - | - | - | - | - | - | - | - | - | - | 1 | 0.05 |
| TCTTTTA | 1 | - | - | - | - | - | - | - | - | - | - | - | - | - | - | - | - | - | 1 | 0.05 |
| TGAAACA | 1 | - | - | - | - | - | - | - | - | - | - | - | - | - | - | - | - | - | 1 | 0.05 |
| TGAAGCA | 1 | - | - | - | - | - | - | - | - | - | - | - | - | - | - | - | - | - | 1 | 0.05 |
| TGAATCT | 1 | - | - | - | - | - | - | - | - | - | - | - | - | - | - | - | - | - | 1 | 0.05 |
| TGACAAC | 1 | - | - | - | - | - | - | - | - | - | - | - | - | - | - | - | - | - | 1 | 0.05 |
| TGAGAAC | 1 | - | - | - | - | - | - | - | - | - | - | - | - | - | - | - | - | - | 1 | 0.05 |
| TGAGAGA | 1 | - | - | - | - | - | - | - | - | - | - | - | - | - | - | - | - | - | 1 | 0.05 |
| TGAGGAT | 1 | - | - | - | - | - | - | - | - | - | - | - | - | - | - | - | - | - | 1 | 0.05 |
| TGCAATT | 1 | - | - | - | - | - | - | - | - | - | - | - | - | - | - | - | - | - | 1 | 0.05 |
| TGCTTCT | 1 | - | - | - | - | - | - | - | - | - | - | - | - | - | - | - | - | - | 1 | 0.05 |
| TGCTTGA | 1 | - | - | - | - | - | - | - | - | - | - | - | - | - | - | - | - | - | 1 | 0.05 |
| TGGCATG | 1 | - | - | - | - | - | - | - | - | - | - | - | - | - | - | - | - | - | 1 | 0.05 |
| TGGCTGA | 1 | - | - | - | - | - | - | - | - | - | - | - | - | - | - | - | - | - | 1 | 0.05 |
| TGGGAAA | 1 | - | - | - | - | - | - | - | - | - | - | - | - | - | - | - | - | - | 1 | 0.05 |
| TGGTATA | 1 | - | - | - | - | - | - | - | - | - | - | - | - | - | - | - | - | - | 1 | 0.05 |
| TGGTGCA | 1 | - | - | - | - | - | - | - | - | - | - | - | - | - | - | - | - | - | 1 | 0.05 |
| TGGTTTC | 1 | - | - | - | - | - | - | - | - | - | - | - | - | - | - | - | - | - | 1 | 0.05 |
| TGTATTT | 1 | - | - | - | - | - | - | - | - | - | - | - | - | - | - | - | - | - | 1 | 0.05 |
| TGTCATC | 1 | - | - | - | - | - | - | - | - | - | - | - | - | - | - | - | - | - | 1 | 0.05 |
| TGTCATT | 1 | - | - | - | - | - | - | - | - | - | - | - | - | - | - | - | - | - | 1 | 0.05 |
| TGTTATA | 1 | - | - | - | - | - | - | - | - | - | - | - | - | - | - | - | - | - | 1 | 0.05 |
| TGTTGCC | 1 | - | - | - | - | - | - | - | - | - | - | - | - | - | - | - | - | - | 1 | 0.05 |
| TGTTTGA | 1 | - | - | - | - | - | - | - | - | - | - | - | - | - | - | - | - | - | 1 | 0.05 |
| TTAAAAA | 1 | - | - | - | - | - | - | - | - | - | - | - | - | - | - | - | - | - | 1 | 0.05 |
| TTAAACC | 1 | - | - | - | - | - | - | - | - | - | - | - | - | - | - | - | - | - | 1 | 0.05 |
| TTAATAT | 1 | - | - | - | - | - | - | - | - | - | - | - | - | - | - | - | - | - | 1 | 0.05 |
| TTACAAA | 1 | - | - | - | - | - | - | - | - | - | - | - | - | - | - | - | - | - | 1 | 0.05 |
| TTACCAG | 1 | - | - | - | - | - | - | - | - | - | - | - | - | - | - | - | - | - | 1 | 0.05 |
| TTACTCT | 1 | - | - | - | - | - | - | - | - | - | - | - | - | - | - | - | - | - | 1 | 0.05 |
| TTAGCCT | 1 | - | - | - | - | - | - | - | - | - | - | - | - | - | - | - | - | - | 1 | 0.05 |
| TTAGTTA | 1 | - | - | - | - | - | - | - | - | - | - | - | - | - | - | - | - | - | 1 | 0.05 |
| TTCAGTG | 1 | - | - | - | - | - | - | - | - | - | - | - | - | - | - | - | - | - | 1 | 0.05 |
| TTCATAT | 1 | - | - | - | - | - | - | - | - | - | - | - | - | - | - | - | - | - | 1 | 0.05 |
| TTCCAGA | 1 | - | - | - | - | - | - | - | - | - | - | - | - | - | - | - | - | - | 1 | 0.05 |
| TTCCATA | 1 | - | - | - | - | - | - | - | - | - | - | - | - | - | - | - | - | - | 1 | 0.05 |
| TTCCATG | 1 | - | - | - | - | - | - | - | - | - | - | - | - | - | - | - | - | - | 1 | 0.05 |
| TTCCCAA | 1 | - | - | - | - | - | - | - | - | - | - | - | - | - | - | - | - | - | 1 | 0.05 |
| TTCCTTC | 1 | - | - | - | - | - | - | - | - | - | - | - | - | - | - | - | - | - | 1 | 0.05 |
| TTCCTTT | 1 | - | - | - | - | - | - | - | - | - | - | - | - | - | - | - | - | - | 1 | 0.05 |
| TTCTCTT | 1 | - | - | - | - | - | - | - | - | - | - | - | - | - | - | - | - | - | 1 | 0.05 |
| TTCTTTC | 1 | - | - | - | - | - | - | - | - | - | - | - | - | - | - | - | - | - | 1 | 0.05 |
| TTCTTTG | 1 | - | - | - | - | - | - | - | - | - | - | - | - | - | - | - | - | - | 1 | 0.05 |
| TTGAAAT | 1 | - | - | - | - | - | - | - | - | - | - | - | - | - | - | - | - | - | 1 | 0.05 |
| TTGATAC | 1 | - | - | - | - | - | - | - | - | - | - | - | - | - | - | - | - | - | 1 | 0.05 |
| TTGATGA | 1 | - | - | - | - | - | - | - | - | - | - | - | - | - | - | - | - | - | 1 | 0.05 |
| TTGATTT | 1 | - | - | - | - | - | - | - | - | - | - | - | - | - | - | - | - | - | 1 | 0.05 |
| TTGCAAT | 1 | - | - | - | - | - | - | - | - | - | - | - | - | - | - | - | - | - | 1 | 0.05 |
| TTGCAGC | 1 | - | - | - | - | - | - | - | - | - | - | - | - | - | - | - | - | - | 1 | 0.05 |
| TTGCATG | 1 | - | - | - | - | - | - | - | - | - | - | - | - | - | - | - | - | - | 1 | 0.05 |
| TTGCATT | 1 | - | - | - | - | - | - | - | - | - | - | - | - | - | - | - | - | - | 1 | 0.05 |
| TTGCCCA | - | 1 | - | - | - | - | - | - | - | - | - | - | - | - | - | - | - | - | 1 | 0.05 |
| TTGCCTC | 1 | - | - | - | - | - | - | - | - | - | - | - | - | - | - | - | - | - | 1 | 0.05 |
| TTGCTGC | 1 | - | - | - | - | - | - | - | - | - | - | - | - | - | - | - | - | - | 1 | 0.05 |
| TTGGAAG | 1 | - | - | - | - | - | - | - | - | - | - | - | - | - | - | - | - | - | 1 | 0.05 |
| TTGGCGG | 1 | - | - | - | - | - | - | - | - | - | - | - | - | - | - | - | - | - | 1 | 0.05 |
| TTGGCTG | 1 | - | - | - | - | - | - | - | - | - | - | - | - | - | - | - | - | - | 1 | 0.05 |
| TTGTAAC | 1 | - | - | - | - | - | - | - | - | - | - | - | - | - | - | - | - | - | 1 | 0.05 |
| TTGTTGC | 1 | - | - | - | - | - | - | - | - | - | - | - | - | - | - | - | - | - | 1 | 0.05 |
| TTGTTTT | 1 | - | - | - | - | - | - | - | - | - | - | - | - | - | - | - | - | - | 1 | 0.05 |
| TTTAATC | 1 | - | - | - | - | - | - | - | - | - | - | - | - | - | - | - | - | - | 1 | 0.05 |
| TTTACAT | 1 | - | - | - | - | - | - | - | - | - | - | - | - | - | - | - | - | - | 1 | 0.05 |
| TTTATAG | 1 | - | - | - | - | - | - | - | - | - | - | - | - | - | - | - | - | - | 1 | 0.05 |
| TTTATCA | 1 | - | - | - | - | - | - | - | - | - | - | - | - | - | - | - | - | - | 1 | 0.05 |
| TTTATCC | 1 | - | - | - | - | - | - | - | - | - | - | - | - | - | - | - | - | - | 1 | 0.05 |
| TTTATTT | 1 | - | - | - | - | - | - | - | - | - | - | - | - | - | - | - | - | - | 1 | 0.05 |
| TTTCACC | 1 | - | - | - | - | - | - | - | - | - | - | - | - | - | - | - | - | - | 1 | 0.05 |
| TTTCTAG | 1 | - | - | - | - | - | - | - | - | - | - | - | - | - | - | - | - | - | 1 | 0.05 |
| TTTCTAT | 1 | - | - | - | - | - | - | - | - | - | - | - | - | - | - | - | - | - | 1 | 0.05 |
| TTTCTGG | 1 | - | - | - | - | - | - | - | - | - | - | - | - | - | - | - | - | - | 1 | 0.05 |
| TTTCTTC | 1 | - | - | - | - | - | - | - | - | - | - | - | - | - | - | - | - | - | 1 | 0.05 |
| TTTCTTG | 1 | - | - | - | - | - | - | - | - | - | - | - | - | - | - | - | - | - | 1 | 0.05 |
| TTTGAAA | 1 | - | - | - | - | - | - | - | - | - | - | - | - | - | - | - | - | - | 1 | 0.05 |
| TTTGAAC | 1 | - | - | - | - | - | - | - | - | - | - | - | - | - | - | - | - | - | 1 | 0.05 |
| TTTGGGT | 1 | - | - | - | - | - | - | - | - | - | - | - | - | - | - | - | - | - | 1 | 0.05 |
| TTTGGTT | 1 | - | - | - | - | - | - | - | - | - | - | - | - | - | - | - | - | - | 1 | 0.05 |
| TTTTATC | 1 | - | - | - | - | - | - | - | - | - | - | - | - | - | - | - | - | - | 1 | 0.05 |
| TTTTATT | 1 | - | - | - | - | - | - | - | - | - | - | - | - | - | - | - | - | - | 1 | 0.05 |
| TTTTCAA | 1 | - | - | - | - | - | - | - | - | - | - | - | - | - | - | - | - | - | 1 | 0.05 |
| TTTTCAC | 1 | - | - | - | - | - | - | - | - | - | - | - | - | - | - | - | - | - | 1 | 0.05 |
| TTTTCTA | 1 | - | - | - | - | - | - | - | - | - | - | - | - | - | - | - | - | - | 1 | 0.05 |
| TTTTCTC | 1 | - | - | - | - | - | - | - | - | - | - | - | - | - | - | - | - | - | 1 | 0.05 |
| TTTTCTG | 1 | - | - | - | - | - | - | - | - | - | - | - | - | - | - | - | - | - | 1 | 0.05 |
| TTTTCTT | 1 | - | - | - | - | - | - | - | - | - | - | - | - | - | - | - | - | - | 1 | 0.05 |
| TTTTGAG | 1 | - | - | - | - | - | - | - | - | - | - | - | - | - | - | - | - | - | 1 | 0.05 |
| TTTTGGA | 1 | - | - | - | - | - | - | - | - | - | - | - | - | - | - | - | - | - | 1 | 0.05 |
| TTTTGTT | 1 | - | - | - | - | - | - | - | - | - | - | - | - | - | - | - | - | - | 1 | 0.05 |
| TTTTTAA | 1 | - | - | - | - | - | - | - | - | - | - | - | - | - | - | - | - | - | 1 | 0.05 |
| TTTTTAT | 1 | - | - | - | - | - | - | - | - | - | - | - | - | - | - | - | - | - | 1 | 0.05 |
| TTTTTCC | 1 | - | - | - | - | - | - | - | - | - | - | - | - | - | - | - | - | - | 1 | 0.05 |
| TTTTTGC | 1 | - | - | - | - | - | - | - | - | - | - | - | - | - | - | - | - | - | 1 | 0.05 |
| TTTTTGG | 1 | - | - | - | - | - | - | - | - | - | - | - | - | - | - | - | - | - | 1 | 0.05 |
| TTTTTGT | - | 1 | - | - | - | - | - | - | - | - | - | - | - | - | - | - | - | - | 1 | 0.05 |
| TTTTTTA | 1 | - | - | - | - | - | - | - | - | - | - | - | - | - | - | - | - | - | 1 | 0.05 |
| TTTTTTTA | 3 | - | - | - | - | - | - | - | - | - | - | - | - | - | - | - | - | - | 3 | 0.16 |
| AAAAAAAT | 2 | - | - | - | - | - | - | - | - | - | - | - | - | - | - | - | - | - | 2 | 0.11 |
| AAAGAGAG | 2 | - | - | - | - | - | - | - | - | - | - | - | - | - | - | - | - | - | 2 | 0.11 |
| AATAAATC | 2 | - | - | - | - | - | - | - | - | - | - | - | - | - | - | - | - | - | 2 | 0.11 |
| ATCAACTC | 2 | - | - | - | - | - | - | - | - | - | - | - | - | - | - | - | - | - | 2 | 0.11 |
| ATGAAGTT | 2 | - | - | - | - | - | - | - | - | - | - | - | - | - | - | - | - | - | 2 | 0.11 |
| GCTGTATT | 2 | - | - | - | - | - | - | - | - | - | - | - | - | - | - | - | - | - | 2 | 0.11 |
| TAGCAACA | 2 | - | - | - | - | - | - | - | - | - | - | - | - | - | - | - | - | - | 2 | 0.11 |
| TTTTCTTC | 2 | - | - | - | - | - | - | - | - | - | - | - | - | - | - | - | - | - | 2 | 0.11 |
| AAAAAAGT | 1 | - | - | - | - | - | - | - | - | - | - | - | - | - | - | - | - | - | 1 | 0.05 |
| AAAATAAA | 1 | - | - | - | - | - | - | - | - | - | - | - | - | - | - | - | - | - | 1 | 0.05 |
| AAACCCCC | 1 | - | - | - | - | - | - | - | - | - | - | - | - | - | - | - | - | - | 1 | 0.05 |
| AAAGAAAA | 1 | - | - | - | - | - | - | - | - | - | - | - | - | - | - | - | - | - | 1 | 0.05 |
| AAAGCCAC | 1 | - | - | - | - | - | - | - | - | - | - | - | - | - | - | - | - | - | 1 | 0.05 |
| AAAGTTCC | 1 | - | - | - | - | - | - | - | - | - | - | - | - | - | - | - | - | - | 1 | 0.05 |
| AAATGGAA | 1 | - | - | - | - | - | - | - | - | - | - | - | - | - | - | - | - | - | 1 | 0.05 |
| AAATTGAC | 1 | - | - | - | - | - | - | - | - | - | - | - | - | - | - | - | - | - | 1 | 0.05 |
| AAATTTTG | 1 | - | - | - | - | - | - | - | - | - | - | - | - | - | - | - | - | - | 1 | 0.05 |
| AACAAAAA | 1 | - | - | - | - | - | - | - | - | - | - | - | - | - | - | - | - | - | 1 | 0.05 |
| AACCCTGA | 1 | - | - | - | - | - | - | - | - | - | - | - | - | - | - | - | - | - | 1 | 0.05 |
| AAGAAAAA | 1 | - | - | - | - | - | - | - | - | - | - | - | - | - | - | - | - | - | 1 | 0.05 |
| AAGAAACA | 1 | - | - | - | - | - | - | - | - | - | - | - | - | - | - | - | - | - | 1 | 0.05 |
| AAGAAGAA | 1 | - | - | - | - | - | - | - | - | - | - | - | - | - | - | - | - | - | 1 | 0.05 |
| AAGACAAA | 1 | - | - | - | - | - | - | - | - | - | - | - | - | - | - | - | - | - | 1 | 0.05 |
| AAGAGCAA | 1 | - | - | - | - | - | - | - | - | - | - | - | - | - | - | - | - | - | 1 | 0.05 |
| AAGCTCTT | 1 | - | - | - | - | - | - | - | - | - | - | - | - | - | - | - | - | - | 1 | 0.05 |
| AAGGCAAT | 1 | - | - | - | - | - | - | - | - | - | - | - | - | - | - | - | - | - | 1 | 0.05 |
| AAGTATGG | 1 | - | - | - | - | - | - | - | - | - | - | - | - | - | - | - | - | - | 1 | 0.05 |
| AATGCACC | 1 | - | - | - | - | - | - | - | - | - | - | - | - | - | - | - | - | - | 1 | 0.05 |
| AATGCATC | 1 | - | - | - | - | - | - | - | - | - | - | - | - | - | - | - | - | - | 1 | 0.05 |
| AATGGGAG | 1 | - | - | - | - | - | - | - | - | - | - | - | - | - | - | - | - | - | 1 | 0.05 |
| AATTATTT | 1 | - | - | - | - | - | - | - | - | - | - | - | - | - | - | - | - | - | 1 | 0.05 |
| AATTCCAT | 1 | - | - | - | - | - | - | - | - | - | - | - | - | - | - | - | - | - | 1 | 0.05 |
| AATTTTTT | 1 | - | - | - | - | - | - | - | - | - | - | - | - | - | - | - | - | - | 1 | 0.05 |
| ACAAAAAC | 1 | - | - | - | - | - | - | - | - | - | - | - | - | - | - | - | - | - | 1 | 0.05 |
| ACACACTC | 1 | - | - | - | - | - | - | - | - | - | - | - | - | - | - | - | - | - | 1 | 0.05 |
| ACAGCTGC | 1 | - | - | - | - | - | - | - | - | - | - | - | - | - | - | - | - | - | 1 | 0.05 |
| ACCTTTTA | 1 | - | - | - | - | - | - | - | - | - | - | - | - | - | - | - | - | - | 1 | 0.05 |
| ACTCCTTG | 1 | - | - | - | - | - | - | - | - | - | - | - | - | - | - | - | - | - | 1 | 0.05 |
| ACTTTTTC | 1 | - | - | - | - | - | - | - | - | - | - | - | - | - | - | - | - | - | 1 | 0.05 |
| AGAAGGAG | 1 | - | - | - | - | - | - | - | - | - | - | - | - | - | - | - | - | - | 1 | 0.05 |
| AGAATCAG | 1 | - | - | - | - | - | - | - | - | - | - | - | - | - | - | - | - | - | 1 | 0.05 |
| AGAGAATC | 1 | - | - | - | - | - | - | - | - | - | - | - | - | - | - | - | - | - | 1 | 0.05 |
| AGAGAGAA | 1 | - | - | - | - | - | - | - | - | - | - | - | - | - | - | - | - | - | 1 | 0.05 |
| AGCCTCTC | 1 | - | - | - | - | - | - | - | - | - | - | - | - | - | - | - | - | - | 1 | 0.05 |
| AGTTTATG | 1 | - | - | - | - | - | - | - | - | - | - | - | - | - | - | - | - | - | 1 | 0.05 |
| ATAAAACC | 1 | - | - | - | - | - | - | - | - | - | - | - | - | - | - | - | - | - | 1 | 0.05 |
| ATAGAGAT | 1 | - | - | - | - | - | - | - | - | - | - | - | - | - | - | - | - | - | 1 | 0.05 |
| ATATAATA | 1 | - | - | - | - | - | - | - | - | - | - | - | - | - | - | - | - | - | 1 | 0.05 |
| ATGCATTG | 1 | - | - | - | - | - | - | - | - | - | - | - | - | - | - | - | - | - | 1 | 0.05 |
| ATGGCTGA | 1 | - | - | - | - | - | - | - | - | - | - | - | - | - | - | - | - | - | 1 | 0.05 |
| ATTCACGA | 1 | - | - | - | - | - | - | - | - | - | - | - | - | - | - | - | - | - | 1 | 0.05 |
| ATTCGATG | 1 | - | - | - | - | - | - | - | - | - | - | - | - | - | - | - | - | - | 1 | 0.05 |
| ATTGTGTA | 1 | - | - | - | - | - | - | - | - | - | - | - | - | - | - | - | - | - | 1 | 0.05 |
| CAAGCCAT | 1 | - | - | - | - | - | - | - | - | - | - | - | - | - | - | - | - | - | 1 | 0.05 |
| CAATGCCT | 1 | - | - | - | - | - | - | - | - | - | - | - | - | - | - | - | - | - | 1 | 0.05 |
| CAGACTTA | 1 | - | - | - | - | - | - | - | - | - | - | - | - | - | - | - | - | - | 1 | 0.05 |
| CAGAGGAC | 1 | - | - | - | - | - | - | - | - | - | - | - | - | - | - | - | - | - | 1 | 0.05 |
| CAGTAATT | 1 | - | - | - | - | - | - | - | - | - | - | - | - | - | - | - | - | - | 1 | 0.05 |
| CATCAACT | 1 | - | - | - | - | - | - | - | - | - | - | - | - | - | - | - | - | - | 1 | 0.05 |
| CATTTCTA | 1 | - | - | - | - | - | - | - | - | - | - | - | - | - | - | - | - | - | 1 | 0.05 |
| CCAAAAAA | 1 | - | - | - | - | - | - | - | - | - | - | - | - | - | - | - | - | - | 1 | 0.05 |
| CCAAACAA | 1 | - | - | - | - | - | - | - | - | - | - | - | - | - | - | - | - | - | 1 | 0.05 |
| CCCACCAG | 1 | - | - | - | - | - | - | - | - | - | - | - | - | - | - | - | - | - | 1 | 0.05 |
| CCCCGGGA | 1 | - | - | - | - | - | - | - | - | - | - | - | - | - | - | - | - | - | 1 | 0.05 |
| CCTAAATC | 1 | - | - | - | - | - | - | - | - | - | - | - | - | - | - | - | - | - | 1 | 0.05 |
| CCTCCTCG | 1 | - | - | - | - | - | - | - | - | - | - | - | - | - | - | - | - | - | 1 | 0.05 |
| CCTGTTGA | 1 | - | - | - | - | - | - | - | - | - | - | - | - | - | - | - | - | - | 1 | 0.05 |
| CGAGGAGG | 1 | - | - | - | - | - | - | - | - | - | - | - | - | - | - | - | - | - | 1 | 0.05 |
| CTCTTCTC | 1 | - | - | - | - | - | - | - | - | - | - | - | - | - | - | - | - | - | 1 | 0.05 |
| CTTCTCTT | 1 | - | - | - | - | - | - | - | - | - | - | - | - | - | - | - | - | - | 1 | 0.05 |
| CTTCTTCT | 1 | - | - | - | - | - | - | - | - | - | - | - | - | - | - | - | - | - | 1 | 0.05 |
| CTTGAACA | 1 | - | - | - | - | - | - | - | - | - | - | - | - | - | - | - | - | - | 1 | 0.05 |
| CTTGATTT | 1 | - | - | - | - | - | - | - | - | - | - | - | - | - | - | - | - | - | 1 | 0.05 |
| CTTTCTGT | 1 | - | - | - | - | - | - | - | - | - | - | - | - | - | - | - | - | - | 1 | 0.05 |
| GAAACTTG | 1 | - | - | - | - | - | - | - | - | - | - | - | - | - | - | - | - | - | 1 | 0.05 |
| GAAAGATT | 1 | - | - | - | - | - | - | - | - | - | - | - | - | - | - | - | - | - | 1 | 0.05 |
| GAAATAAT | 1 | - | - | - | - | - | - | - | - | - | - | - | - | - | - | - | - | - | 1 | 0.05 |
| GAACAAAC | 1 | - | - | - | - | - | - | - | - | - | - | - | - | - | - | - | - | - | 1 | 0.05 |
| GAAGATTG | 1 | - | - | - | - | - | - | - | - | - | - | - | - | - | - | - | - | - | 1 | 0.05 |
| GAAGCAAA | 1 | - | - | - | - | - | - | - | - | - | - | - | - | - | - | - | - | - | 1 | 0.05 |
| GACAAAAA | 1 | - | - | - | - | - | - | - | - | - | - | - | - | - | - | - | - | - | 1 | 0.05 |
| GACAGAGA | 1 | - | - | - | - | - | - | - | - | - | - | - | - | - | - | - | - | - | 1 | 0.05 |
| GACATTCT | 1 | - | - | - | - | - | - | - | - | - | - | - | - | - | - | - | - | - | 1 | 0.05 |
| GAGACTGA | 1 | - | - | - | - | - | - | - | - | - | - | - | - | - | - | - | - | - | 1 | 0.05 |
| GAGAGATC | 1 | - | - | - | - | - | - | - | - | - | - | - | - | - | - | - | - | - | 1 | 0.05 |
| GAGATTGA | 1 | - | - | - | - | - | - | - | - | - | - | - | - | - | - | - | - | - | 1 | 0.05 |
| GAGCCTCA | 1 | - | - | - | - | - | - | - | - | - | - | - | - | - | - | - | - | - | 1 | 0.05 |
| GCAATTTT | 1 | - | - | - | - | - | - | - | - | - | - | - | - | - | - | - | - | - | 1 | 0.05 |
| GCAGGAGA | 1 | - | - | - | - | - | - | - | - | - | - | - | - | - | - | - | - | - | 1 | 0.05 |
| GCCAACTT | 1 | - | - | - | - | - | - | - | - | - | - | - | - | - | - | - | - | - | 1 | 0.05 |
| GCCTTTCT | 1 | - | - | - | - | - | - | - | - | - | - | - | - | - | - | - | - | - | 1 | 0.05 |
| GGAAGGCT | 1 | - | - | - | - | - | - | - | - | - | - | - | - | - | - | - | - | - | 1 | 0.05 |
| GGAGATGA | 1 | - | - | - | - | - | - | - | - | - | - | - | - | - | - | - | - | - | 1 | 0.05 |
| GGCGCCCC | 1 | - | - | - | - | - | - | - | - | - | - | - | - | - | - | - | - | - | 1 | 0.05 |
| GGGGAGAG | 1 | - | - | - | - | - | - | - | - | - | - | - | - | - | - | - | - | - | 1 | 0.05 |
| GGTTGGAT | 1 | - | - | - | - | - | - | - | - | - | - | - | - | - | - | - | - | - | 1 | 0.05 |
| GTCTTCTT | 1 | - | - | - | - | - | - | - | - | - | - | - | - | - | - | - | - | - | 1 | 0.05 |
| GTTCGAAA | 1 | - | - | - | - | - | - | - | - | - | - | - | - | - | - | - | - | - | 1 | 0.05 |
| GTTTATGC | 1 | - | - | - | - | - | - | - | - | - | - | - | - | - | - | - | - | - | 1 | 0.05 |
| TAATAATA | 1 | - | - | - | - | - | - | - | - | - | - | - | - | - | - | - | - | - | 1 | 0.05 |
| TAATCAAT | 1 | - | - | - | - | - | - | - | - | - | - | - | - | - | - | - | - | - | 1 | 0.05 |
| TAATTCAT | 1 | - | - | - | - | - | - | - | - | - | - | - | - | - | - | - | - | - | 1 | 0.05 |
| TATAGCTA | 1 | - | - | - | - | - | - | - | - | - | - | - | - | - | - | - | - | - | 1 | 0.05 |
| TATATATT | 1 | - | - | - | - | - | - | - | - | - | - | - | - | - | - | - | - | - | 1 | 0.05 |
| TATTTTTT | 1 | - | - | - | - | - | - | - | - | - | - | - | - | - | - | - | - | - | 1 | 0.05 |
| TCAATCTC | 1 | - | - | - | - | - | - | - | - | - | - | - | - | - | - | - | - | - | 1 | 0.05 |
| TCATCTCC | 1 | - | - | - | - | - | - | - | - | - | - | - | - | - | - | - | - | - | 1 | 0.05 |
| TCCAATTT | 1 | - | - | - | - | - | - | - | - | - | - | - | - | - | - | - | - | - | 1 | 0.05 |
| TCTCTCAC | 1 | - | - | - | - | - | - | - | - | - | - | - | - | - | - | - | - | - | 1 | 0.05 |
| TCTTAGAA | 1 | - | - | - | - | - | - | - | - | - | - | - | - | - | - | - | - | - | 1 | 0.05 |
| TCTTATTA | 1 | - | - | - | - | - | - | - | - | - | - | - | - | - | - | - | - | - | 1 | 0.05 |
| TCTTCCTC | 1 | - | - | - | - | - | - | - | - | - | - | - | - | - | - | - | - | - | 1 | 0.05 |
| TCTTTCTG | 1 | - | - | - | - | - | - | - | - | - | - | - | - | - | - | - | - | - | 1 | 0.05 |
| TGAAAAGA | 1 | - | - | - | - | - | - | - | - | - | - | - | - | - | - | - | - | - | 1 | 0.05 |
| TGAAATAG | 1 | - | - | - | - | - | - | - | - | - | - | - | - | - | - | - | - | - | 1 | 0.05 |
| TGAGAGCT | 1 | - | - | - | - | - | - | - | - | - | - | - | - | - | - | - | - | - | 1 | 0.05 |
| TGATTTGT | 1 | - | - | - | - | - | - | - | - | - | - | - | - | - | - | - | - | - | 1 | 0.05 |
| TGCAATCG | 1 | - | - | - | - | - | - | - | - | - | - | - | - | - | - | - | - | - | 1 | 0.05 |
| TGCTACCG | 1 | - | - | - | - | - | - | - | - | - | - | - | - | - | - | - | - | - | 1 | 0.05 |
| TGGGGGCC | 1 | - | - | - | - | - | - | - | - | - | - | - | - | - | - | - | - | - | 1 | 0.05 |
| TTAACGAC | 1 | - | - | - | - | - | - | - | - | - | - | - | - | - | - | - | - | - | 1 | 0.05 |
| TTAATTAT | 1 | - | - | - | - | - | - | - | - | - | - | - | - | - | - | - | - | - | 1 | 0.05 |
| TTACAATA | 1 | - | - | - | - | - | - | - | - | - | - | - | - | - | - | - | - | - | 1 | 0.05 |
| TTATTGTA | 1 | - | - | - | - | - | - | - | - | - | - | - | - | - | - | - | - | - | 1 | 0.05 |
| TTATTTTA | 1 | - | - | - | - | - | - | - | - | - | - | - | - | - | - | - | - | - | 1 | 0.05 |
| TTCACTAA | 1 | - | - | - | - | - | - | - | - | - | - | - | - | - | - | - | - | - | 1 | 0.05 |
| TTCAGAAG | 1 | - | - | - | - | - | - | - | - | - | - | - | - | - | - | - | - | - | 1 | 0.05 |
| TTCATAAT | 1 | - | - | - | - | - | - | - | - | - | - | - | - | - | - | - | - | - | 1 | 0.05 |
| TTCTCTCT | 1 | - | - | - | - | - | - | - | - | - | - | - | - | - | - | - | - | - | 1 | 0.05 |
| TTCTCTTC | 1 | - | - | - | - | - | - | - | - | - | - | - | - | - | - | - | - | - | 1 | 0.05 |
| TTCTGGCA | 1 | - | - | - | - | - | - | - | - | - | - | - | - | - | - | - | - | - | 1 | 0.05 |
| TTCTTCTT | 1 | - | - | - | - | - | - | - | - | - | - | - | - | - | - | - | - | - | 1 | 0.05 |
| TTCTTTTC | 1 | - | - | - | - | - | - | - | - | - | - | - | - | - | - | - | - | - | 1 | 0.05 |
| TTCTTTTT | 1 | - | - | - | - | - | - | - | - | - | - | - | - | - | - | - | - | - | 1 | 0.05 |
| TTTATCTT | 1 | - | - | - | - | - | - | - | - | - | - | - | - | - | - | - | - | - | 1 | 0.05 |
| TTTATTAT | 1 | - | - | - | - | - | - | - | - | - | - | - | - | - | - | - | - | - | 1 | 0.05 |
| TTTCAATC | 1 | - | - | - | - | - | - | - | - | - | - | - | - | - | - | - | - | - | 1 | 0.05 |
| TTTCCCCT | 1 | - | - | - | - | - | - | - | - | - | - | - | - | - | - | - | - | - | 1 | 0.05 |
| TTTCTTCA | 1 | - | - | - | - | - | - | - | - | - | - | - | - | - | - | - | - | - | 1 | 0.05 |
| TTTGGAGG | 1 | - | - | - | - | - | - | - | - | - | - | - | - | - | - | - | - | - | 1 | 0.05 |
| TTTTAGAG | 1 | - | - | - | - | - | - | - | - | - | - | - | - | - | - | - | - | - | 1 | 0.05 |
| TTTTGTTT | 1 | - | - | - | - | - | - | - | - | - | - | - | - | - | - | - | - | - | 1 | 0.05 |
| TTTTTGTG | 1 | - | - | - | - | - | - | - | - | - | - | - | - | - | - | - | - | - | 1 | 0.05 |
| TTTTTTAT | 1 | - | - | - | - | - | - | - | - | - | - | - | - | - | - | - | - | - | 1 | 0.05 |
| TCTTCCTCT | 2 | 1 | - | - | - | - | - | - | - | - | - | - | - | - | - | - | - | - | 3 | 0.16 |
| CGTACTATT | 2 | - | - | - | - | - | - | - | - | - | - | - | - | - | - | - | - | - | 2 | 0.11 |
| TGAAGAGGA | 2 | - | - | - | - | - | - | - | - | - | - | - | - | - | - | - | - | - | 2 | 0.11 |
| TTCTCCTTC | 2 | - | - | - | - | - | - | - | - | - | - | - | - | - | - | - | - | - | 2 | 0.11 |
| AAAAAATAA | 1 | - | - | - | - | - | - | - | - | - | - | - | - | - | - | - | - | - | 1 | 0.05 |
| AAAAGAAAA | 1 | - | - | - | - | - | - | - | - | - | - | - | - | - | - | - | - | - | 1 | 0.05 |
| AAAGGAAAA | 1 | - | - | - | - | - | - | - | - | - | - | - | - | - | - | - | - | - | 1 | 0.05 |
| AAATATACA | 1 | - | - | - | - | - | - | - | - | - | - | - | - | - | - | - | - | - | 1 | 0.05 |
| AAATTACAG | 1 | - | - | - | - | - | - | - | - | - | - | - | - | - | - | - | - | - | 1 | 0.05 |
| AACTTTCAG | 1 | - | - | - | - | - | - | - | - | - | - | - | - | - | - | - | - | - | 1 | 0.05 |
| AAGAAAGTT | 1 | - | - | - | - | - | - | - | - | - | - | - | - | - | - | - | - | - | 1 | 0.05 |
| AAGAATTGA | 1 | - | - | - | - | - | - | - | - | - | - | - | - | - | - | - | - | - | 1 | 0.05 |
| AAGAGGAAG | 1 | - | - | - | - | - | - | - | - | - | - | - | - | - | - | - | - | - | 1 | 0.05 |
| AAGATGGCA | 1 | - | - | - | - | - | - | - | - | - | - | - | - | - | - | - | - | - | 1 | 0.05 |
| AAGCAATAG | 1 | - | - | - | - | - | - | - | - | - | - | - | - | - | - | - | - | - | 1 | 0.05 |
| AAGCCACCA | 1 | - | - | - | - | - | - | - | - | - | - | - | - | - | - | - | - | - | 1 | 0.05 |
| AAGGAAAGG | 1 | - | - | - | - | - | - | - | - | - | - | - | - | - | - | - | - | - | 1 | 0.05 |
| AAGTGCAGC | 1 | - | - | - | - | - | - | - | - | - | - | - | - | - | - | - | - | - | 1 | 0.05 |
| ACACGAAAC | 1 | - | - | - | - | - | - | - | - | - | - | - | - | - | - | - | - | - | 1 | 0.05 |
| ACACGACAC | 1 | - | - | - | - | - | - | - | - | - | - | - | - | - | - | - | - | - | 1 | 0.05 |
| ACATCAAAA | 1 | - | - | - | - | - | - | - | - | - | - | - | - | - | - | - | - | - | 1 | 0.05 |
| ACATGAGAA | 1 | - | - | - | - | - | - | - | - | - | - | - | - | - | - | - | - | - | 1 | 0.05 |
| ACATGATTA | 1 | - | - | - | - | - | - | - | - | - | - | - | - | - | - | - | - | - | 1 | 0.05 |
| AGAAACATC | 1 | - | - | - | - | - | - | - | - | - | - | - | - | - | - | - | - | - | 1 | 0.05 |
| AGAAAGCAA | 1 | - | - | - | - | - | - | - | - | - | - | - | - | - | - | - | - | - | 1 | 0.05 |
| AGAAGCAGC | 1 | - | - | - | - | - | - | - | - | - | - | - | - | - | - | - | - | - | 1 | 0.05 |
| AGAGAGCTG | 1 | - | - | - | - | - | - | - | - | - | - | - | - | - | - | - | - | - | 1 | 0.05 |
| AGATGATGA | 1 | - | - | - | - | - | - | - | - | - | - | - | - | - | - | - | - | - | 1 | 0.05 |
| AGCCTGCTA | 1 | - | - | - | - | - | - | - | - | - | - | - | - | - | - | - | - | - | 1 | 0.05 |
| AGCTGTGCA | 1 | - | - | - | - | - | - | - | - | - | - | - | - | - | - | - | - | - | 1 | 0.05 |
| AGGAAGCTG | 1 | - | - | - | - | - | - | - | - | - | - | - | - | - | - | - | - | - | 1 | 0.05 |
| AGGACCTGG | 1 | - | - | - | - | - | - | - | - | - | - | - | - | - | - | - | - | - | 1 | 0.05 |
| AGGCAGCAA | 1 | - | - | - | - | - | - | - | - | - | - | - | - | - | - | - | - | - | 1 | 0.05 |
| AGGTGGAGG | 1 | - | - | - | - | - | - | - | - | - | - | - | - | - | - | - | - | - | 1 | 0.05 |
| AGTAATAAT | 1 | - | - | - | - | - | - | - | - | - | - | - | - | - | - | - | - | - | 1 | 0.05 |
| ATCAACTTC | 1 | - | - | - | - | - | - | - | - | - | - | - | - | - | - | - | - | - | 1 | 0.05 |
| ATCATAATC | 1 | - | - | - | - | - | - | - | - | - | - | - | - | - | - | - | - | - | 1 | 0.05 |
| ATCATCACT | 1 | - | - | - | - | - | - | - | - | - | - | - | - | - | - | - | - | - | 1 | 0.05 |
| ATCTTCTTC | 1 | - | - | - | - | - | - | - | - | - | - | - | - | - | - | - | - | - | 1 | 0.05 |
| ATGATTCGG | 1 | - | - | - | - | - | - | - | - | - | - | - | - | - | - | - | - | - | 1 | 0.05 |
| ATTAAAAAT | 1 | - | - | - | - | - | - | - | - | - | - | - | - | - | - | - | - | - | 1 | 0.05 |
| ATTCAAATC | 1 | - | - | - | - | - | - | - | - | - | - | - | - | - | - | - | - | - | 1 | 0.05 |
| ATTCCAAAG | 1 | - | - | - | - | - | - | - | - | - | - | - | - | - | - | - | - | - | 1 | 0.05 |
| ATTGCTTCA | 1 | - | - | - | - | - | - | - | - | - | - | - | - | - | - | - | - | - | 1 | 0.05 |
| ATTGGAGCA | 1 | - | - | - | - | - | - | - | - | - | - | - | - | - | - | - | - | - | 1 | 0.05 |
| ATTTGGTGA | 1 | - | - | - | - | - | - | - | - | - | - | - | - | - | - | - | - | - | 1 | 0.05 |
| ATTTGTGCT | 1 | - | - | - | - | - | - | - | - | - | - | - | - | - | - | - | - | - | 1 | 0.05 |
| ATTTTTGTA | 1 | - | - | - | - | - | - | - | - | - | - | - | - | - | - | - | - | - | 1 | 0.05 |
| CAAACTTCT | 1 | - | - | - | - | - | - | - | - | - | - | - | - | - | - | - | - | - | 1 | 0.05 |
| CAACAGCAA | 1 | - | - | - | - | - | - | - | - | - | - | - | - | - | - | - | - | - | 1 | 0.05 |
| CACTTCAAT | 1 | - | - | - | - | - | - | - | - | - | - | - | - | - | - | - | - | - | 1 | 0.05 |
| CAGCAAGGC | 1 | - | - | - | - | - | - | - | - | - | - | - | - | - | - | - | - | - | 1 | 0.05 |
| CAGCAGCAA | 1 | - | - | - | - | - | - | - | - | - | - | - | - | - | - | - | - | - | 1 | 0.05 |
| CATGTAAGA | 1 | - | - | - | - | - | - | - | - | - | - | - | - | - | - | - | - | - | 1 | 0.05 |
| CCAGGAGGA | - | 1 | - | - | - | - | - | - | - | - | - | - | - | - | - | - | - | - | 1 | 0.05 |
| CCATAACCA | 1 | - | - | - | - | - | - | - | - | - | - | - | - | - | - | - | - | - | 1 | 0.05 |
| CCCTGCTGC | 1 | - | - | - | - | - | - | - | - | - | - | - | - | - | - | - | - | - | 1 | 0.05 |
| CCTCCTTCC | 1 | - | - | - | - | - | - | - | - | - | - | - | - | - | - | - | - | - | 1 | 0.05 |
| CCTTCTTCT | 1 | - | - | - | - | - | - | - | - | - | - | - | - | - | - | - | - | - | 1 | 0.05 |
| CCTTTTATC | 1 | - | - | - | - | - | - | - | - | - | - | - | - | - | - | - | - | - | 1 | 0.05 |
| CGTGCTGGG | 1 | - | - | - | - | - | - | - | - | - | - | - | - | - | - | - | - | - | 1 | 0.05 |
| CTCATCACT | 1 | - | - | - | - | - | - | - | - | - | - | - | - | - | - | - | - | - | 1 | 0.05 |
| CTCCTCTGA | 1 | - | - | - | - | - | - | - | - | - | - | - | - | - | - | - | - | - | 1 | 0.05 |
| CTCTTCTTT | - | 1 | - | - | - | - | - | - | - | - | - | - | - | - | - | - | - | - | 1 | 0.05 |
| CTGCTTTGG | 1 | - | - | - | - | - | - | - | - | - | - | - | - | - | - | - | - | - | 1 | 0.05 |
| CTGTTATTG | 1 | - | - | - | - | - | - | - | - | - | - | - | - | - | - | - | - | - | 1 | 0.05 |
| CTTATTCAC | 1 | - | - | - | - | - | - | - | - | - | - | - | - | - | - | - | - | - | 1 | 0.05 |
| CTTCAGTTT | 1 | - | - | - | - | - | - | - | - | - | - | - | - | - | - | - | - | - | 1 | 0.05 |
| CTTCCTCAA | 1 | - | - | - | - | - | - | - | - | - | - | - | - | - | - | - | - | - | 1 | 0.05 |
| CTTCCTCTT | 1 | - | - | - | - | - | - | - | - | - | - | - | - | - | - | - | - | - | 1 | 0.05 |
| CTTGCTCCA | 1 | - | - | - | - | - | - | - | - | - | - | - | - | - | - | - | - | - | 1 | 0.05 |
| GAAACGGTT | 1 | - | - | - | - | - | - | - | - | - | - | - | - | - | - | - | - | - | 1 | 0.05 |
| GAATAAAAT | 1 | - | - | - | - | - | - | - | - | - | - | - | - | - | - | - | - | - | 1 | 0.05 |
| GACACAATG | 1 | - | - | - | - | - | - | - | - | - | - | - | - | - | - | - | - | - | 1 | 0.05 |
| GATGATGTT | 1 | - | - | - | - | - | - | - | - | - | - | - | - | - | - | - | - | - | 1 | 0.05 |
| GATTTCATT | 1 | - | - | - | - | - | - | - | - | - | - | - | - | - | - | - | - | - | 1 | 0.05 |
| GCAAGCACC | 1 | - | - | - | - | - | - | - | - | - | - | - | - | - | - | - | - | - | 1 | 0.05 |
| GCCACCGGA | 1 | - | - | - | - | - | - | - | - | - | - | - | - | - | - | - | - | - | 1 | 0.05 |
| GCGGCTGCT | 1 | - | - | - | - | - | - | - | - | - | - | - | - | - | - | - | - | - | 1 | 0.05 |
| GCGGGTCAA | 1 | - | - | - | - | - | - | - | - | - | - | - | - | - | - | - | - | - | 1 | 0.05 |
| GCTCAAGCT | 1 | - | - | - | - | - | - | - | - | - | - | - | - | - | - | - | - | - | 1 | 0.05 |
| GCTGCTGTG | 1 | - | - | - | - | - | - | - | - | - | - | - | - | - | - | - | - | - | 1 | 0.05 |
| GGAGAAGAA | 1 | - | - | - | - | - | - | - | - | - | - | - | - | - | - | - | - | - | 1 | 0.05 |
| GGAGAAGGA | 1 | - | - | - | - | - | - | - | - | - | - | - | - | - | - | - | - | - | 1 | 0.05 |
| GGCAGTGAA | 1 | - | - | - | - | - | - | - | - | - | - | - | - | - | - | - | - | - | 1 | 0.05 |
| GGCCTTCTC | 1 | - | - | - | - | - | - | - | - | - | - | - | - | - | - | - | - | - | 1 | 0.05 |
| GGGTTATTG | 1 | - | - | - | - | - | - | - | - | - | - | - | - | - | - | - | - | - | 1 | 0.05 |
| GGTGTGCGA | 1 | - | - | - | - | - | - | - | - | - | - | - | - | - | - | - | - | - | 1 | 0.05 |
| GGTTTTCTA | 1 | - | - | - | - | - | - | - | - | - | - | - | - | - | - | - | - | - | 1 | 0.05 |
| GTCACAGCT | 1 | - | - | - | - | - | - | - | - | - | - | - | - | - | - | - | - | - | 1 | 0.05 |
| GTTCCTCAA | 1 | - | - | - | - | - | - | - | - | - | - | - | - | - | - | - | - | - | 1 | 0.05 |
| GTTCTTGAG | 1 | - | - | - | - | - | - | - | - | - | - | - | - | - | - | - | - | - | 1 | 0.05 |
| GTTGATGTT | 1 | - | - | - | - | - | - | - | - | - | - | - | - | - | - | - | - | - | 1 | 0.05 |
| GTTGGACTT | 1 | - | - | - | - | - | - | - | - | - | - | - | - | - | - | - | - | - | 1 | 0.05 |
| GTTTTTTTT | 1 | - | - | - | - | - | - | - | - | - | - | - | - | - | - | - | - | - | 1 | 0.05 |
| TACAGTTGG | 1 | - | - | - | - | - | - | - | - | - | - | - | - | - | - | - | - | - | 1 | 0.05 |
| TATTTTTTT | 1 | - | - | - | - | - | - | - | - | - | - | - | - | - | - | - | - | - | 1 | 0.05 |
| TCAGTGAAT | 1 | - | - | - | - | - | - | - | - | - | - | - | - | - | - | - | - | - | 1 | 0.05 |
| TCATCATCC | 1 | - | - | - | - | - | - | - | - | - | - | - | - | - | - | - | - | - | 1 | 0.05 |
| TCCAAAACT | 1 | - | - | - | - | - | - | - | - | - | - | - | - | - | - | - | - | - | 1 | 0.05 |
| TCCCCATCC | 1 | - | - | - | - | - | - | - | - | - | - | - | - | - | - | - | - | - | 1 | 0.05 |
| TCCTCCTCA | 1 | - | - | - | - | - | - | - | - | - | - | - | - | - | - | - | - | - | 1 | 0.05 |
| TCCTTCTTA | 1 | - | - | - | - | - | - | - | - | - | - | - | - | - | - | - | - | - | 1 | 0.05 |
| TCGGAAGAA | 1 | - | - | - | - | - | - | - | - | - | - | - | - | - | - | - | - | - | 1 | 0.05 |
| TCTCTCTCA | 1 | - | - | - | - | - | - | - | - | - | - | - | - | - | - | - | - | - | 1 | 0.05 |
| TCTCTCTTT | 1 | - | - | - | - | - | - | - | - | - | - | - | - | - | - | - | - | - | 1 | 0.05 |
| TCTCTTGTA | 1 | - | - | - | - | - | - | - | - | - | - | - | - | - | - | - | - | - | 1 | 0.05 |
| TCTTGTTGC | 1 | - | - | - | - | - | - | - | - | - | - | - | - | - | - | - | - | - | 1 | 0.05 |
| TCTTTCTTT | 1 | - | - | - | - | - | - | - | - | - | - | - | - | - | - | - | - | - | 1 | 0.05 |
| TGAAGTTGA | 1 | - | - | - | - | - | - | - | - | - | - | - | - | - | - | - | - | - | 1 | 0.05 |
| TGACCAGGC | 1 | - | - | - | - | - | - | - | - | - | - | - | - | - | - | - | - | - | 1 | 0.05 |
| TGATTTTGC | 1 | - | - | - | - | - | - | - | - | - | - | - | - | - | - | - | - | - | 1 | 0.05 |
| TGCAGCAAC | 1 | - | - | - | - | - | - | - | - | - | - | - | - | - | - | - | - | - | 1 | 0.05 |
| TGCCTACCC | 1 | - | - | - | - | - | - | - | - | - | - | - | - | - | - | - | - | - | 1 | 0.05 |
| TGCTGCAAT | 1 | - | - | - | - | - | - | - | - | - | - | - | - | - | - | - | - | - | 1 | 0.05 |
| TGGAATAAA | 1 | - | - | - | - | - | - | - | - | - | - | - | - | - | - | - | - | - | 1 | 0.05 |
| TGGCCTGGA | 1 | - | - | - | - | - | - | - | - | - | - | - | - | - | - | - | - | - | 1 | 0.05 |
| TGGCGGCGG | 1 | - | - | - | - | - | - | - | - | - | - | - | - | - | - | - | - | - | 1 | 0.05 |
| TGGCGGCGT | 1 | - | - | - | - | - | - | - | - | - | - | - | - | - | - | - | - | - | 1 | 0.05 |
| TGTTAGCAA | 1 | - | - | - | - | - | - | - | - | - | - | - | - | - | - | - | - | - | 1 | 0.05 |
| TTATCTTCA | 1 | - | - | - | - | - | - | - | - | - | - | - | - | - | - | - | - | - | 1 | 0.05 |
| TTATTGCTT | 1 | - | - | - | - | - | - | - | - | - | - | - | - | - | - | - | - | - | 1 | 0.05 |
| TTCTGCTTC | 1 | - | - | - | - | - | - | - | - | - | - | - | - | - | - | - | - | - | 1 | 0.05 |
| TTCTTCTTT | 1 | - | - | - | - | - | - | - | - | - | - | - | - | - | - | - | - | - | 1 | 0.05 |
| TTCTTTTAG | 1 | - | - | - | - | - | - | - | - | - | - | - | - | - | - | - | - | - | 1 | 0.05 |
| TTCTTTTTT | 1 | - | - | - | - | - | - | - | - | - | - | - | - | - | - | - | - | - | 1 | 0.05 |
| TTGACCCGC | 1 | - | - | - | - | - | - | - | - | - | - | - | - | - | - | - | - | - | 1 | 0.05 |
| TTGATACTT | 1 | - | - | - | - | - | - | - | - | - | - | - | - | - | - | - | - | - | 1 | 0.05 |
| TTGATTCAT | 1 | - | - | - | - | - | - | - | - | - | - | - | - | - | - | - | - | - | 1 | 0.05 |
| TTGGCATGG | 1 | - | - | - | - | - | - | - | - | - | - | - | - | - | - | - | - | - | 1 | 0.05 |
| TTGTTCTCA | 1 | - | - | - | - | - | - | - | - | - | - | - | - | - | - | - | - | - | 1 | 0.05 |
| TTTATCTTT | 1 | - | - | - | - | - | - | - | - | - | - | - | - | - | - | - | - | - | 1 | 0.05 |
| TTTCAAGAA | 1 | - | - | - | - | - | - | - | - | - | - | - | - | - | - | - | - | - | 1 | 0.05 |
| TTTCTTCTT | 1 | - | - | - | - | - | - | - | - | - | - | - | - | - | - | - | - | - | 1 | 0.05 |
| TTTTATTTT | 1 | - | - | - | - | - | - | - | - | - | - | - | - | - | - | - | - | - | 1 | 0.05 |
| TTTTCAATG | 1 | - | - | - | - | - | - | - | - | - | - | - | - | - | - | - | - | - | 1 | 0.05 |
| TTTTCTTTT | 1 | - | - | - | - | - | - | - | - | - | - | - | - | - | - | - | - | - | 1 | 0.05 |
| TTTTTGATT | 1 | - | - | - | - | - | - | - | - | - | - | - | - | - | - | - | - | - | 1 | 0.05 |
| TTTTTTCTT | 1 | - | - | - | - | - | - | - | - | - | - | - | - | - | - | - | - | - | 1 | 0.05 |
| TTTTTTTTC | 1 | - | - | - | - | - | - | - | - | - | - | - | - | - | - | - | - | - | 1 | 0.05 |
| Total | 848 | 215 | 338 | 114 | 78 | 44 | 32 | 11 | 149 | 24 | 17 | 12 | 7 | 3 | 3 | 3 | 0 | 1 | 1899 | 100 |
