# Supplementary material for: First Microsatellite Markers Developed from Cupuassu ESTs: Application in Diversity Analysis and Cross-Species Transferability to Cacao
Source: PLoS One. 2016 Mar 7;11(3):e0151074. doi: 10.1371/journal.pone.0151074 (PMC4780773; doi:10.1371/journal.pone.0151074)
Supplement: S3 Table — (DOCX) [file pone.0151074.s003.docx]

**S3 Table. Resume of the different SSR data set obtained from cacao and cupuassu and already avaliable in the databanks or literature.**

| SSR type | Species | Amount of SSR | Tested SSR | Accession number | Reference |
| --- | --- | --- | --- | --- | --- |
| Genomic | *Theobroma cacao* L. | 23 | 23 | Y16883, Y16977-Y16999 | [[1](#_ENREF_1), [2](#_ENREF_2)] |
| Genomic | *Theobroma cacao* L. | 15 | 15 | nd | [[3](#_ENREF_3)] |
| Genomic | *Theobroma cacao* L. | 201 | 201 | AJ566412-AJ566609 | [[4](#_ENREF_4)] |
| Genomic | *Theobroma cacao* L. | 3^*^ | 3 | nd | [[5](#_ENREF_5)] |
| Genomic | *Theobroma cacao* L. | 123 | 123 | nd | [[6](#_ENREF_6)] |
| Genomic | *Theobroma cacao* L. | 10^*^ | 10 | AJ271823-AJ271826, AJ271945, AJ271947, AJ271950, AJ271953, AJ271956, AJ271957, AJ271959 | [[2](#_ENREF_2), [7](#_ENREF_7)]  [[8](#_ENREF_8)]^**^ |
| EST | *Theobroma cacao* L. | 27 | 27 | nd | [[9](#_ENREF_9)] |
| EST | *Theobroma cacao* L. | 409^*^ | 409 | nd | [[10](#_ENREF_10), [11](#_ENREF_11)]^**^ |
| EST | *Theobroma cacao* L. | 11 | 11 | AM117760-AM117768, DN237949-DN237957,  CK144293-CK144298, CF972636–CF974749, CA794213–CA798660 | [[12](#_ENREF_12)] |
| EST | *Theobroma cacao* L. | 505 | 49 | AM851096-AM851106^***^ | [[13](#_ENREF_13)] |
| EST | *Theobroma cacao* L. | 251 | 251 | nd | [[14](#_ENREF_14)] |
| EST | *Theobroma cacao* L. | 428 | 33 | nd | [[15](#_ENREF_15)] |
| EST | *Theobroma grandiflorum* | 498 | 77 | nd | This study |
| Total | | 2504 | 1232 | - | - |

* Only new SSR in relation to previous studies were considered

** Obtained and analyzed in different works

*** Some of the SSR were described in the corresponding paper but not submitted in the NCBI/EMBL databanks

**References:**

1. Lanaud C, Risterucci AM, Pieretti I, Falque M, Bouet A, Lagoda PJL. Isolation and characterization of microsatellites in *Theobroma cacao* L. Molecular Ecology. 1999;8(12):2141-3. doi: 10.1046/j.1365-294x.1999.00802.x.

2. Saunders JA, Mischke S, Leamy EA, Hemeida AA. Selection of international molecular standards for DNA fingerprinting of Theobroma cacao. Theoretical and Applied Genetics. 2004;110(1):41-7. doi: 10.1007/s00122-004-1762-1.

3. Kuhn DN, Heath M, Wisser RJ, Meerow A, Brown JS, Lopes U, et al. Resistance gene homologues in Theobroma cacao as useful genetic markers. TAG Theoretical and Applied Genetics. 2003;107(2):191-202. doi: 10.1007/s00122-003-1239-7.

4. Pugh T, Fouet O, Risterucci AM, Brottier P, Abouladze M, Deletrez C, et al. A new cacao linkage map based on codominant markers: development and integration of 201 new microsatellite markers. Theoretical and Applied Genetics. 2004;108(6):1151-61. doi: 10.1007/s00122-003-1533-4.

5. Brown J, Schnell R, Motamayor J, Lopes U, Kuhn D, Borrone J. Resistance gene mapping for witches’ broom disease in *Theobroma cacao* L. in an F2 population using SSR markers and candidate genes. J Amer Soc Hort Sci. 2005;130:366-73.

6. Araújo IS, Intorne AC, Pereira MG, Lopes UV, Filho GAdS. Development and characterization of novel tetra-, tri- and di-nucleotide microsatellite markers in cacao (Theobroma cacao L.). Molecular Breeding. 2007;20(1):73-81. doi: 10.1007/s11032-006-9057-7.

7. Risterucci MA, Grivet L, N’Goran KJA, Pieretti I, Flament HM, Lanaud C. A high-density linkage map of Theobroma cacao L. Theoretical and Applied Genetics. 2000;101(5):948-55. doi: 10.1007/s001220051566.

8. Alves RM, Sebbenn AM, Artero AS, Figueira A. Microsatellite loci transferability from *Theobroma cacao* to *Theobroma grandiflorum*. Molecular Ecology Notes. 2006;6(4):1219-21. doi: 10.1111/j.1471-8286.2006.01496.x.

9. Borrone JW, Brown JS, Kuhn DN, Motamayor JC, Schnell RJ. Microsatellite markers developed from Theobroma cacao L. expressed sequence tags. Molecular Ecology Notes. 2007;7(2):236-9. doi: 10.1111/j.1471-8286.2006.01561.x.

10. Argout X, Fouet O, Wincker P, Gramacho K, Legavre T, Sabau X, et al. Towards the understanding of the cocoa transcriptome: Production and analysis of an exhaustive dataset of ESTs of *Theobroma cacao* L. generated from various tissues and under various conditions. BMC Genomics. 2008;9(1):512. PubMed PMID: doi:10.1186/1471-2164-9-512.

11. Allegre M, Argout X, Boccara M, Fouet O, Roguet Y, Bérard A, et al. Discovery and mapping of a new expressed sequence tag-single nucleotide polymorphism and simple sequence repeat panel for large-scale genetic studies and breeding of Theobroma cacao L. DNA Research. 2012;19(1):23-35. doi: 10.1093/dnares/dsr039.

12. Riju A, Rajesh MK, Sherin PT, Chandrasekar A, Apshara SE, Arunachalam V. Mining of expressed sequence tag libraries of cacao for microsatellite markers using five computational tools. Journal of Genetics. 2009 88(2):217-25.

13. Lima LS, Gramacho KP, Pires JL, Clement D, Lopes UV, Carels N, et al. Development, characterization, validation, and mapping of SSRs derived from *Theobroma cacao* L.–*Moniliophthora perniciosa* interaction ESTs. Tree Genetics & Genomes. 2010;6(5):663-76. doi: 10.1007/s11295-010-0282-1.

14. Fouet O, Allegre M, Argout X, Jeanneau M, Lemainque A, Pavek S, et al. Structural characterization and mapping of functional EST-SSR markers in Theobroma cacao. Tree Genetics & Genomes. 2011;7(4):799-817. doi: 10.1007/s11295-011-0375-5.

15. Santos R, Clement D, Lemos L, Legravre T, Lanaud C, Schnell R, et al. Identification, characterization and mapping of EST-derived SSRs from the cacao–*Ceratocystis cacaofunesta* interaction. Tree Genetics & Genomes. 2013;9(1):117-27. doi: 10.1007/s11295-012-0539-y.
